# Merging rhodium-catalysed C–H activation and hydroamination in a highly selective [4+2] imine/alkyne annulation

**DOI:** 10.1038/ncomms11506

**Published:** 2016-06-20

**Authors:** Rajith S. Manan, Pinjing Zhao

**Affiliations:** 1 Department of Chemistry and Biochemistry, North Dakota State University, Fargo, North Dakota 58102, USA

## Abstract

Catalytic C–H activation and hydroamination represent two important strategies for eco-friendly chemical synthesis with high atom efficiency and reduced waste production. Combining both C–H activation and hydroamination in a cascade process, preferably with a single catalyst, would allow rapid access to valuable nitrogen-containing molecules from readily available building blocks. Here we report a single metal catalyst-based approach for N-heterocycle construction by tandem C–H functionalization and alkene hydroamination. A simple catalyst system of cationic rhodium(I) precursor and phosphine ligand promotes redox-neutral [4+2] annulation between N–H aromatic ketimines and internal alkynes to form multi-substituted 3,4-dihydroisoquinolines (DHIQs) in high chemoselectivity over competing annulation processes, exclusive *cis*-diastereoselectivity, and distinct regioselectivity for alkyne addition. This study demonstrates the potential of tandem C–H activation and alkene hydrofunctionalization as a general strategy for modular and atom-efficient assembly of six-membered heterocycles with multiple chirality centres.

## Abstract (web summary)

C–H activations and hydroaminations are useful synthetic procedures for building up complex, functional molecules. Here, the authors report a single catalyst capable of performing tandem C–H activation and hydroamination, giving access to N-heterocycles via the [4+2] annulation of aromatic imines and alkynes.

## Graphical abstract

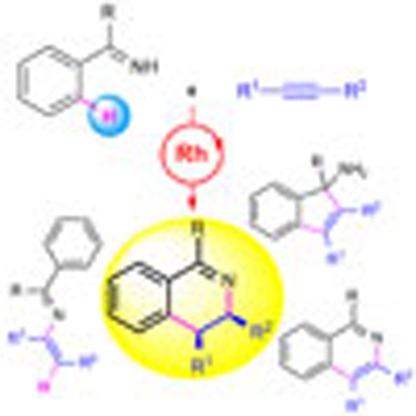

In recent years, transition metal-mediated C–H bond activation has been increasingly explored in catalytic construction of heterocycles that involve tandem formation of carbon–carbon and carbon–heteroatom bonds^1^,^2^,^3^,^4^,^5^,^6^,^7^,^8^. A major strategy for such catalytic heterocycles synthesis via C–H activation is the intermolecular coupling between aromatic compounds and alkynes to form six-membered benzoheterocycles (Fig. 1a). These [4+2] annulations utilize a variety of heteroatom-based *ortho*-directing groups for aromatic C–H bond activation. The resulting metallacycle intermediates (**A**) undergo subsequent alkyne coupling and ring-closure steps to incorporate the directing groups into the heterocyclic product backbone^9^,^10^,^11^. A dominant majority of these domino processes occur in the form of oxidative annulation that retains a carbon–carbon double bond in the heterocycle structure (Fig. 1b)^8^,^12^,^13^,^14^,^15^,^16^,^17^,^18^,^19^,^20^,^21^,^22^,^23^,^24^,^25^,^26^,^27^,^28^,^29^,^30^,^31^,^32^,^33^,^34^. For heteroatom-based directing groups with a N–H or O–H moiety, stoichiometric amounts of Ag(I) or Cu(II) oxidants are commonly used for oxidative [4+2] annulations. This need for external oxidants can be eliminated either by developing aerobic oxidation^25^,^26^,^27^,^34^ or dehydrogenative coupling conditions^30^, or by using an ‘oxidizing directing group' as the internal oxidant that releases a small molecule byproduct such as water^17^,^21^, alcohol^18^ or carboxylic acid^23^,^33^. In principle, redox-neutral [4+2] annulations with aromatic compounds and alkynes would provide direct access to partially saturated benzoheterocycles in 100% atom efficiency and forms up to two new chirality centres (Fig. 1a). However, this strategy was only demonstrated in a single report in 2013 by Sun, Wang and coworkers^35^, who developed a Re(I)–Mg(II) bimetallic catalyst for benzamide/alkyne coupling to synthesize 3,4-dihydroisoquinolinones with controlled *cis*- or *trans*-diastereoselectivity (Fig. 1c). This redox-neutral [4+2] annulation featured a ring-closure step of Mg-catalysed intramolecular alkene addition by the amide N–H bond, which represents an example of main group metal-catalysed alkene hydroamination^36^.

Considering that late transition metal catalysts have been widely used in both C–H activation and alkene hydroamination processes^1^,^36^,^37^, we envisioned that redox-neutral [4+2] annulations for N-heterocycle synthesis may be promoted by a single transition metal catalyst via a domino sequence of C–H bond activation, C–C bond formation by alkyne coupling and C–N bond formation by intramolecular alkene hydroamination. This ‘one catalyst does it all' approach for redox-neutral annulations would complement existing methods with operationally simple procedures and provide opportunities for ligand-enabled control over chemo- and stereoselectivity for hydroamination. From the reaction mechanism perspective, our strategy can be compared with ruthenium- and gold-catalysed synthesis of 1,2-dihydroquinoline derivatives by 3-component coupling between an aromatic amine and two alkynes^38^,^39^,^40^. This redox-neutral [3+2+1] annulation involves a tandem sequence of intermolecular alkyne hydroamination and aromatic C–H functionalization. In the current study, we have focused our attention on metal-catalysed intermolecular couplings between N–H aromatic ketimines and internal alkynes (Fig. 1d). We and others have previously reported three classes of couplings between these two reaction partners to selectively form isoquinolines by oxidative [4+2] N-heterocyclization^15^,^30^, indene-based tertiary carbinamines by [3+2] carbocyclization^41^,^42^,^43^, and 2-aza-1,3-butadienes by alkyne hydroamination with N–H imine nucleophile (hydroimination)^44^. These divergent catalytic processes attest to the challenge of targeting desired redox-neutral [4+2] annulation with high chemoselectivity.

We herein describe a mechanism-based development of a rhodium-catalysed redox-neutral [4+2] annulation with aromatic N–H ketimines and internal alkynes to form 3,4-dihydroisoquinolines (DHIQs), which are synthetic intermediates towards valuable 1,2,3,4-tetrahydroisoquinoline (THIQ) structures^45^. The strategic combination of a cationic Rh(I) catalyst precursor and a bis(phosphine) ligand enables this N-heterocyclization to proceed with high chemoselectivity over other possible coupling processes and exclusive diastereoselectivity for *cis*-3,4-disubstituted products. Regio- and stereochemistry results, as well as results from deuterium-labelling studies, are most consistent with a domino sequence of imine-directed aromatic C–H bond activation via oxidative addition, alkyne coupling, and a novel intramolecular alkene hydroimination. Synthetic utility of target DHIQ products are demonstrated with several stoichiometric and catalytic transformations including diastereoselective hydride reduction and Rh(III)-mediated regioselective C–H functionalizations.

## Results

### Initial observations and mechanistic implications

**Figure 2:**
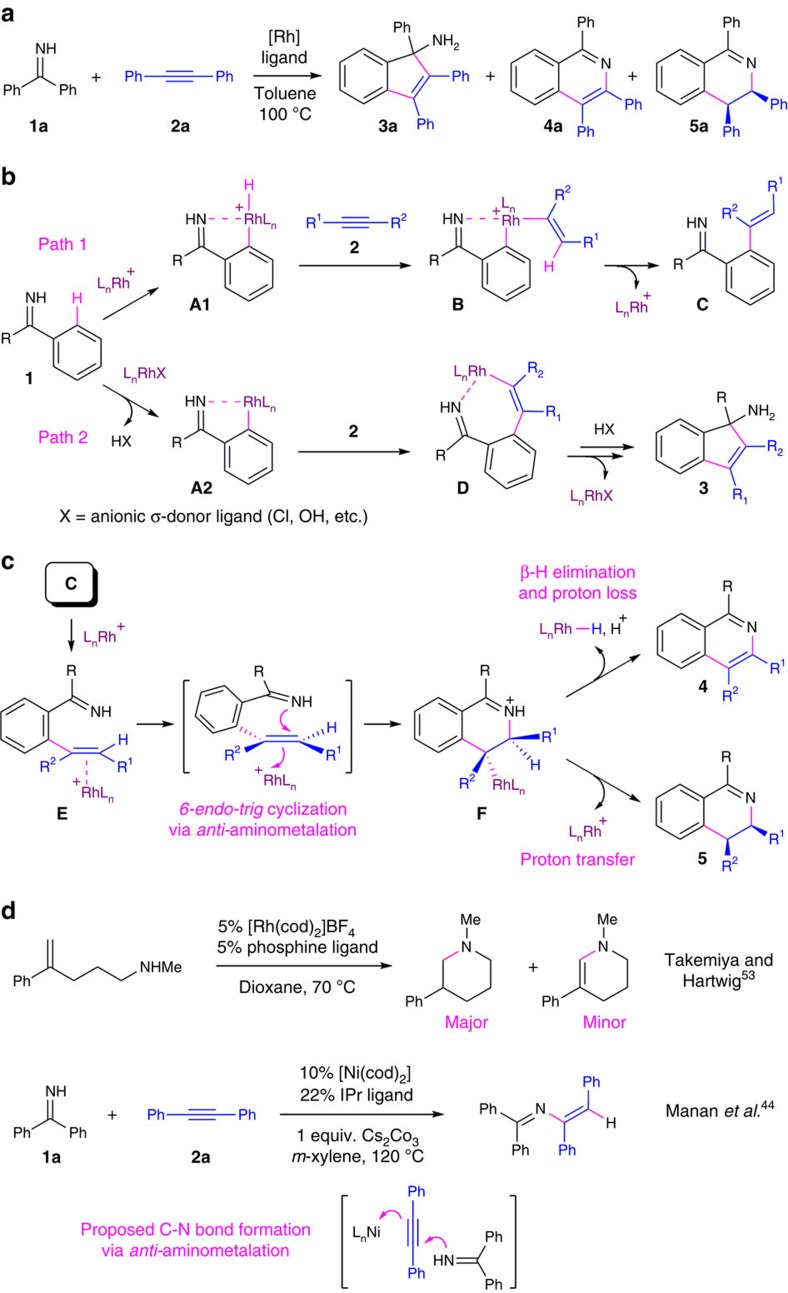
Catalyst design for redox-neutral [4+2] imine/alkyne coupling. (**a**) Observation of three different annulation products with Rh(I) catalysts. (**b**) Proposed pathways for imine-directed aromatic C–H bond activation and subsequent alkyne coupling. (**c**) Proposed N-heterocyclization by intramolecular alkene hydroimination and subsequent competition between oxidative and redox-neutral [4+2] annulation product formations. (**d**) Mechanistically related reports on intramolecular alkene hydroamination and intermolecular alkyne hydroimination.

Our study began with the detection of three annulation products from catalytic coupling between N–H aromatic ketimines and internal alkynes (Scheme 2a). In particular, Rh(I)-catalysed 1:1 coupling between benzophenone imine (**1a**) and diphenylacetylene (**2a**) led to formation of [3+2] carbocyclization product **3a** (refs 41, 42, 43), oxidative [4+2] N-heterocyclization product **4a** (refs 15, 30), and the desired dihydroisoquinoline product **5aa** by redox-neutral [4+2] N-heterocyclization. The overall yield and chemoselectivity depended significantly on the choices of Rh(I) catalyst precursor, ancillary ligand and solvent for the reaction (*vide infra*). Thus, the major challenge for our catalyst development was to selectively promote formation of **5aa** over byproducts **3a** and **4a**. In particular, we expected that formation of isoquinoline byproduct **4a** would be highly competitive due to the strong thermodynamic driving force of aromatization. To this end, our effort was guided by an early observation that cationic Rh(I) precursors with non-coordinating counteranions, for example, [Rh(cod)_2_]BF_4_ (**6**), appeared to promote higher chemoselectivity for **5aa** than neutral Rh(I) precursors with anionic ligands such as [Rh(cod)_2_(OH)]_2_. In addition, **5aa** was detected exclusively as the *cis*-3,4-diphenyl diastereomer by ^1^H NMR spectroscopy with a relatively small H(3)-H(4) coupling in ^1^H NMR (^3^*J*∼6.0 Hz), which supported gauche *cis*- H(3)-H(4) and gauche *cis*-3,4-disubstitution relationships^30^,^43^,^46^. Combined with proposed reaction pathways for mechanistically relevant annulation processes^8^,^15^,^30^,^35^,^41^,^42^,^43^, these results led us to suggest an imine-directed C–H oxidative addition on cationic Rh(I)^47^,^48^,^49^ to form a cyclometalated Rh(III) hydride **A1** (Fig. 2b, Path 1; see Supplementary Fig. 88 for a more detailed discussion)^9^. Alkyne insertion into the Rh–H linkage and subsequent C–C reductive elimination gave an alkyne hydroarylation product **C**, which we envisioned as a key intermediate for [4+2] annulation products (*vide infra*)^47^,^48^,^49^. Alternatively, a neutral Rh(I) catalyst precursor may promote a deprotonation-type C–H activation pathway, leading to a cyclometalated Rh(I) complex **A2** (Path 2). Subsequent alkyne insertion into the Rh–C bond gave an imine-chelated Rh(I) alkenyl complex **D** (refs 10, 11), which could lead to [3+2] carbocyclization product **3** by sequential intramolecular imine insertion into the Rh-alkenyl linkage and protonation of the resulting Rh(I) alkyl complex^41^,^42^,^43^.

### Mechanism-based catalyst design

The proposed acyclic intermediate **C** was not directly detected during our study. However, the involvement of similar alkyne hydroarylation intermediates was confirmed by Sun and Wang^35^ in their recent report on Re(I)–Mg(II) bimetallic catalyst for redox-neutral [4+2] benzamide/alkyne annulation. In addition, Bergman, Ellman and coworkers have reported structurally analogous acyclic C–H alkenylation products in their reports on Rh(I)-catalysed N-heterocyclization with α,β-unsaturated N-benzyl imines and alkynes, which undergoes non-catalytic 6π-electrocyclization to form dihydropyridines as reactive intermediates for several one-pot procedures towards pyridine and tetrahydropyridine synthesis^49^,^50^,^51^. Aiming for a single catalyst-based domino procedure, we hypothesized that a cationic Rh(I) catalyst for the stage of C–H activation and alkyne coupling (Fig. 2b, Path 1) could also promote subsequent ring closure by intramolecular alkene hydroamination. As described in Fig. 2c, alkene π-complexation between **C** and a Lewis acidic Rh(I) centre would promote intramolecular nucleophilic attack by the N–H imine moiety in stereospecific *6-endo-trig* fashion to give metal alkyl intermediate **F**. With the stereochemistry of C–N bond formation determined by such *anti*-aminometalation^36^,^37^, **F** could undergo either *syn*-β-H elimination to give isoquinoline **4** or protonation of the Rh-alkyl linkage with stereospecific retention to give the *cis*-isomer of redox-neutral annulation product **5** (see Supplementary Fig. 88 for a more detailed description). Thus, we envisioned that an ideal catalyst system for selective synthesis of DHIQs should effectively promote a tandem sequence of C–H alkenylation (**1**→**C**), intramolecular alkene hydroamination (**C**→**F**), and selective protonation of a Rh-alkyl complex (**F**→**5**) over β-H elimination. This catalysis design is inspired by cationic Rh(I) catalysts that have been successfully explored by the Hartwig group for inter- and intramolecular alkene hydroamination^52^,^53^,^54^,^55^. For example, [Rh(cod)_2_]BF_4_ (**6**) was demonstrated as an effective catalyst precursor for *anti*-Markovnikov intramolecular hydroamination of vinylarenes with secondary aliphatic amines (Fig. 2d)^53^. Notably, selective formation of desired hydroamination products versus oxidative amination products in this report was significantly affected by the choice of chelating bis-phosphine ligands. Such ligand-controlled chemoselectivity can be attributed to the ligand bite angle effects on β-H elimination versus protonation of metal alkyl intermediates in several catalytic processes including alkene hydroamination versus oxidative amination, as well as Heck–Mizoroki olefinations versus the corresponding alkylation processes^56^. It is also noteworthy that we have recently reported a nickel-catalysed intermolecular alkyne hydroamination with N–H aromatic ketimines that proceeded by a proposed imine nulcleophilic attack on Ni(0)-coordinated alkyne for stereospecific *anti*-addition^44^. This result serves as an indirect evidence to support both the unconventional role of N–H imine moiety as a N-nucleophile for hydroamination and the proposed stereochemistry for C–N bond formation in current study.

### Catalyst development

With mechanistic insights described above, we have focused our attention on the combination of cationic Rh(I) precursors and chelating bis-phosphine ligands for catalyst development (Table 1). A particularly effective catalyst system was discovered using [Rh(cod)_2_]BF_4_ precursor (**6**) and DPEphos ligand (bis[(2-diphenylphosphino)phenyl] ether, **7**). It is noteworthy that DPEphos is a prominent example of bis-phosphine ligands with wide bite angles, whose significant ligand effects on chemo- and regioselectivity in metal-catalysed coupling reactions including hydroamination are well documented^52^,^56^. At 100 °C and in toluene solvent, a 1:1 coupling between **1a** and **2a** was promoted by 5 mol% [Rh(cod)_2_]BF_4_ (**6**) and 6 mol% DPEphos (**7**) to selectively form **5aa** in 93% yield over 24 h. Under these conditions, oxidative [4+2] annulation product **4a** was formed in 3% yield, and the [3+2] carbocyclization product **3a** was not detected (entry 1). Significantly reduced reactivity and chemoselectivity was observed when replacing DPEphos with various bis- and mono-phosphines (entries 2–8), or replacing **6** with several other Rh(I) catalyst precursors (entries 9–12). Switching from toluene to THF solvent led to similar overall reactivity but slightly lower selectivity for **5aa** (entry 13), while much lower reactivity and chemoselectivity was observed in hexane (entry 14) and several solvents of higher polarity (entries 15–17). Lastly, using an increased amount of DPEphos ligand (entry 18) or reduced catalyst loading (entry 19) both led to lower combined yield of **5aa** and **4a** but higher yield for **4a** formation. This observation suggested that the oxidative [4+2] annulation may also proceed in different pathways that involve non-catalytic ring-closure steps such as 6π-electrocyclization (see Supplementary Fig. 88 for a more detailed discussion on possible pathways for imine/alkyne coupling).

### Alkyne substrate scope

With the standard reaction conditions established, various aromatic N–H ketimine (**1**) and internal alkyne (**2**) substrates were studied for Rh(I)-catalysed redox-neutral [4+2] annulation (Scheme 3). In general, 1,3,4-trisubstiuted DHIQs (**5**) were formed in exclusive *cis*-3,4-diastereoselectivity and high chemoselectivity, with only trace amounts (0–5%) of isoquinoline byproducts (**4**) and no detection of indenamine byproducts (**3**) as evidenced by gas chromatography (GC) and ^1^H NMR analysis of the unpurified reaction mixture. However, several DHIQ products appeared to undergo spontaneous dehydrogenation during the separation and purification procedures, which generated small amounts of isoquinoline byproducts **4** that could not be fully removed from the isolated DHIQ products **5** (*vide infra*). Scope of the alkyne substrates was studied with benzophenone imine (**1a**) as the reaction partner, and high coupling yields were achieved with various symmetrical alkynes having aryl, 2-thienyl, and alkyl substituents (products **5aa**–**5am**). However, no coupling products were detected for bis(2-pyridyl)acetylene or terminal alkynes such as phenylacetylene. Reactions with non-symmetrical phenyl alkyl alkynes led to the exclusive formation of *cis*-3-alkyl-4-phenyl products (**5an**–**5ap**). By sharp contrast, most reported methods of oxidative [4+2] heterocyclization with non-symmetrical aryl alkyl akynes displayed the opposite regioselectivity^8^,^12^,^13^,^14^,^15^,^16^,^17^,^18^,^19^,^20^,^21^,^22^,^23^,^24^,^25^,^26^,^27^,^28^,^29^,^30^,^31^,^32^,^33^,^34^. For instance, regioselective formation of a 3-aryl-4-alkyl-substituted isoquinoline product was recently reported by the Wang group by manganese-catalysed dehydrogenative [4+2] annulation of N–H imines and alkynes^30^. The only example of similar regioselectivity with aryl alkyl alkynes was reported by the Cramer group on rhodium(III)-catalysed oxidative [4+2] annulation of N-acyl arylsulfonamides and alkynes, which promoted formation of a 3-alkyl-4-aryl-substituted benzosultam product in modest regioselectivity (2:1) (ref. 27). The reaction with 1-(2-thienyl)-2-phenylacetylene generated a 4:1 mixture of regioisomers, and the major isomer of 3-phenyl-4-(2-thienyl)-substituted DHIQ product **5aq** was isolated in 66% yield. This modest regioselectivity was likely affected by potential coordination between sulfur centre of the 2-thienyl moiety and the cationic Rh(I) centre during the akyne coupling process. Due to product decomposition by dehydrogenation, isolated products **5am**–**5ao** were contaminated with ∼10–15% of the corresponding isoquinoline byproducts, and the reported yields were estimated by ^1^H NMR analysis (see Supplementary Figs 29, 31 and 33 for more details).

### N–H ketimine substrate scope

Scope of the ketimine substrates was studied by coupling with diphenylacetylene (**2a**), and high reactivity was observed for both diaryl and aryl alkyl ketimines with phenyl groups (**5ia** and **5ja**) or electron-poor aryl groups having F, Cl and CF_3_ groups at *para* or *meta* positions (**5ba**, **5ca**, **5fa** and **5ka**–**5oa**). Electron-rich di(*p*-tolyl) N–H ketimine gave product **5da** in 70% yield, while di(*p*-anisyl) N–H ketimine failed to react with **2a** to give detectable coupling products. Such electronic effect on ketimine reactivity was further demonstrated with product **5ga**, which was formed with exclusive regioselectivity for C–H functionalization of the electron-poor aryl group with *meta*-CF_3_ substituent over the electron-rich one with *meta*-methoxy substituent. Notably, the sterically hindered di(*o*-tolyl) N–H ketimine did react with **2a** to give redox-neutral [4+2] adduct **5ha**. Product decomposition via dehydrogenation was also observed for **5ha**, and an isolated yield of 66% was calculated based on ^1^H NMR analysis (see Supplementary Fig. 54 for details). The solid-state structures of products **5aa**, **5an** and **5na** were established by single-crystal X-ray diffraction analysis, which confirmed the *cis*-diastereoselectivity and 3-alkyl-4-aryl regioselectivity for corresponding DHIQ products.

## Discussion

Current results of the substituent effects on coupling reactivity and regioselectivity provide several mechanistic insights that were consistent with the proposed pathway for C–H alkenylation (Fig. 2b, Path 1). Firstly, the lack of significant reactivity dependence on symmetrically substituted alkyne substrates (products **5aa**–**5am**) suggested that the proposed C–C and C–N bond formation steps (**B**→**C**, **E**→**F**) were not rate determining. Secondly, the highly regioselective functionalization at the more electron-deficient aryl moiety in product **5ga** suggested a C–H activation pathway that does not involve electrophilic aromatic substitution^1^. Instead, these restuls were consistent with a proposed rate-determining step of C–H oxidative onto the Rh(I) centre of the catalyst (**1**→**A1**) (ref. 49). Thirdly, the distinct regioselectivity with non-symmetrical phenyl alkyl alkynes (products **5an**–**5ap**) was consistent with proposed alkyne insertion into a Rh–H linkage, which placed the Rh centre preferentially at the more stabilized, α-to-phenyl position in intermediate **B** (R_1_=alkyl, R_2_=phenyl). Subsequent C–C reductive elimination gave Murai-type hydroarylation product **C** (refs 47, 48, 49), whose regiochemistry was reflected in the 3-alkyl-4-phenyl substitution pattern of the corresponding DHIQ product **5**.

**Figure 4:**
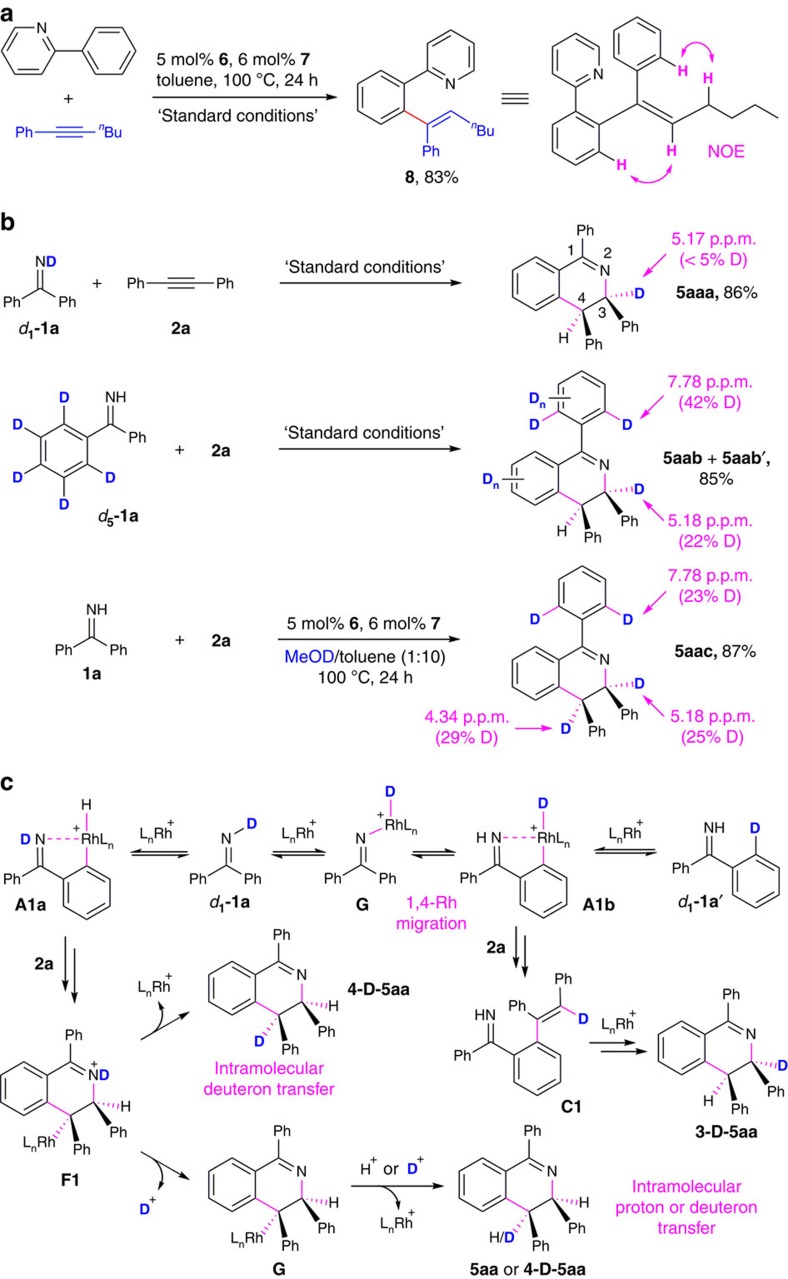
Reaction mechanism studies and analysis. (**a**) Regioselective alkyne hydroarylation with 2-phenylpyridine under standard catalytic conditions for [4+2] imine/alkyne annulation. (**b**) Results from deuterium-labelling studies. (**c**) Proposed pathways for regioselective deuterium transfer and equilibrium processes for H/D scrambling.

To further investigate regiochemistry of the proposed C–H alkenylation intermediate **C**, the current catalyst system was explored for a 1:1 coupling between 2-phenylpyridine and *n*-butylphenylacetylene that should not give an annulation product without oxidants ([Fig f3])(Fig. 4a)^10^,^57^. Under the standard conditions for catalytic redox-neutral [4+2] annulations, a (*E*)-1,1-diarylhexene product **8** was acquired in 83% yield and with exclusive regio- and stereoselectivity (see Supplementary Figs 75 and 77 for 1H NMR and NOESY spectra). This result is consistent with our proposed C–H alkenylation pathway and represents a relatively rare example of high selectivity towards 1,1-diarylalkene regioisomers for catalytic hydroarylation with aryl alkyl alkynes by the directed C–H activation strategy^21^,^58^. Lastly, the failure of produt formation for the highly electron-rich imine substrate di(p-anisyl) N–H ketimine (product **5ea**) suggested that C–H activation may be inhibited by strong σ-complexation between Rh(I) and imine ligands. A similar lack of reactivity for electron-rich N–H aromatic imines was observed in our previous study on Ni-catalysed hydroimination of alkynes^44^.

To better understand hydrogen atom transfer processes in the proposed mechanism for redox-neutral [4+2] annulation (Fig. 2b,c), we carried out deuterium-labelling studies on the formation of **5aa** by coupling between **1a** and **2a** under standard catalytic conditions and with various deuterium sources (Fig. 4b). Firstly, the reaction with Ph_2_C=ND (***d***_**1**_**-1a**) led to <5% D incorporation at C3 position of the product **5aaa**. The significant deuterium loss suggested a rapid hydrogen/deuterium (H/D) srambling between the imine moiety and the reaction media, presumably due to traces of moisture or acid impurities. The regioselective D-transfer to C3, albeit in low conversion, suggested an intramolecular H/D exchange between the imine N centre and the *ortho* aromatic positions of ***d***_**1**_**-1a**. The resulting *ortho*-D atom would migrate to C3 position of **5aa** by the proposed pathways for C–H alkenylation (Fig. 2b) and intramolecular alkene hydroamination (Fig. 2c). Secondly, the reaction with (C_6_D_5_)PhC=NH (***d***_**5**_**-1a**) led to an inseparable mixture of products **5aab** and **5aab′** via imine-directed C–H or C–D activation. This mixture displayed 22% D incorporation at C3 position, while 42% D could be measured at each *ortho*-position of the 1-phenyl group. This result further supported the proposed H/D exchange between the N centre and *ortho* aromatic positions of imine substrates. Thirdly, the reaction with non-deuterated **1a** and in a mixed solvent of 1:10 MeOD/toluene gave **5aac** with 25% D at C3, 29% D at C4 and 23% D at each *ortho*-position of the 1-phenyl group. The partial deuterium incorporation at C4 suggested that the cleavage of Rh-alkyl linkage (Fig. 2c, **F**→**5**) likely occurred by both intra- and intermolecular proton/deuterium transfer processes.

Despite the complication by facile H/D scrambling, results from these deuterium-labelling experiments allowed us to gain further evidence and additional details for proposed reaction pathways as described in Fig. 4c. The proposed H/D exchange between the imine N atom and *ortho* aromatic positions likely occurs by reversible N–H(D) oxidative addition onto Rh(I) centre of the catalyst to form a Rh(III) hydrido iminyl intermediate (***d***_**1**_**-1a**→**G**), which undergoes reversible 1,4-Rh migration^59^ to activate an *ortho* aromatic C–H bond and form the cyclometalated intermediate **A1b** with a Rh–D linkage. Subsequent C–D reductive elimination generates *ortho*-deuterated imine (***d***_**1**_**-1a′**) and completes the proposed H/D exchange. Notably, the cyclometalated Rh(III) intermediate with a Rh–D linkage that is analogous to **A1b** is also formed by imine-directed *ortho* C–D activation (that is, microscopic reverse of C–D reductive elimination) with ***d***_**5**_**-1a** (Fig. 2b). As described in Path 1 in Fig. 2b, a tandem sequence of alkyne insertion with **A1b** (or its analogue from ***d***_**5**_**-1a**) and C–C reductive elimination leads to regioselective D-transfer onto the mono-substituted alkenyl position in alkyne hydroarylation product **C1**, which followed the proposed hydroamination pathway (Fig. 2c, **C**→**5**) to form the 3-deuterated compound **3-D-5aa** as observed in products **5aaa**–**5aac**. Besides the proposed intramolecular H/D exchange (***d***_**1**_**-1a**→***d***_**1**_**-1a′**), imine-directed *ortho* C–H oxidative addition forms the cyclometalated intermediate **A1a** that transforms into intermediate **F1** after the proposed alkyne coupling and ring-closure steps. With the D atom retained on the iminium N centre in **F1**, cleavage of the Rh-alkyl linkage by intramolecular deuteron transfer in stereospecific retention forms the 4-deuterated compound **4-D-5aa** as observed in product **5aac**. Alternatively, deuteron dissociation from **F1** and subsequent cleavage of the Rh-alkyl linkage by intermolecular proton or deuteron transfer forms the non-deuterated **5aa** or **4-D-5aa** respectively. The observed facile H/D scrambling can be attributed to the Bronsted acid behaviours by several proposed reactive intermediates such as cationic Rh(III) hydride/deuteride complexes (**A1a**, **A1b** and **G**) and the iminium species (**F1** and its protonated analogue), which could undergo fast and reversible H/D transfer with external proton or deuteron sources such as trace moisture, acid, or added MeOD in the reaction environment.

**Figure 5:**
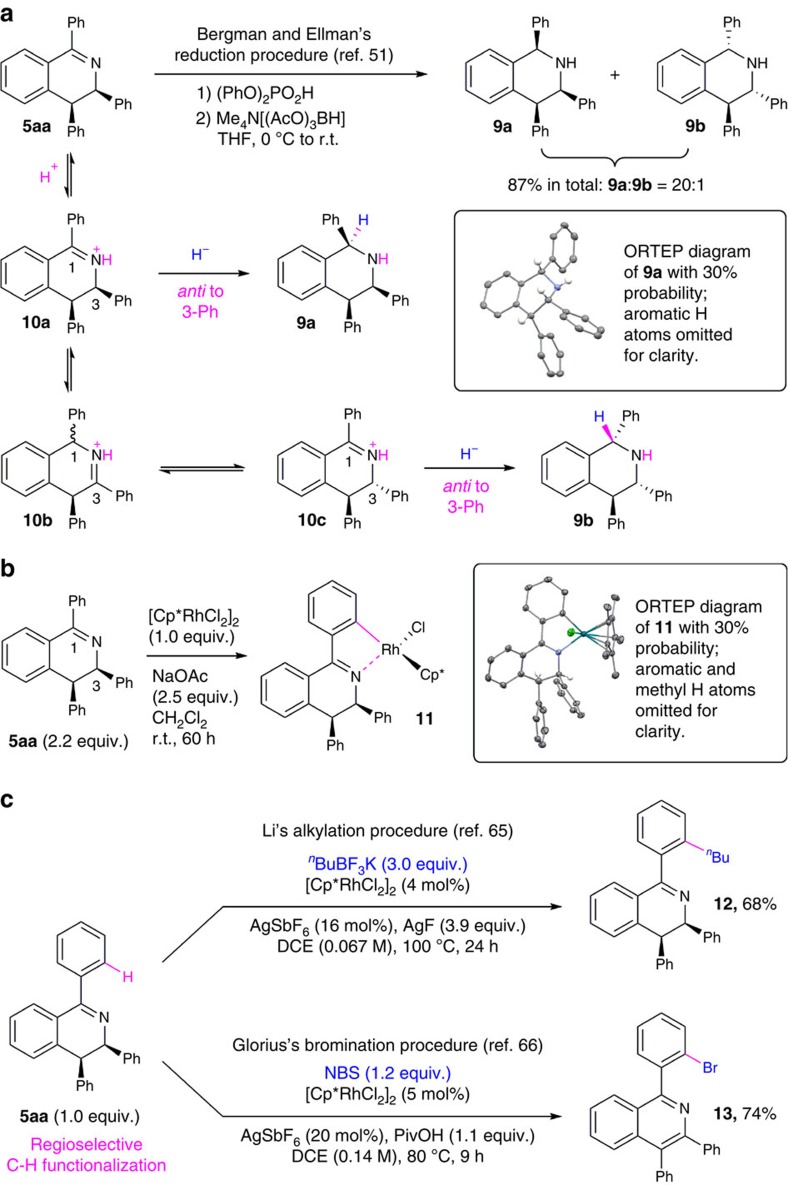
Demonstration of synthetic transformations of DHIQ products with the model compound 5aa. (**a**) Diastereoselective hydride reduction. (**b**) Rh(III)-mediated regioselective cyclometalation. (**c**) Rh(III)-catalyzed aromatic functionalizations via directed C–H activation.

Product **5aa** was subjected to several stoichiometric and catalytic transformations to explore DHIQ products from the current study as valuable building blocks in chemical synthesis (Fig. 5). In particular, we expected that hydrogenation or nucleophilic addition of the imine moiety in these DHIQ products could lead to stereoselective formation of the corresponding THIQs, which are important structural motifs in biologically active compounds including natural alkaloids and drug molecules^45^. Thus, we studied the hydride reduction of **5aa** using a procedure reported by Bergman and Ellman (Fig. 5a)^51^. Upon acid-mediated activation, **5aa** underwent a borohydride reduction to give a 20:1 mixture of two diastereomers of the corresponding THIQ product in 87% overall yield. ^1^H NMR and X-ray crystallography indicated that the major product was the all-*cis*-diastereomer (**9a**), while the minor stereoisomer **9b** displayed *cis*-1,3 and *trans*-3,4 stereochemistry. The *cis*-1,3 relationship in both isomers should result from stereospecific, *anti* to 3-phenyl hydride transfer^51^. The formation of **9b** was likely due to acid-mediated epimerization at C3 via iminium intermediates^60^,^61^. This result highlights the pontential of redox-neutral [4+2] imine/alkyne annulation as a new approach towards stereoselective synthesis of poly-substituted THIQs that complements existing strategies such as intramolecular electrophilic aromatic substitutions^62^ or metal-catalysed enantioselective hydrogenation of isoquinolines^63^,^64^.

To explore 1-aryl-substituted DHIQs as aromatic imine analogues for imine-directed C–H functionalization, a 1:1 reaction between **5aa** and a Cp*-ligated Rh(III) complex [Cp*RhCl_2_]_2_ was carried out at room temperature with sodium acetate as an additive (Fig. 5b). Although this transformation occurred with incomplete conversion, we were able to isolate a Rh(III) product with a cyclometalated DHIQ ligand (**11**) and characterized its solid-state structure by single-crystal X-ray diffraction. This {Cp*RhCl[η^2^-(C,N)-DHIQ]} complex resulted from regioselective C–H activation of **5aa** at the *ortho*-position of 1-phenyl instead of 3-phenyl substituent. Notably, a preliminary test reaction between the oxidative [4+2] annulation product **4a** and [Cp*RhCl_2_]_2_ under similar conditions failed to generate cyclometalation products. Such reactivity difference between **5aa** and **4a** may result from the more rigid structure of isoquinoline than DHIQ, which led to more significant steric crowding by phenyl substituents and 1- and 3-positions that inhibits imine-directed aromatic C–H activation.

The Rh(III)-mediated regioselective C–H activation with **5aa** was further exploited in two catalytic transformations following reported procedures by Li^65^ and Glorius^66^, both using [Cp*RhCl_2_]_2_ as the catalyst precursor (Fig. 5c). A coupling between **5aa** and the organoboron reagent ^*n*^BuBF_3_K gave alkylated DHIQ product **12** in 68% yield^65^. Consistent with the stoichiometric cyclometalation result (Fig. 5b), the *n*-butyl group was attached selectively at an *ortho*-position of the 1-phenyl group. Notably, the *cis*-3,4-diphenyl stereochemistry of the DHIQ backbone was retained in both compounds **11** and **12**. By contrast, **5aa** underwent C–H bromination with the NBS reagent in same regioselectivity but dehydrogenated under the reaction conditions to give brominated isoquinoline product **13** in 74% yield^66^. Although it is difficult to rationalize the observation of such dehydrogenation under non-oxidation conditions without a systematic investigation, this result resonates with the recent report by the Wang group on Mn-catalysed dehydrogenative [4+2] imine/alkyne annulation for isoquinoline synthesis^30^. In another preliminary reactivity evaluation, we found that the isoquinoline compound **4a** failed to undergo C–H bromination under similar reaction conditions to form **13**, presumably due to the steric hindrance against cyclometalation as previously discussed. Thus, DHIQ products from current study could also be explored as isoquinoline precursors by tandem functionalization-dehydrogenation strategy.

In summary, we have developed a single catalyst-based approach towards atom-efficient N-heterocycle construction by tandem C–H activation, alkyne coupling, and intramolecular alkene hydroamination. The mechanism-based catalyst development led to the combination of a cationic Rh(I) catalyst precursor and a bis(phosphine) ligand DPEphos, which promotes a redox-neutral [4+2] annulation between N–H aromatic ketimines and internal alkynes to form *cis*-3,4-disubstituted 3,4-dihydroisoquinolines (DHIQs) in high chemo-, regio- and stereoselectivity. With a proposed ligand-enabled intramolecular alkene hydroamination to introduce C3- and C4-chirality, this method can be potentially developed into an enantioselective version for asymmetric synthesis of poly-substituted chiral DHIQ building blocks towards highly valuable THIQ structures. The current strategy of combining metal-catalysed C–H functionalization and alkene hydrofunctionalization represents a unified synthetic approach towards various 6-membered benzoheterocycles by redox-neutral [4+2] annulation between aromatic compounds and alkynes.

## Methods

### General procedure for redox-neutral [4+2] imine/alkyne annulations

Into a 4 ml scintillation vial equipped with a magnetic stir bar was placed [Rh(cod)_2_]BF_4_ (**6**, 5.6 mg, 0.014 mmol, 0.050 equiv.), DPEphos (**7**, 8.9 mg, 0.017 mmol, 0.060 equiv.), and 1.0 ml of toluene. Next, N–H ketimine **1** (0.28 mmol, 1.0 equiv.) and internal alkyne **2** (0.31 mmol, 1.1 equiv.) were added into the vial for the synthesis of products **5aa**–**5aq** (demonstration of alkyne substrate scope). For the synthesis of products **5ba**–**5oa** (demonstration of imine substrate scope), 0.28 mmol alkyne **2** (1.0 equiv.) and 0.31 mmol of imine **1** (1.1 equiv.) were added instead. The vial was sealed with a silicone-lined screw-cap, transferred out of the glovebox, and stirred at 100 °C for 24 h. After the reaction mixture was cooled to room temperature, all volatile materials were removed under reduced pressure. Further purification was achieved by flash-column chromatography using neutral alumina. Yields of the isolated products are based on the average of two runs under identical conditions. The exclusive *cis*-diastereoselectivity for products was determined by GC and ^1^H NMR analysis of the unpurified reaction mixture. See Supplementary Figs 1–87 and Supplementary Methods for full experimental details and analytical data for characterization of new compounds.

## Additional information

**Accession codes:** The X-ray crystallographic coordinates for structures reported in this study have been deposited at the Cambridge Crystallographic Data Centre (CCDC), under deposition numbers 1424375-1424378 (compounds **5aa**, **9a**, **5na** and **11**) and 1442483 (compound **5an**). These data can be obtained free of charge from The Cambridge Crystallographic Data Centre via www.ccdc.cam.ac.uk/data_request/cif.

**How to cite this article:** Manan, R. S. *et al.* Merging rhodium-catalysed C–H activation and hydroamination in a highly selective [4+2] imine/alkyne annulation. *Nat. Commun.* 7:11506 doi: 10.1038/ncomms11506 (2016).

## Supplementary Material

Supplementary Information Supplementary Figures 1-90, Supplementary Table 1, Supplementary Discussion, Supplementary Methods and Supplementary References

## Figures and Tables

**Figure 1:**
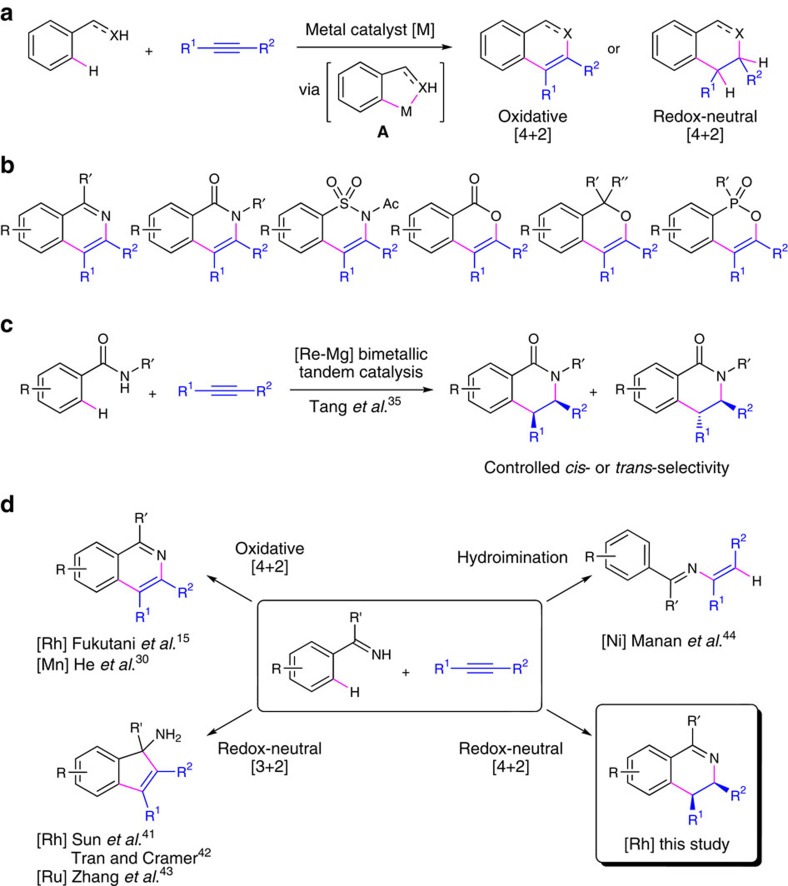
Transition metal-catalyzed [4+2] annulations and related strategies. (**a**) Catalytic [4+2] annulations between aromatic compounds and alkynes via cyclometalated intermediates (**A**); XH=H-substituted σ-donating functional group to direct aromatic C–H activation at the *ortho*-position. (**b**) Reported benzoheterocycle products from oxidative [4+2] annulations with NH or OH directing groups. (**c**) A Re–Mg bimetallic catalyst system for redox-neutral [4+2] annulation between benzamides and alkynes. (**d**) Divergent catalytic couplings between N–H aromatic ketimines and alkynes.

**Figure 3 f3:**
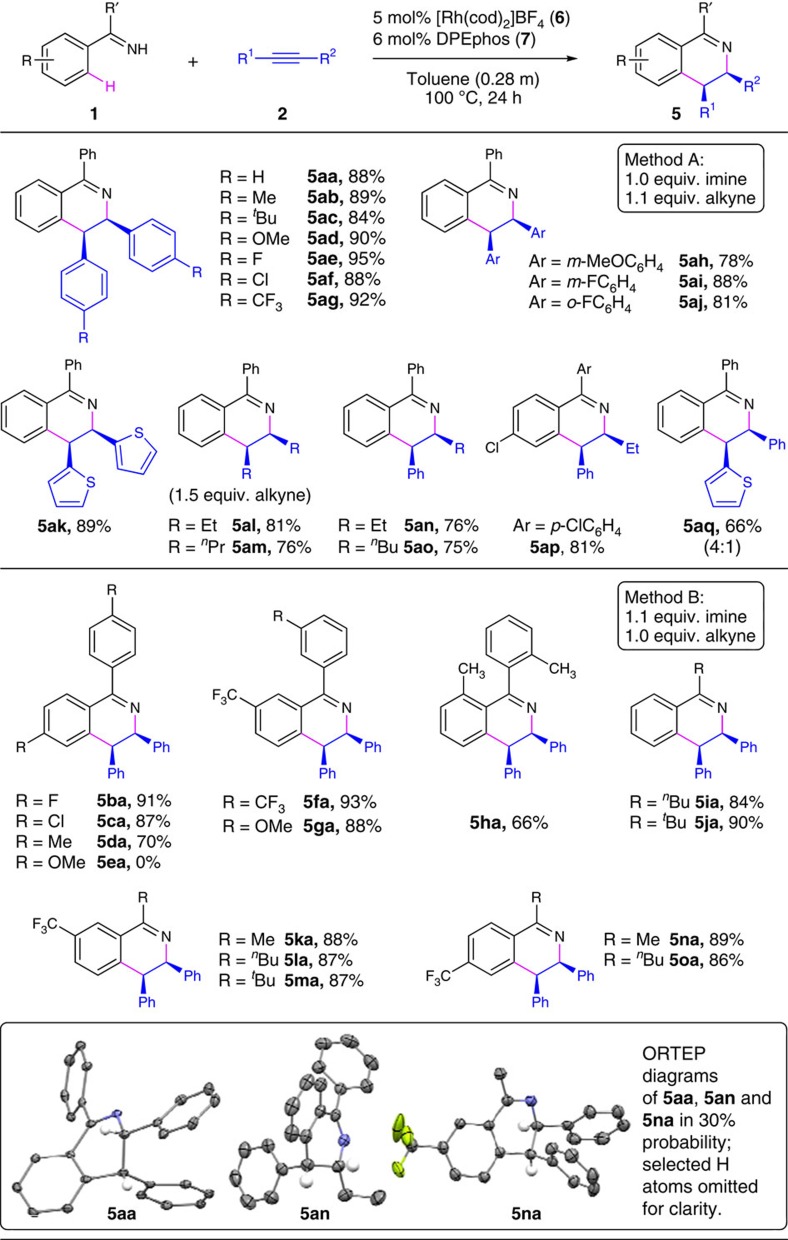
Scope of Rh-catalyzed redox-neutral [4+2] imine/alkyne annulation. General reaction conditions: **1** (0.28 mmol for Method A, 0.31 mmol for Method B), **2** (0.31 mmol for Method A, 0.28 mmol for Method B); [Rh(cod)_2_]BF_4_ (**6**, 5.0 mol%), DPEphos (**7**, 6.0 mol%), toluene (1.0 ml), 100 °C, 24 h; averaged yield of isolated products from two runs.

**Table 1:**
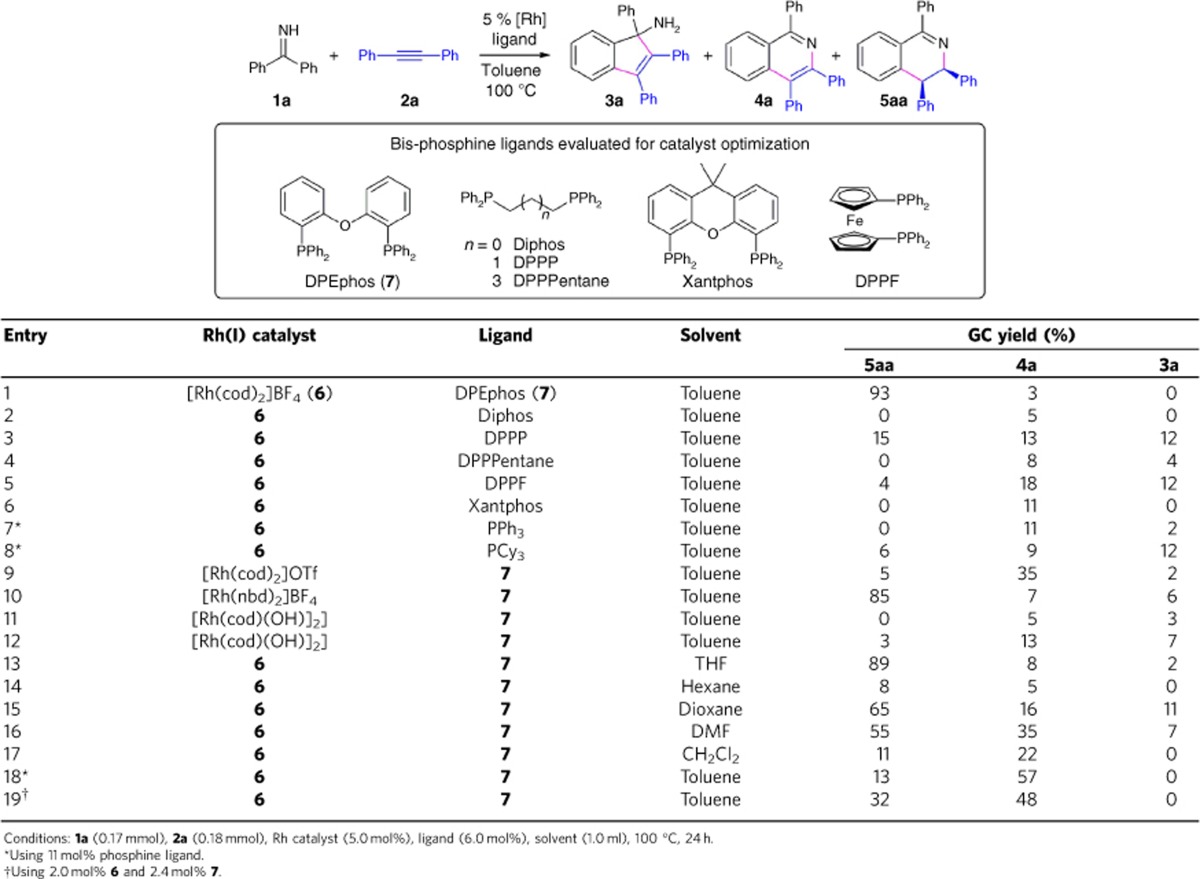
Optimization of conditions for redox-neutral [4+2] imine/alkyne annulation.

## References

[b1] Yu J.-Q., & Shi Z. (eds). C–H Activation Topics in Current Chemistry, Vol. 292 (Springer, 2010). 21500400

[b2] Colby D. A., Bergman R. G. & Ellman J. A. Rhodium-catalyzed C–C bond formation via heteroatom-directed C–H bond activation. Chem. Rev. 110, 624–655 (2010). 19438203 10.1021/cr900005n PMC2820156

[b3] Lyons T. W. & Sanford M. S. Palladium-catalyzed ligand-directed C–H functionalization reactions. Chem. Rev. 110, 1147–1169 (2010). 20078038 10.1021/cr900184e PMC2836499

[b4] Satoh T. & Miura M. Oxidative coupling of aromatic substrates with alkynes and alkenes under rhodium catalysis. Chem. Eur. J 16, 11212–11222 (2010). 20740508 10.1002/chem.201001363

[b5] Mei T.-S., Kou L., Ma S., Engle K. M. & Yu J.-Q. Heterocycle formation via palladium-catalyzed C-H functionalization. Synthesis 44, 1778–1791 (2012). 10.1055/s-0031-1289766 PMC4936280 27397938

[b6] Song G., Wang F. & Li X. C–C, C–O and C–N bond formation via rhodium(iii)-catalyzed oxidative C-H activation. Chem. Soc. Rev. 41, 3651–3678 (2012). 22377832 10.1039/c2cs15281a

[b7] Ackermann L. Carboxylate-assisted ruthenium-catalyzed alkyne annulations by C-H/Het-H bond functionalizations. Acc. Chem. Res. 47, 281–295 (2014). 23379589 10.1021/ar3002798

[b8] He R., Huang Z.-T., Zheng Q.-Y. & Wang C. Isoquinoline skeleton synthesis via chelation-assisted C-H activation. Tetrahedron Lett. 55, 5705–5713 (2014).

[b9] Albrecht M. Cyclometalation using *d*-block transition metals: fundamental aspects and recent trends. Chem. Rev. 110, 576–623 (2010). 20017477 10.1021/cr900279a

[b10] Li L., Brennessel W. W. & Jones W. D. An efficient low-temperature route to polycyclic isoquinoline salt synthesis via C–H activation with [Cp*MCl_2_]_2_ (M=Rh, Ir). J. Am. Chem. Soc. 130, 12414–12419 (2008). 18714995 10.1021/ja802415h

[b11] Li L., Brennessel W. W. & Jones W. D. C–H activation of phenyl imines and 2-phenylpyridines with [Cp*MCl_2_]_2_ (M=Ir, Rh): regioselectivity, kinetics, and mechanism. Organometallics 28, 3492–3500 (2009).

[b12] Lim S.-G., Lee J. H., Moon C. W., Hong J.-B. & Jun C.-H. Rh(I)-catalyzed direct *ortho*-alkenylation of aromatic ketimines with alkynes and its application to the synthesis of isoquinoline derivatives. Org. Lett. 5, 2759–2761 (2003). 12868908 10.1021/ol035083d

[b13] Ueura K., Satoh T. & Miura M. An efficient waste-free oxidative coupling via regioselective C–H bond cleavage: Rh/Cu-catalyzed reaction of benzoic acids with alkynes and acrylates under air. Org. Lett. 9, 1407–1409 (2007). 17346060 10.1021/ol070406h

[b14] Umeda N., Tsurugi H., Satoh T. & Miura M. Fluorescent naphthyl- and anthrylazoles from the catalytic coupling of phenylazoles with internal alkynes through the cleavage of multiple C–H bonds. Angew. Chem. Int. Ed. 47, 4019–4022 (2008). 10.1002/anie.200800924 18418815

[b15] Fukutani T., Umeda N., Hirano K., Satoh T. & Miura M. Rhodium-catalyzed oxidative coupling of aromatic imines with internal alkynes via regioselective C-H bond cleavage. Chem. Commun. 2009, 5141–5143 (2009). 10.1039/b910198e 20448973

[b16] Guimond N. & Fagnou K. Isoquinoline synthesis via rhodium-catalyzed oxidative cross-coupling/cyclization of aryl aldimines and alkynes. J. Am. Chem. Soc. 131, 12050–12051 (2009). 19705909 10.1021/ja904380q

[b17] Parthasarathy K. & Cheng C.-H. Easy access to isoquinolines and tetrahydroquinolines from ketoximes and alkynes via rhodium-catalyzed C–H bond activation. J. Org. Chem. 74, 9359–9364 (2009). 19894732 10.1021/jo902084j

[b18] Guimond N., Gouliaras C. & Fagnou K. Rhodium(III)-catalyzed isoquinolone synthesis: the N–O bond as a handle for C–N bond formation and catalyst turnover. J. Am. Chem. Soc. 132, 6908–6909 (2010). 20433170 10.1021/ja102571b

[b19] Mochida S., Umeda N., Hirano K., Satoh T. & Miura M. Rhodium-catalyzed oxidative coupling/cyclization of benzamides with alkynes via C-H bond cleavage. Chem. Lett. 39, 744–746 (2010).

[b20] Song G., Chen D., Pan C.-L., Crabtree R. H. & Li X. Rh-catalyzed oxidative coupling between primary and secondary benzamides and alkynes: synthesis of polycyclic amides. J. Org. Chem. 75, 7487–7490 (2010). 20923219 10.1021/jo101596d

[b21] Hyster T. K. & Rovis T. Pyridine synthesis from oximes and alkynes via rhodium(III) catalysis: Cp* and Cp^t^ provide complementary selectivity. Chem. Commun. 47, 11846–11848 (2011). 10.1039/c1cc15248c PMC3430144 21986995

[b22] Morimoto K., Hirano K., Satoh T. & Miura M. Synthesis of isochromene and related derivatives by rhodium-catalyzed oxidative coupling of benzyl and allyl alcohols with alkynes. J. Org. Chem. 76, 9548–9551 (2011). 21988500 10.1021/jo201923d

[b23] Too P. C., Chua S. H., Wong S. H. & Chiba S. Synthesis of azaheterocycles from aryl ketone O-acetyl oximes and internal alkynes by Cu-Rh bimetallic relay catalysts. J. Org. Chem. 76, 6159–6168 (2011). 21688786 10.1021/jo200897q

[b24] Ackermann L., Pospech J., Graczyk K. & Rauch K. Versatile synthesis of isocoumarins and a-pyrones by ruthenium-catalyzed oxidative C–H/O–H bond cleavages. Org. Lett. 14, 930–933 (2012). 22273364 10.1021/ol2034614

[b25] Chinnagolla R. K. & Jeganmohan M. Regioselective synthesis of isocoumarins by ruthenium-catalyzed aerobic oxidative cyclization of aromatic acids with alkynes. Chem. Commun. 48, 2030–2032 (2012). 10.1039/c2cc16916a 22227858

[b26] Zhong H., Yang D., Wang S. & Huang J. Pd-catalysed synthesis of isoquinolinones and analogues via C–H and N–H bonds double activation. Chem. Commun. 48, 3236–3238 (2012). 10.1039/c2cc17859a 22334061

[b27] Pham M. V., Ye B. & Cramer N. Access to sultams by rhodium(III)-catalyzed directed C-H activation. Angew. Chem. Int. Ed. 51, 10610–10614 (2012). 10.1002/anie.201206191 23002028

[b28] Unoh Y. *et al.* Rhodium(III)-catalyzed oxidative coupling through C–H bond cleavage directed by phosphinoxy groups. Org. Lett. 15, 3258–3261 (2013). 23772867 10.1021/ol4012794

[b29] Villuendas P. & Urriolabeitia E. P. Primary amines as directing groups in the Ru-catalyzed synthesis of isoquinolines, benzoisoquinolines, and thienopyridines. J. Org. Chem. 78, 5254–5263 (2013). 23650873 10.1021/jo400344m

[b30] He R., Huang Z.-T., Zheng Q.-Y. & Wang C. Manganese-catalyzed dehydrogenative [4+2] annulation of N–H imines and alkynes by C–H/N–H activation. Angew. Chem. Int. Ed. 53, 4950–4953 (2014). 10.1002/anie.201402575 24700597

[b31] Li J. & Ackermann L. Ruthenium-catalyzed oxidative alkyne annulation by C-H activation on ketimines. Tetrahedron 70, 3342–3348 (2014).

[b32] Nakanowatari S. & Ackermann L. Ruthenium(II)-catalyzed synthesis of isochromenes by C-H activation with weakly coordinating aliphatic hydroxyl groups. Chemistry 20, 5409–5413 (2014). 24677395 10.1002/chem.201400161

[b33] Neufeldt S. R., Jimenez-Oses G., Huckins J. R., Thiel O. R. & Houk K. N. Pyridine N-oxide vs pyridine substrates for Rh(III)-catalyzed oxidative C–H bond functionalization. J. Am. Chem. Soc. 137, 9843–9854 (2015). 26197041 10.1021/jacs.5b03535

[b34] Warratz S. *et al.* Ruthenium(II)-catalyzed C–H activation/alkyne annulation by weak coordination with O_2_ as the sole oxidant. Angew. Chem. Int. Ed. 54, 5513–5517 (2015). 10.1002/anie.201500600 25737001

[b35] Tang Q., Xia D., Zhang Q., Sun X.-Q. & Wang C. Re/Mg bimetallic tandem catalysis for [4+2] annulation of benzamides and alkynes via C–H/N–H functionalization. J. Am. Chem. Soc. 135, 4628–4631 (2013). 23469938 10.1021/ja400020e

[b36] Mueller T. E., Hultzsch K. C., Yus M., Foubelo F. & Tada M. Hydroamination: direct addition of amines to alkenes and alkynes. Chem. Rev. 108, 3795–3892 (2008). 18729420 10.1021/cr0306788

[b37] Huang L., Arndt M., Goossen K., Heydt H. & Goossen L. J. Late transition metal-catalyzed hydroamination and hydroamidation. Chem. Rev. 115, 2596–2697 (2015). 25721762 10.1021/cr300389u

[b38] Yi C. S. & Yun S. Y. Scope and mechanistic study of the ruthenium-catalyzed ortho-C-H bond activation and cyclization reactions of arylamines with terminal alkynes. J. Am. Chem. Soc. 127, 17000–17006 (2005). 16316246 10.1021/ja055608s PMC2585980

[b39] Lium X.-Y., Ding P., Huang J.-S. & Che C.-M. Synthesis of substituted 1,2-dihydroquinolines and quinolines from aromatic amines and alkynes by gold(I)-catalyzed tandem hydroamination–hydroarylation under microwave-assisted conditions. Org. Lett. 9, 2645–2648 (2007). 17564458 10.1021/ol070814l

[b40] Zeng X., Frey G. D., Kinjo R., Donnadieu B. & Bertrand G. Synthesis of a simplified version of stable bulky and rigid cyclic (Alkyl)(amino)carbenes, and catalytic activity of the ensuing gold(I) complex in the three-component preparation of 1,2-dihydroquinoline derivatives. J. Am. Chem. Soc. 131, 8690–8696 (2009). 19456108 10.1021/ja902051m PMC2724870

[b41] Sun Z.-M., Chen S.-P. & Zhao P. Tertiary carbinamine synthesis by rhodium-catalyzed [3+2] annulation of N-unsubstituted aromatic ketimines and alkynes. Chem. Eur. J 16, 2619–2627 (2010). 20077543 10.1002/chem.200902814

[b42] Tran D. N. & Cramer N. Enantioselective rhodium(I)-catalyzed [3+2] annulations of aromatic ketimines induced by directed C–H activations. Angew. Chem. Int. Ed. 50, 11098–11102 (2011). 10.1002/anie.201105766 21976453

[b43] Zhang J., Ugrinov A. & Zhao P. Ruthenium(II)/N-heterocyclic carbene catalyzed [3+2] carbocyclization with aromatic N–H ketimines and internal alkynes. Angew. Chem. Int. Ed. 52, 6681–6684 (2013). 10.1002/anie.201209031 PMC4310470 23696055

[b44] Manan R. S., Kilaru P. & Zhao P. Nickel-catalyzed hydroimination of alkynes. J. Am. Chem. Soc. 137, 6136–6139 (2015). 25923248 10.1021/jacs.5b02272

[b45] Scott J. D. & Williams R. M. Chemistry and biology of the tetrahydroisoquinoline antitumor antibiotics. Chem. Rev. 102, 1669–1730 (2002). 11996547 10.1021/cr010212u

[b46] Kwan E. E. & Huang S. G. Structural elucidation with NMR spectroscopy: practical strategies for organic chemists. Eur. J. Biochem. 2671–2688 (2008).

[b47] Murai S. *et al.* Efficient catalytic addition of aromatic carbon-hydrogen bonds to olefins. Nature 366, 529–531 (1993).

[b48] Kakiuchi F., Yamamoto Y., Chatani N. & Murai S. Catalytic addition of aromatic C-H bonds to acetylenes. Chem. Lett. 24, 681–682 (1995).

[b49] Colby D. A., Bergman R. G. & Ellman J. A. Synthesis of dihydropyridines and pyridines from imines and alkynes via C–H activation. J. Am. Chem. Soc. 130, 3645–3651 (2008). 18302381 10.1021/ja7104784 PMC3057408

[b50] Duttwyler S., Lu C., Rheingold A. L., Bergman R. G. & Ellman J. A. Highly diastereoselective synthesis of tetrahydropyridines by a C–H activation-cyclization-reduction cascade. J. Am. Chem. Soc. 134, 4064–4067 (2012). 22356093 10.1021/ja2119833 PMC3319108

[b51] Duttwyler S. *et al.* Proton donor acidity controls selectivity in nonaromatic nitrogen heterocycle synthesis. Science 339, 678–682 (2013). 23393259 10.1126/science.1230704 PMC3809088

[b52] Utsunomiya M., Kuwano R., Kawatsura M. & Hartwig J. F. Rhodium-catalyzed anti-Markovnikov hydroamination of vinylarenes. J. Am. Chem. Soc. 125, 5608–5609 (2003). 12733880 10.1021/ja0293608

[b53] Takemiya A. & Hartwig J. F. Rhodium-catalyzed intramolecular, anti-Markovnikov hydroamination. Synthesis of 3-arylpiperidines. J. Am. Chem. Soc. 128, 6042–6043 (2006). 16669666 10.1021/ja058299e

[b54] Liu Z. & Hartwig J. F. Mild, rhodium-catalyzed intramolecular hydroamination of unactivated terminal and internal alkenes with primary and secondary amines. J. Am. Chem. Soc. 130, 1570–1571 (2008). 18183986 10.1021/ja710126x PMC2814333

[b55] Liu Z., Yamamichi H., Madrahimov S. T. & Hartwig J. F. Rhodium phosphine-π-arene intermediates in the hydroamination of alkenes. J. Am. Chem. Soc. 133, 2772–2782 (2011). 21309512 10.1021/ja1057949 PMC3075068

[b56] van Leeuwen P. W, Kamer P. C. J., Reek J. N. H. & Dierkes P. Ligand bite angle effects in metal-catalyzed C–C bond formation. Chem. Rev. 100, 2741–2769 (2000). 11749304 10.1021/cr9902704

[b57] Zhang G., Yang L., Wang Y., Xie Y. & Huang H. An efficient Rh/O_2_ catalytic system for oxidative C–H activation/annulation: evidence for Rh(I) to Rh(III) oxidation by molecular oxygen. J. Am. Chem. Soc. 135, 8850–8853 (2013). 23742052 10.1021/ja404414q

[b58] Kitamura T. Transition-metal-catalyzed hydroarylation reactions of alkynes through direct functionalization of C–H bonds. A convenient tool for organic synthesis. Eur. J. Org. Chem. 2009, 1111–1125 (2009).

[b59] Ma S. & Gu Z. 1,4-migration of rhodium and palladium in catalytic organometallic reactions. Angew. Chem. Int. Ed. 44, 7512–7517 (2005). 10.1002/anie.200501298 16273558

[b60] Pahadi N. K., Paley M., Jana R., Waetzig S. R. & Tunge J. A. Formation of N-alkylpyrroles via intermolecular redox amination. J. Am. Chem. Soc. 131, 16626–16627 (2009). 19886656 10.1021/ja907357g PMC2878740

[b61] Deb I., Das D. & Seidel D. Redox isomerization via azomethine ylide intermediates: N-alkyl indoles from indolines and aldehydes. Org. Lett. 13, 812–815 (2010). 21247142 10.1021/ol1031359

[b62] Stockigt J., Antonchick A. P., Wu F.-R. & Waldmann H. The pictet-spengler reaction in nature and in organic chemistry. Angew. Chem. Int. Ed. 50, 8538–8564 (2011). 10.1002/anie.201008071 21830283

[b63] Zhao D. & Glorius F. Enantioselective hydrogenation of isoquinolines. Angew. Chem. Int. Ed. 52, 9616–9618 (2013). 10.1002/anie.201304756 23881748

[b64] Wang D.-S., Chen Q.-A., Lu S.-M. & Zhou Y.-G. Asymmetric hydrogenation of heteroarenes and arenes. Chem. Rev. 112, 2557–2590 (2012). 22098109 10.1021/cr200328h

[b65] Wang H., Yu S., Qi Z. & Li X. Rh(III)-catalyzed C-H alkylation of arenes using alkylboron reagents. Org. Lett. 17, 2812–2815 (2015). 25972069 10.1021/acs.orglett.5b01232

[b66] Schroeder N., Wencel-Delord J. & Glorius F. High-yielding, versatile, and practical [Rh(III)Cp*]-catalyzed ortho bromination and iodination of arenes. J. Am. Chem. Soc. 134, 8298–8301 (2012). 22548632 10.1021/ja302631j

