# Supplementary material for: Merging rhodium-catalysed C–H activation and hydroamination in a highly selective [4+2] imine/alkyne annulation
Source: Nat Commun. 2016 Jun 20;7:11506. doi: 10.1038/ncomms11506 (PMC4915129; doi:10.1038/ncomms11506)
Supplement: Supplementary Information — Supplementary Figures 1-90, Supplementary Table 1, Supplementary Discussion, Supplementary Methods and Supplementary References [file ncomms11506-s1.pdf]

Supplementary Figure 1: ^{1}H NMR Spectrum of **5aa** (400 MHz, $CDCl_3$)

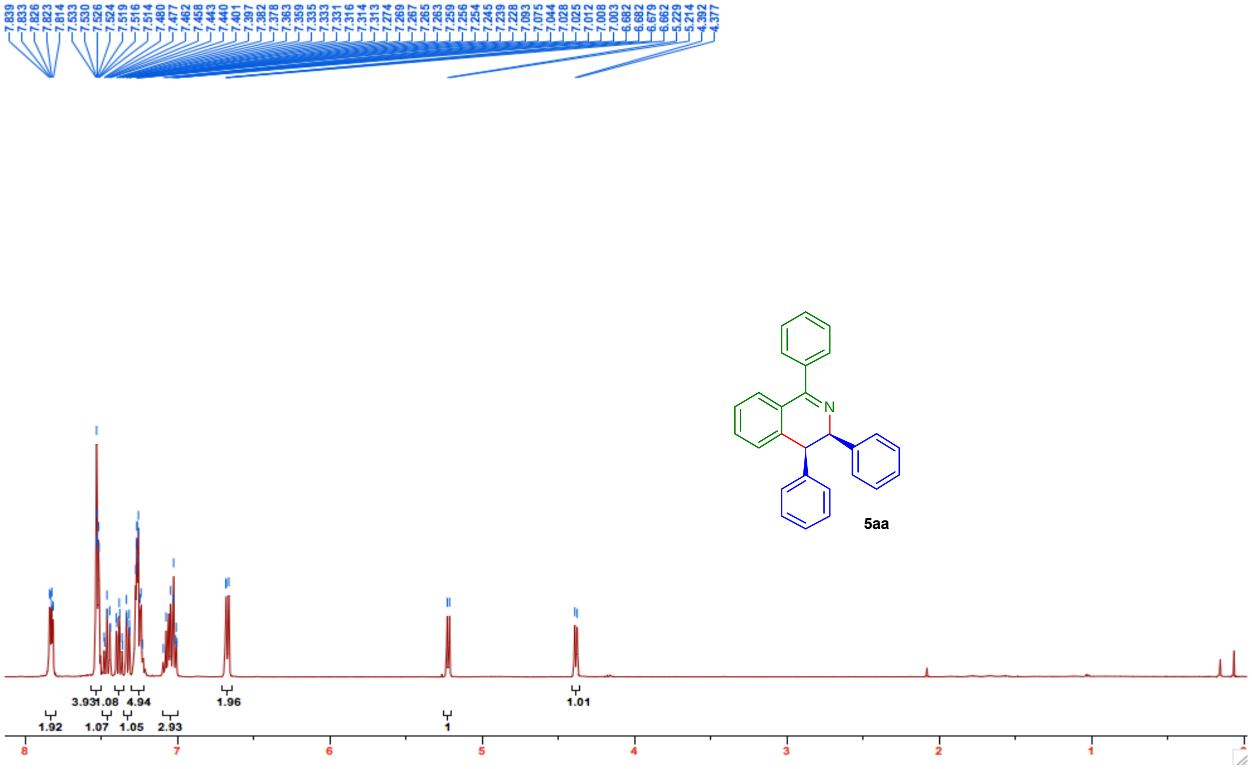

Supplementary Figure 2: ^{13}C NMR Spectrum of **5aa** (100 MHz, $CDCl_3$)

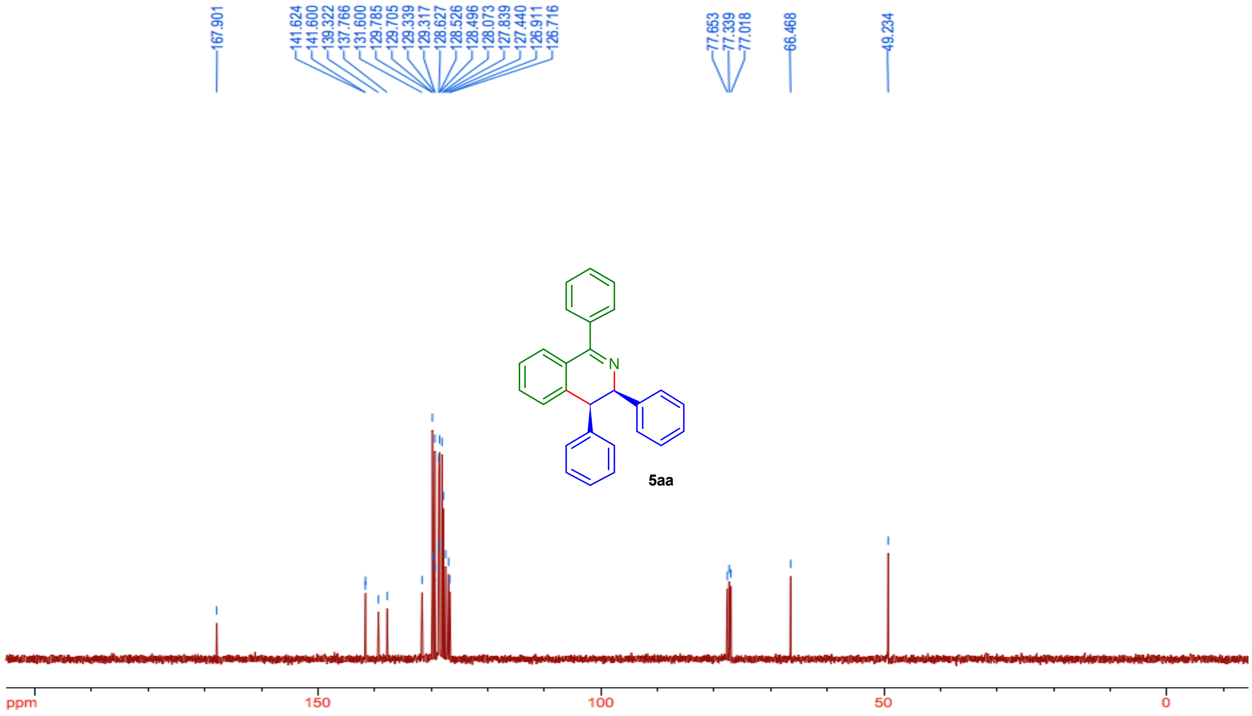

Supplementary Figure 3: ^{1}H NMR Spectrum of **5ab** (400 MHz, $CDCl_3$)

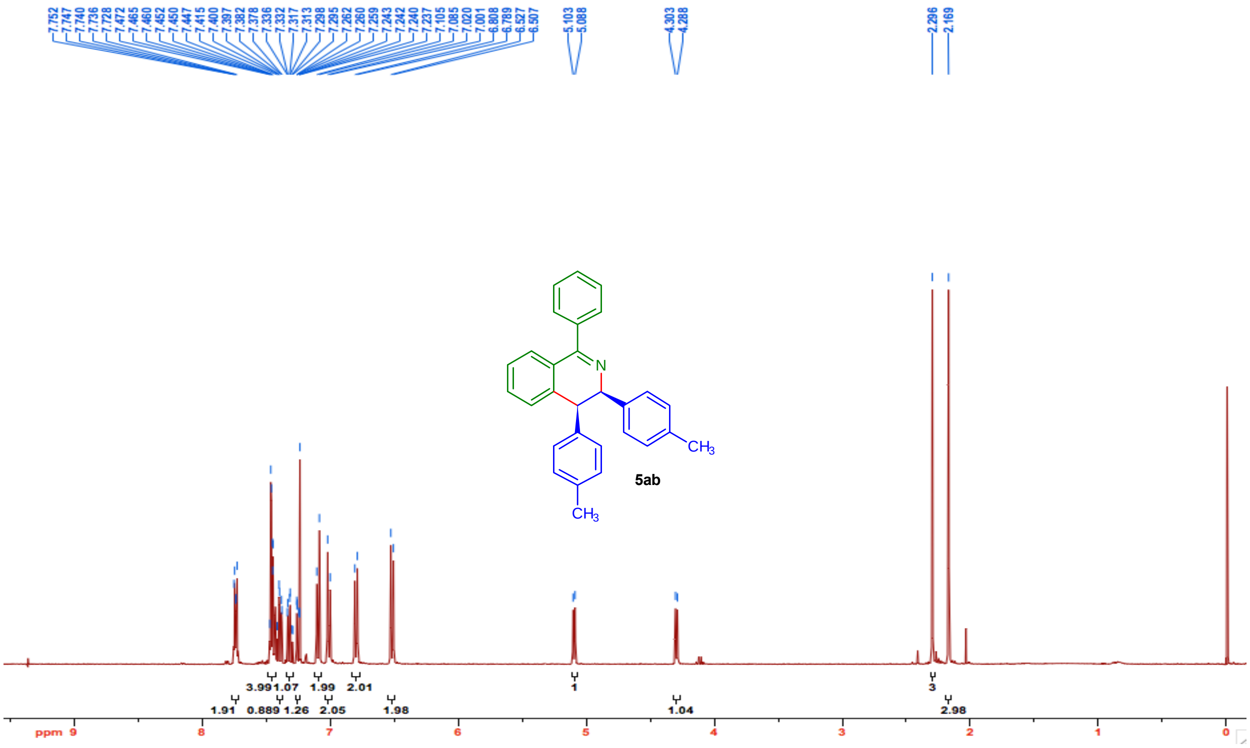

Supplementary Figure 4: ^{13}C NMR Spectrum of **5ab** (100 MHz, $CDCl_3$)

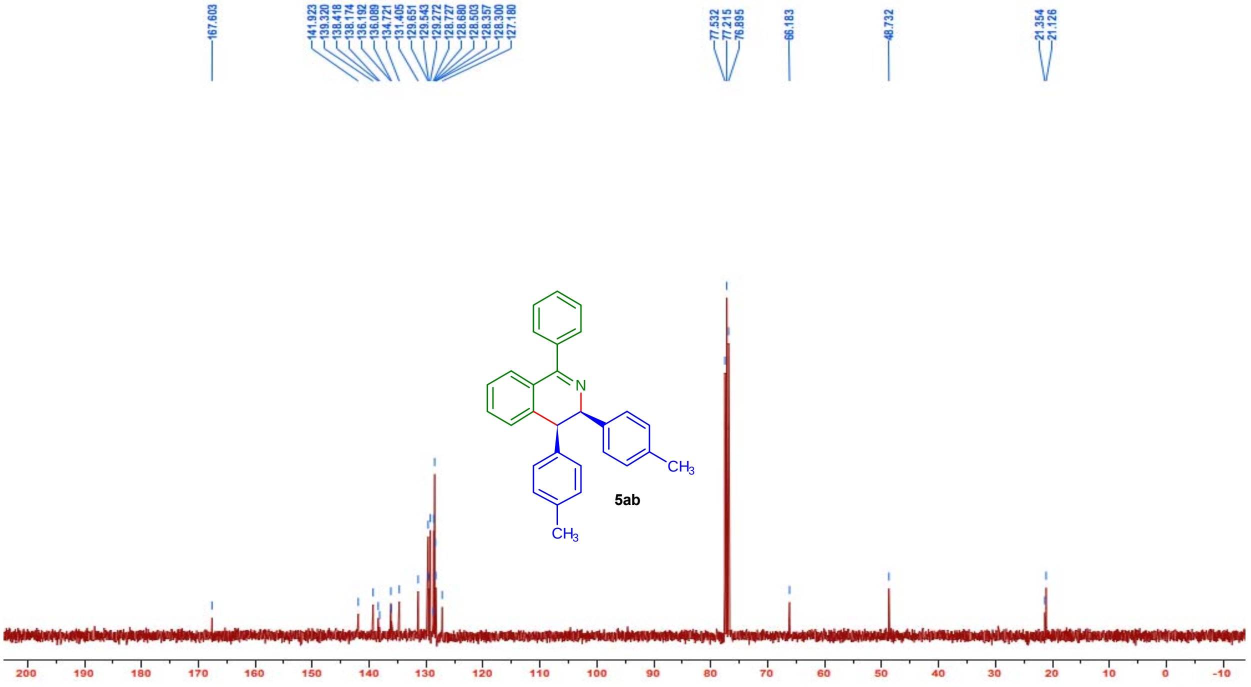

Supplementary Figure 5: ^{1}H NMR Spectrum of **5ac** (400 MHz, $CDCl_3$)

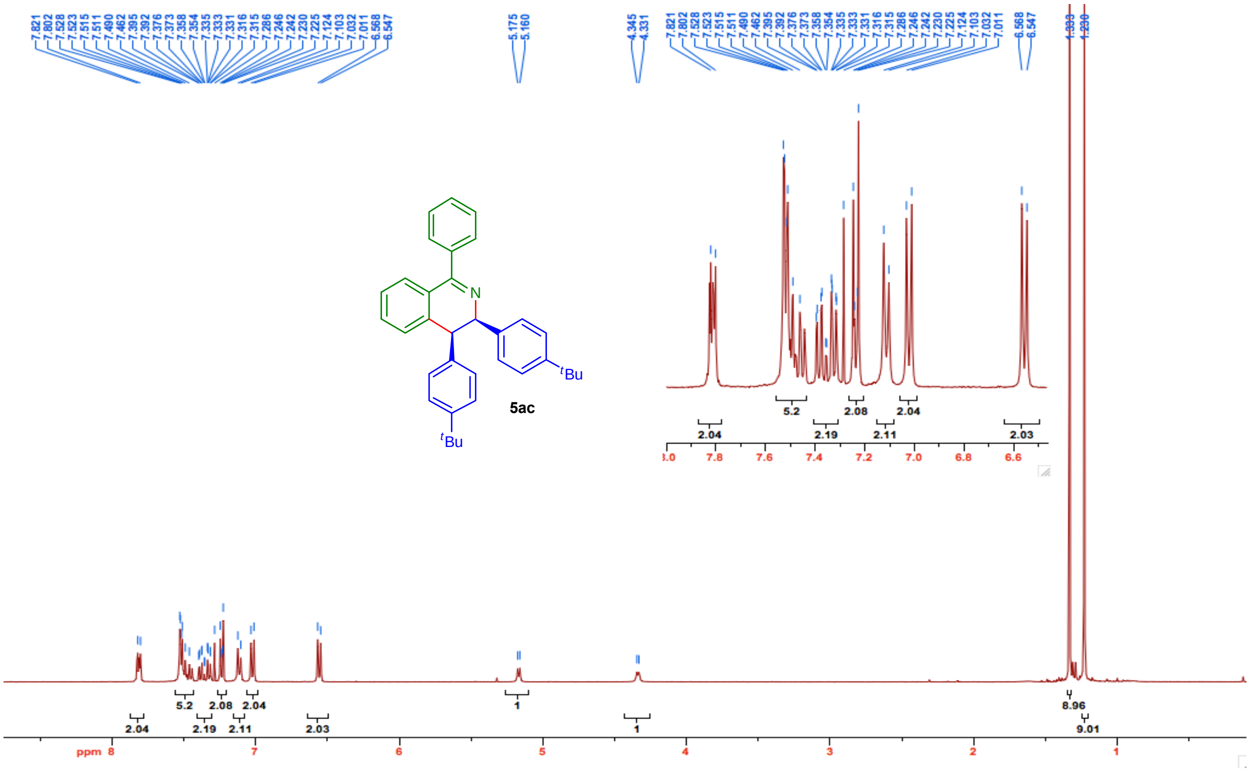

Supplementary Figure 6: ^{13}C NMR Spectrum of **5ac** (100 MHz, $CDCl_3$)

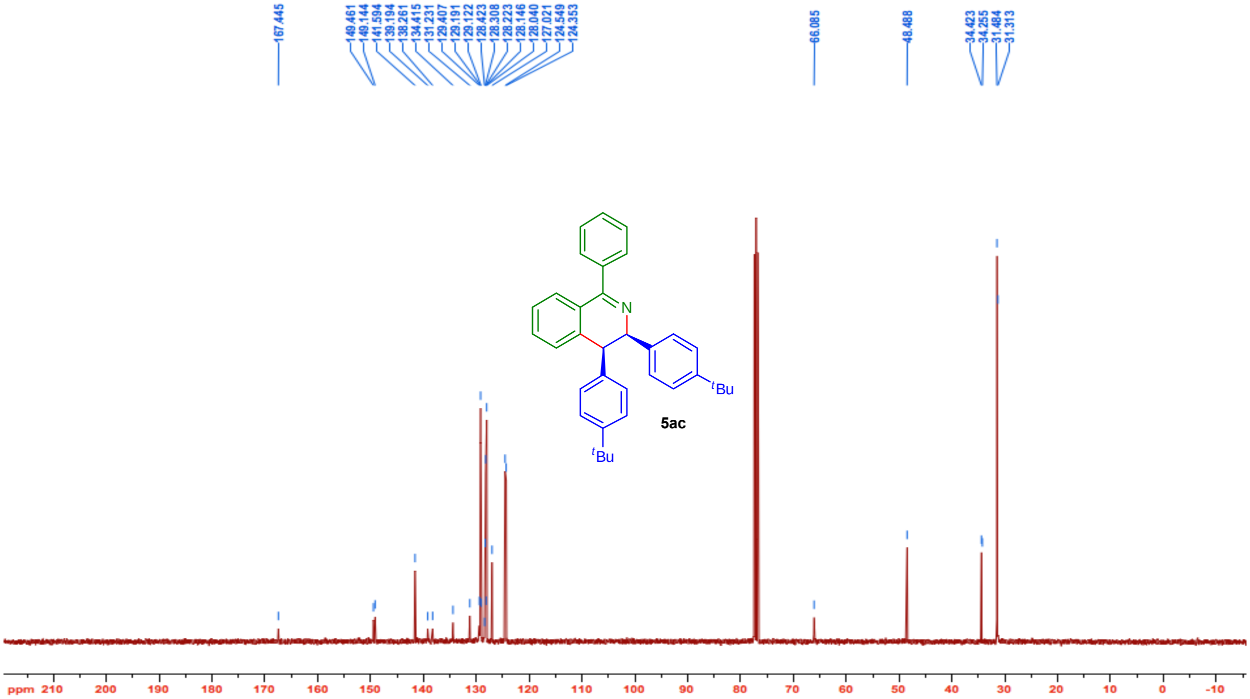

Supplementary Figure 7: ^{1}H NMR Spectrum of **5ad** (400 MHz, $CDCl_3$)

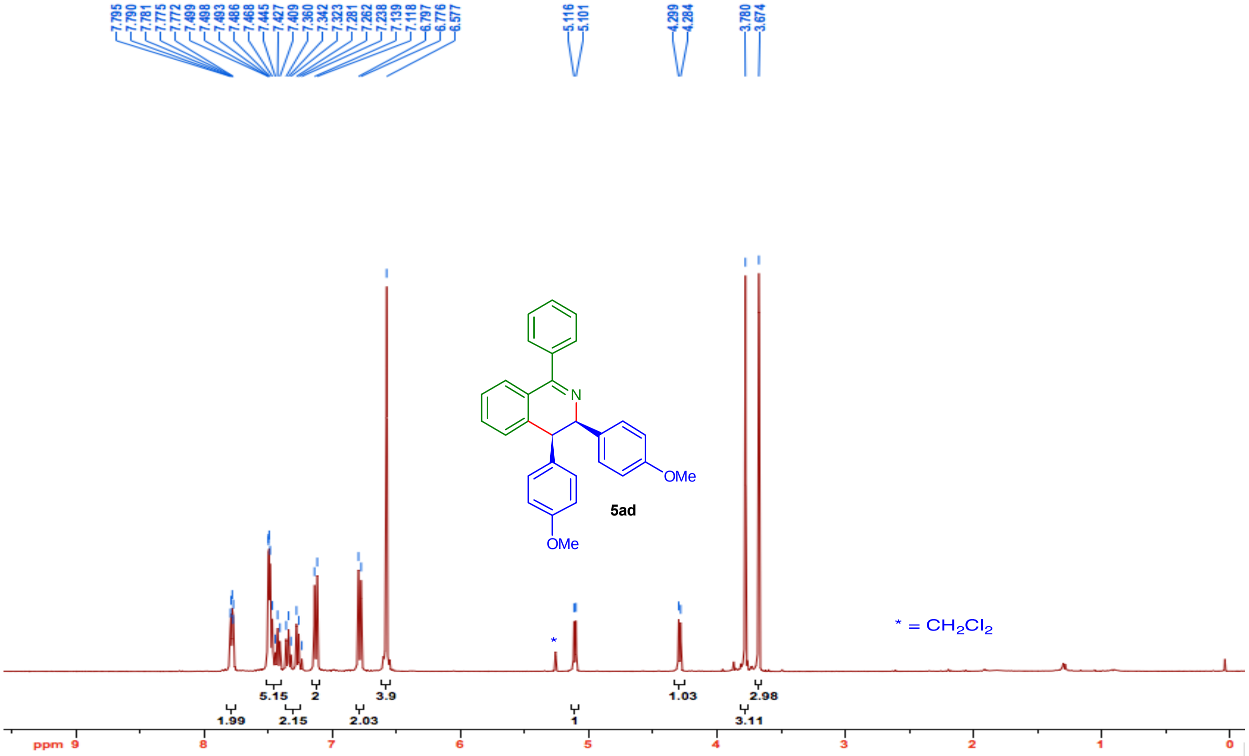

Supplementary Figure 8: ^{13}C NMR Spectrum of **5ad** (100 MHz, $CDCl_3$)

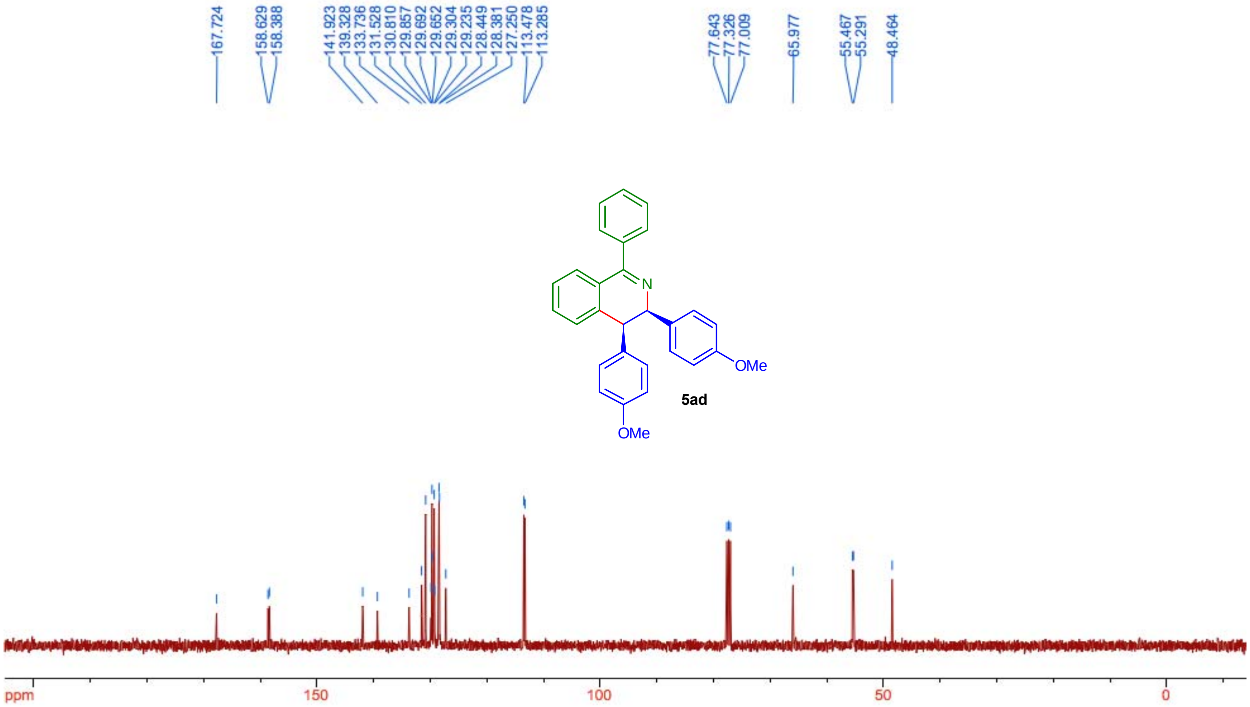

Supplementary Figure 9: ^{1}H NMR Spectrum of **5ae** (400 MHz, $CDCl_3$)

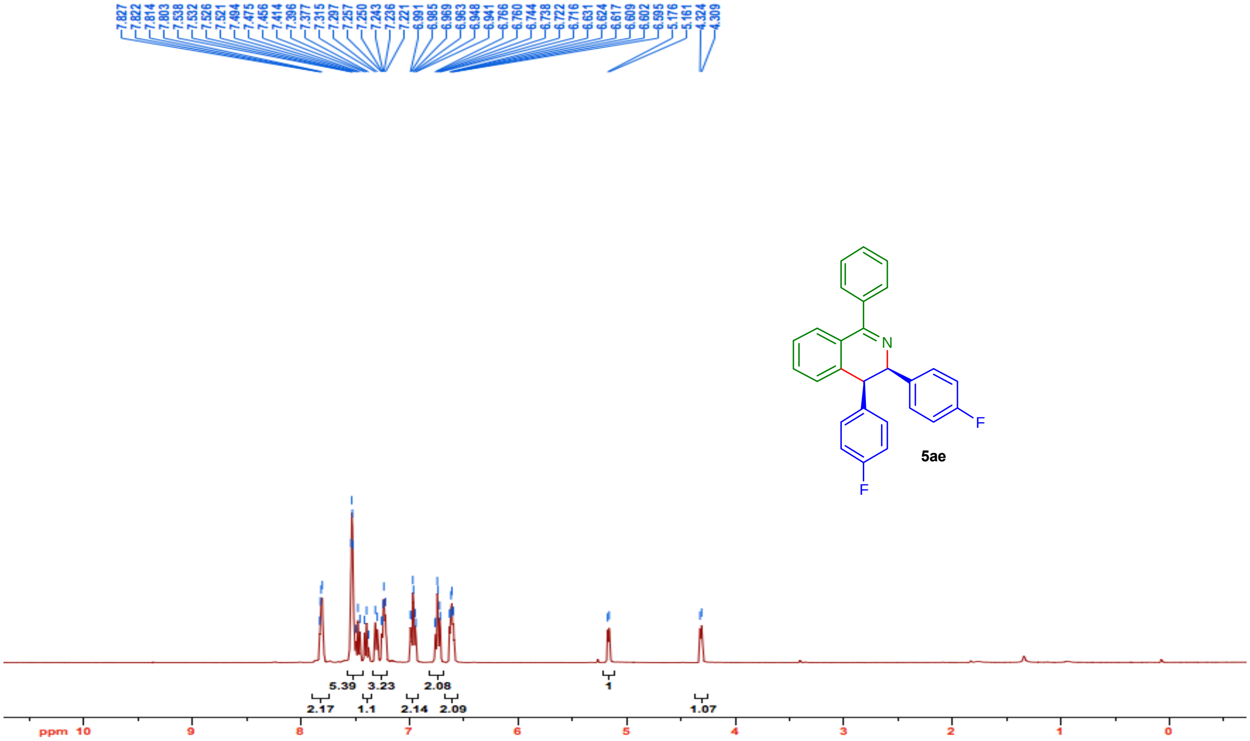

Supplementary Figure 10: ^{13}C NMR Spectrum of **5ae** (100 MHz, $CDCl_3$)

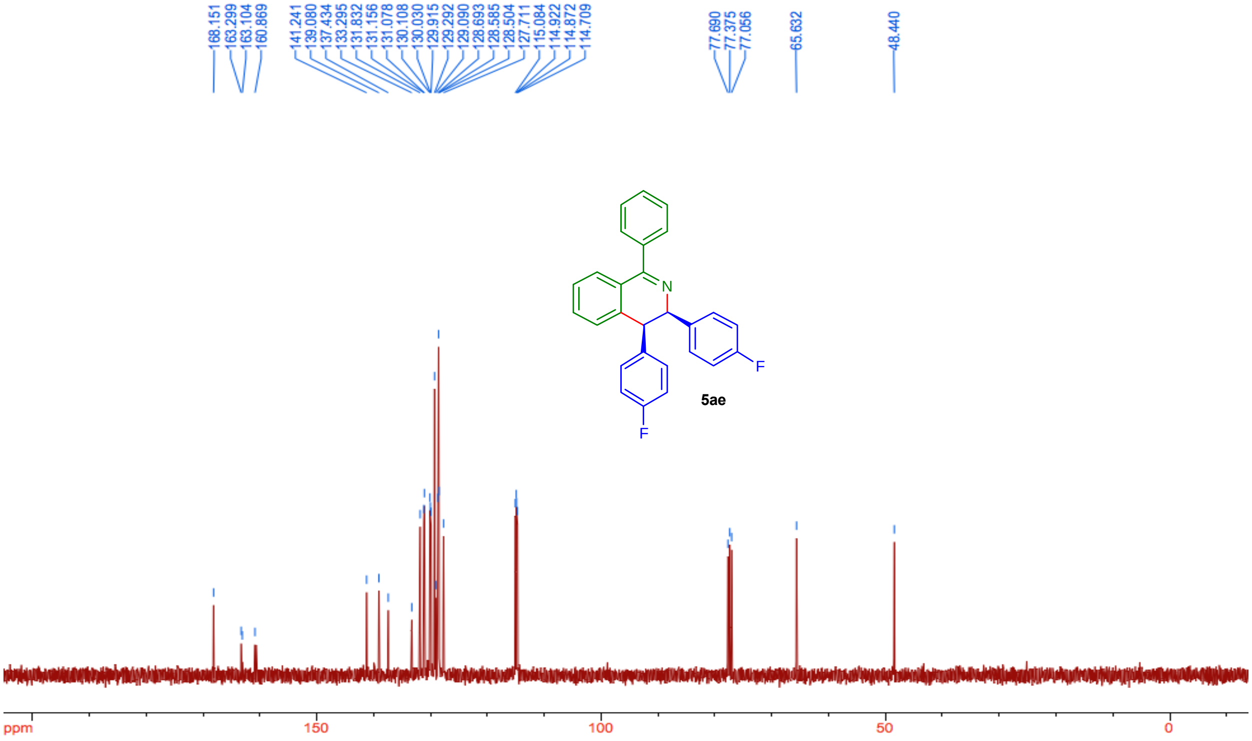

Supplementary Figure 11: ^{19}F NMR Spectrum of **5ae** (376 MHz, $CDCl_3$)

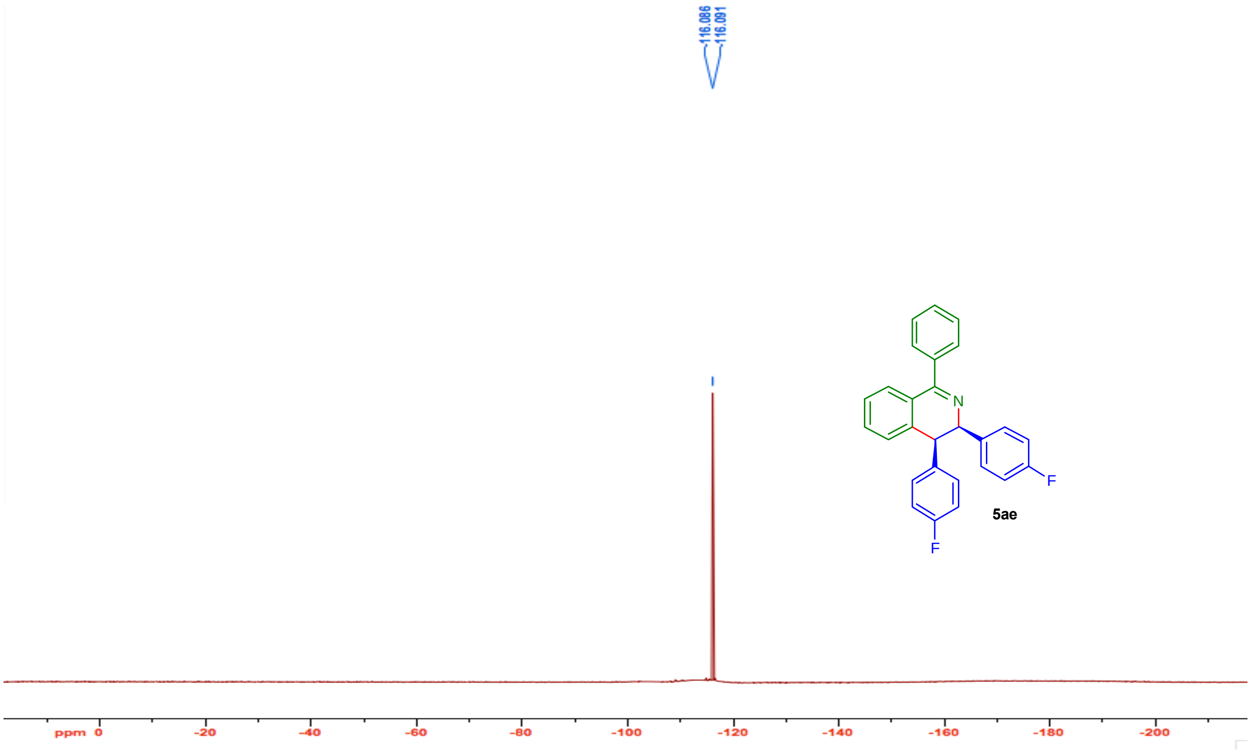

Supplementary Figure 12: ^{1}H NMR Spectrum of **5af** (400 MHz, $CDCl_3$)

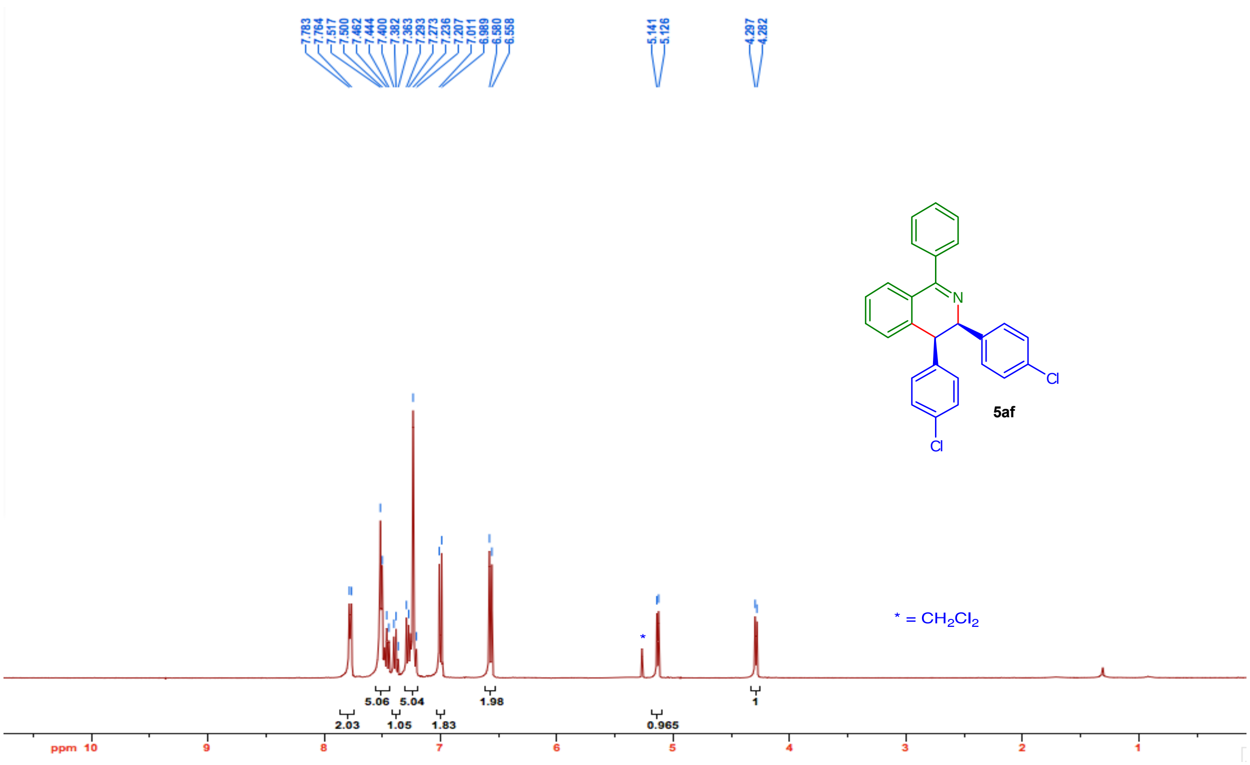

Supplementary Figure 13: ^{13}C NMR Spectrum of **5af** (100 MHz, $CDCl_3$)

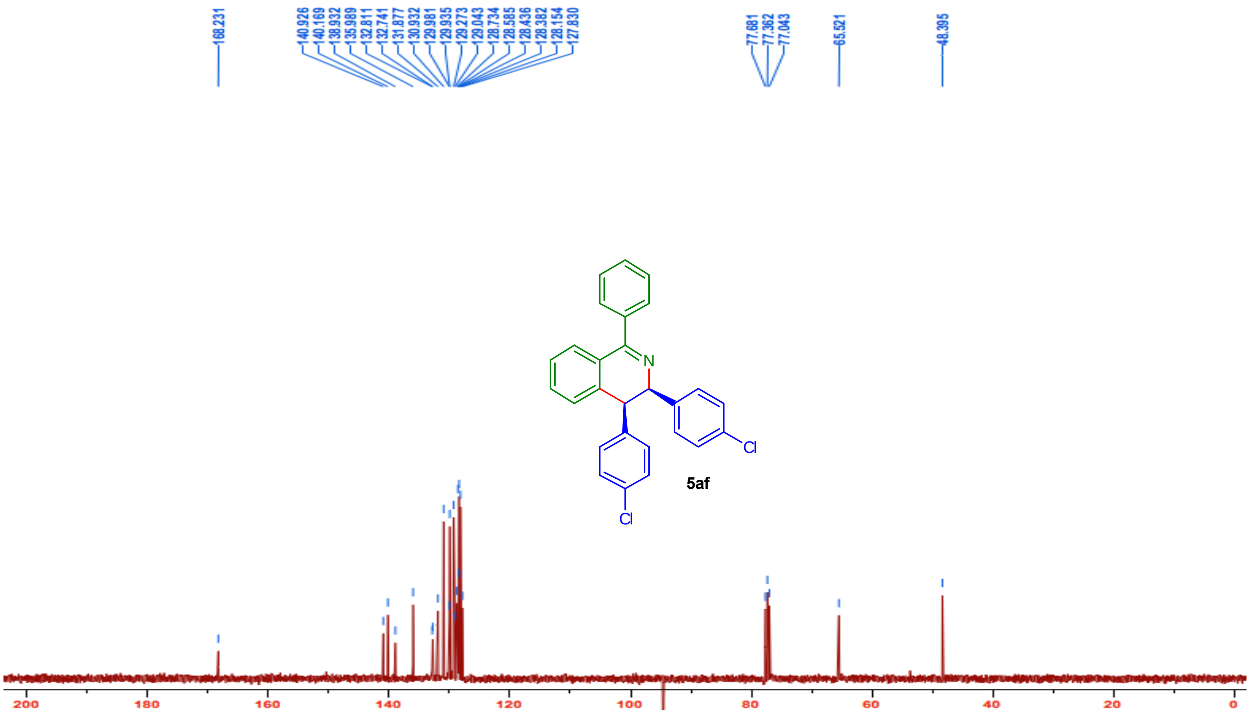

Supplementary Figure 14: ^{1}H NMR Spectrum of **5ag** (400 MHz, $CDCl_3$)

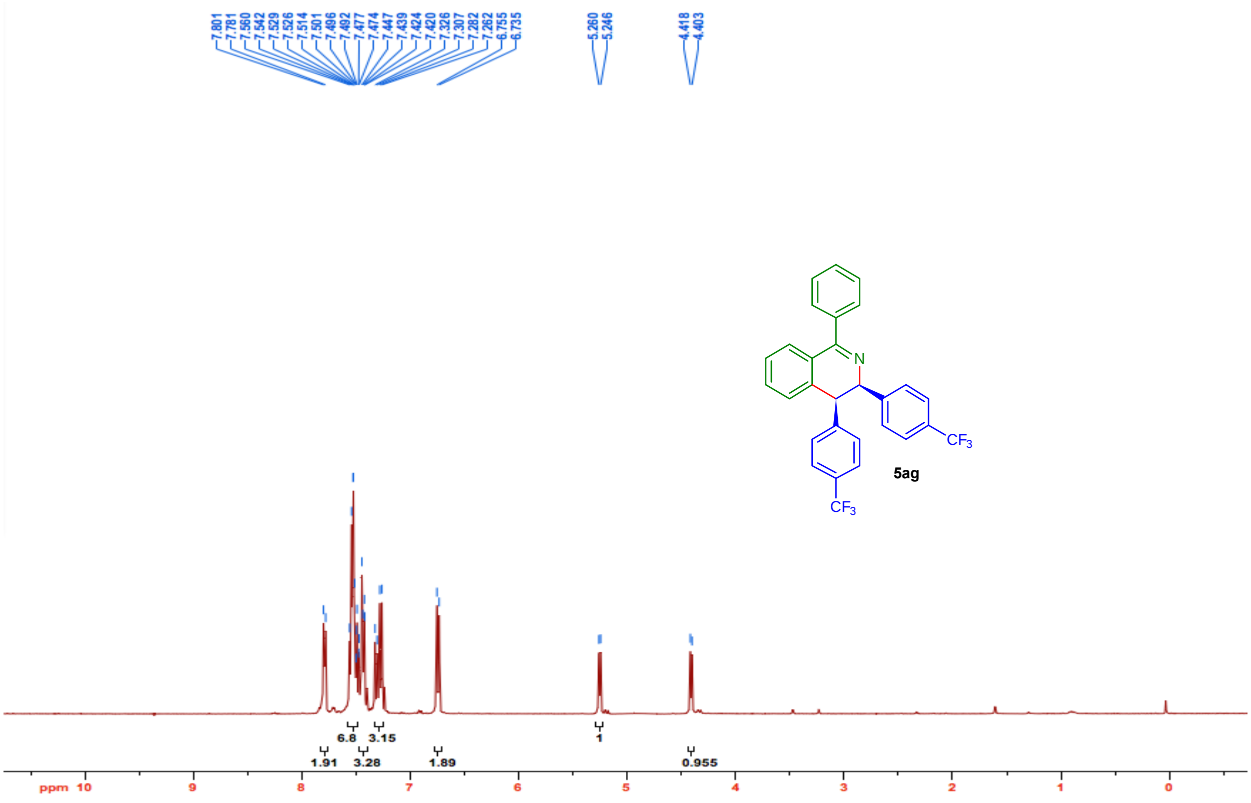

Supplementary Figure 15: ^{13}C NMR Spectrum of **5ag** (100 MHz, $CDCl_3$)

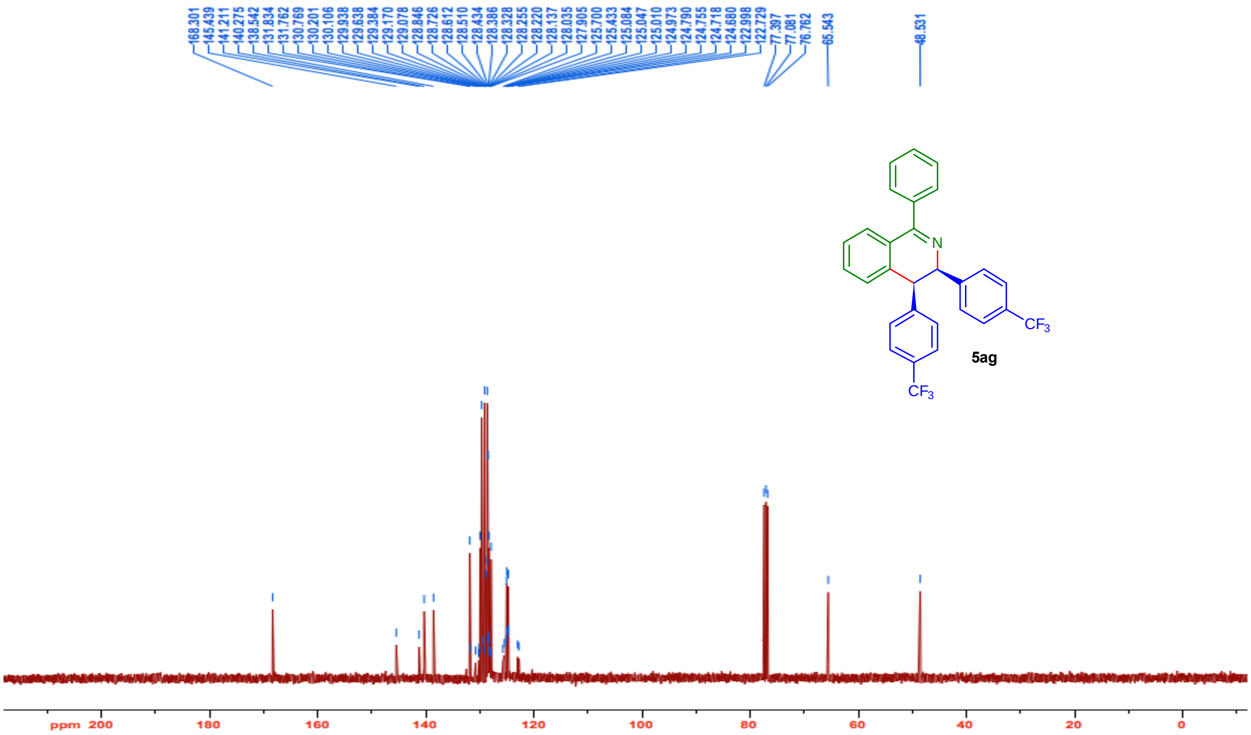

Supplementary Figure 16: ^{19}F NMR Spectrum of **5ag** (376 MHz, $CDCl_3$)

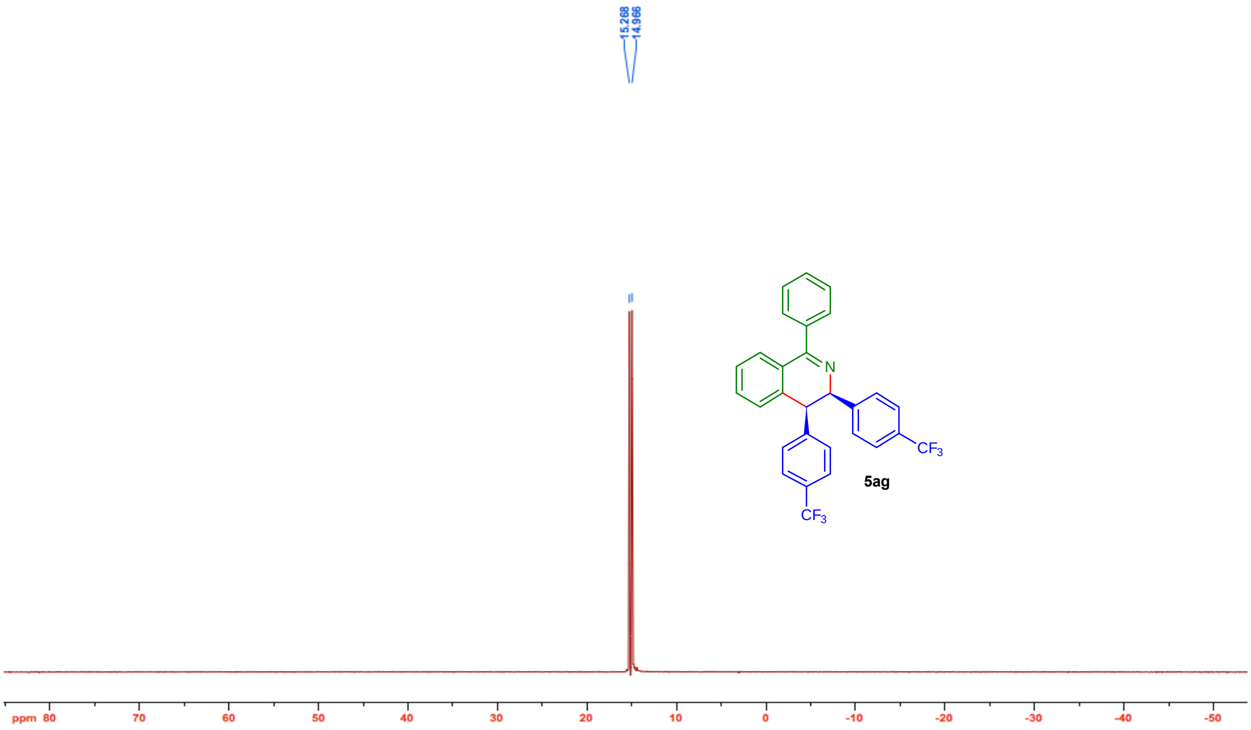

Supplementary Figure 17: ^{1}H NMR Spectrum of **5ah** (400 MHz, $CDCl_3$)

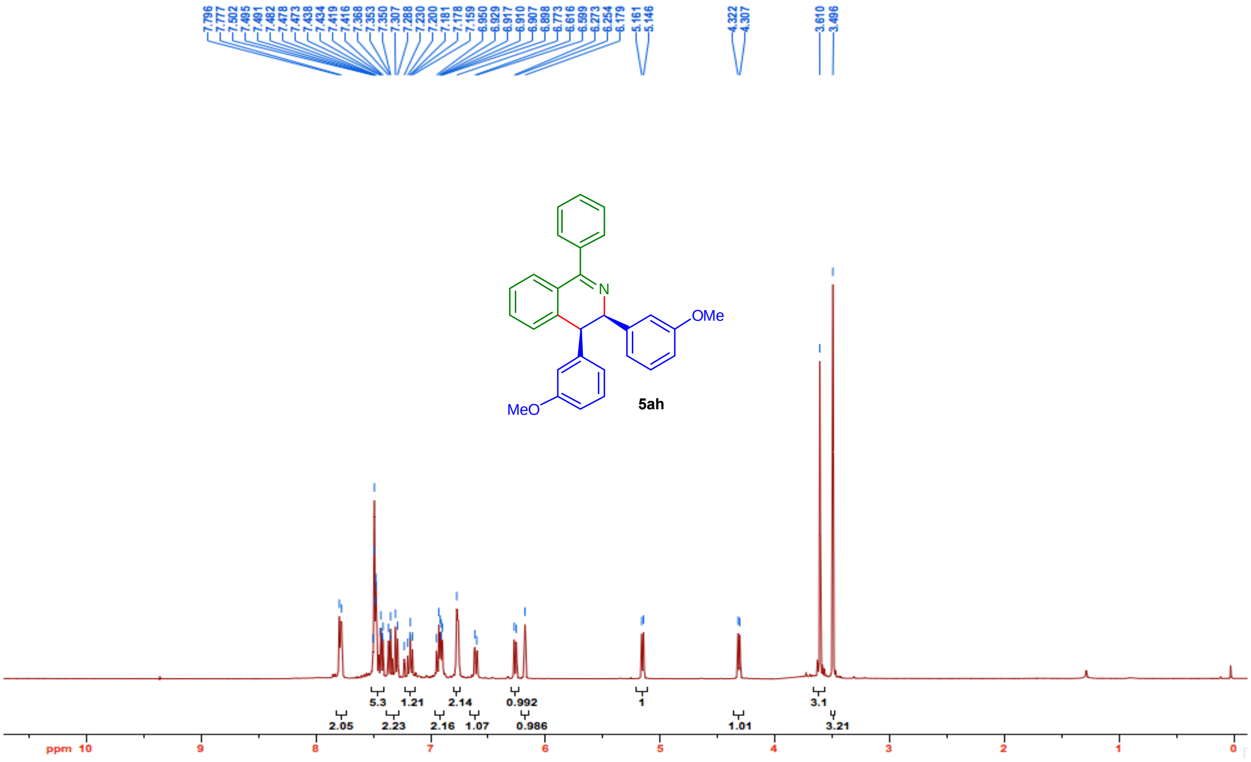

Supplementary Figure 18: ^{13}C NMR Spectrum of **5ah** (100 MHz, $CDCl_3$)

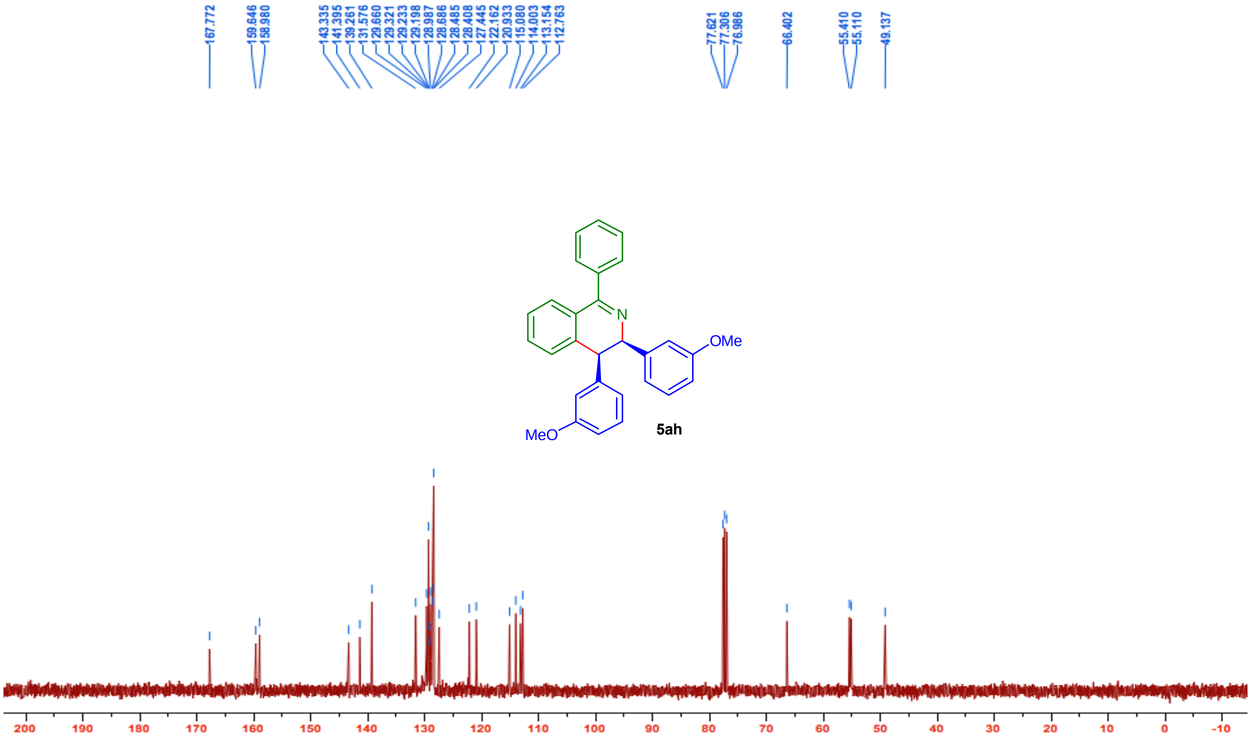

Supplementary Figure 19: ^{1}H NMR Spectrum of **5ai** (400 MHz, $CDCl_3$)

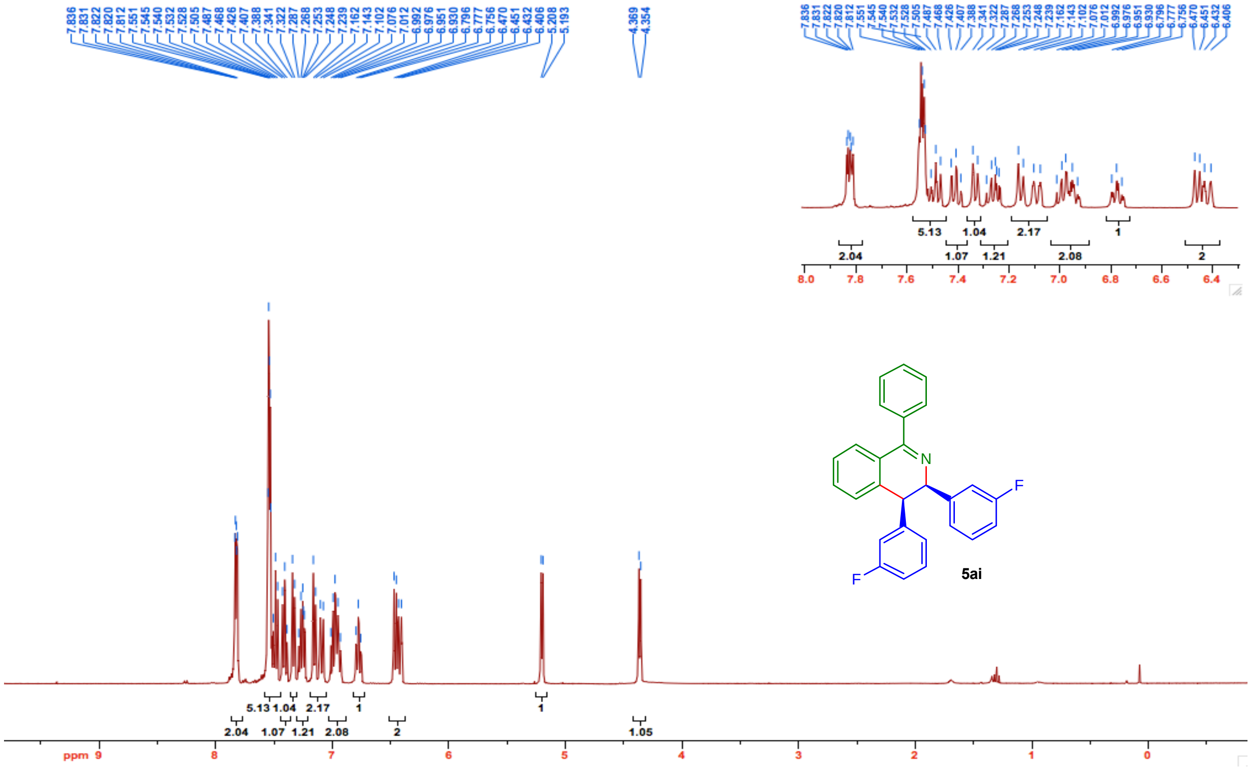

Supplementary Figure 20: ^{13}C NMR Spectrum of **5ai** (100 MHz, $CDCl_3$)

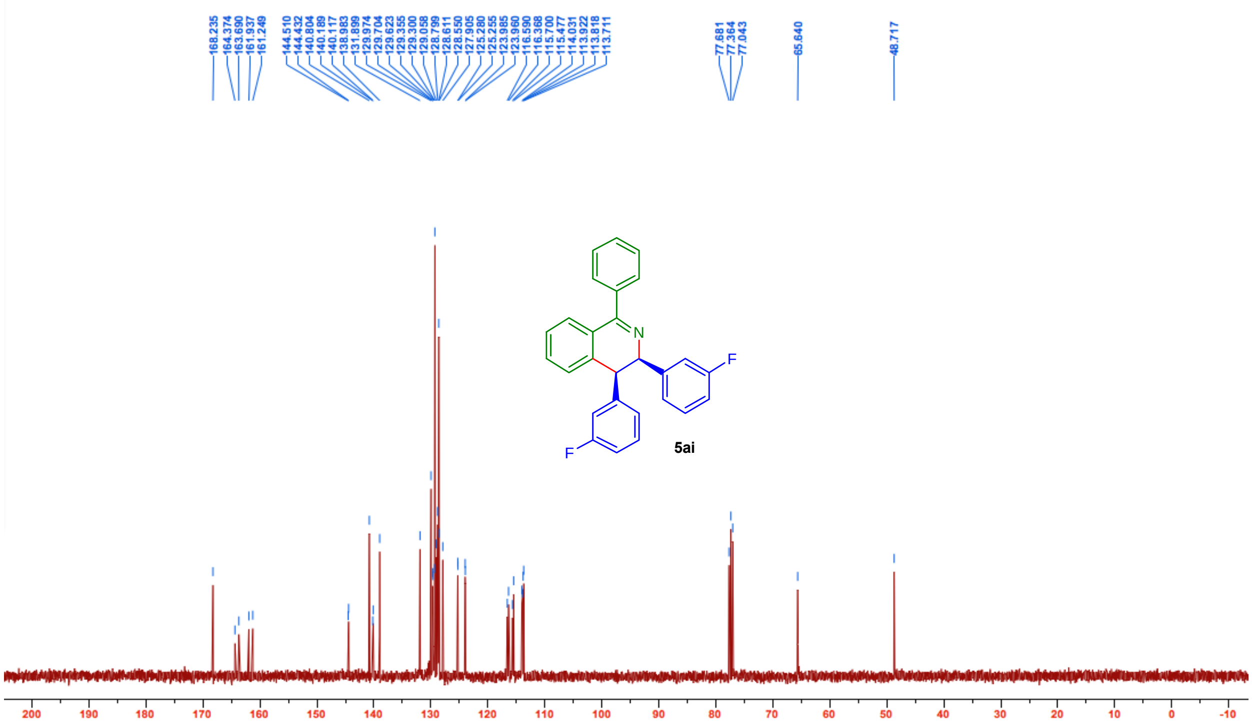

Supplementary Figure 21: ^{19}F NMR Spectrum of **5ai** (376 MHz, $CDCl_3$)

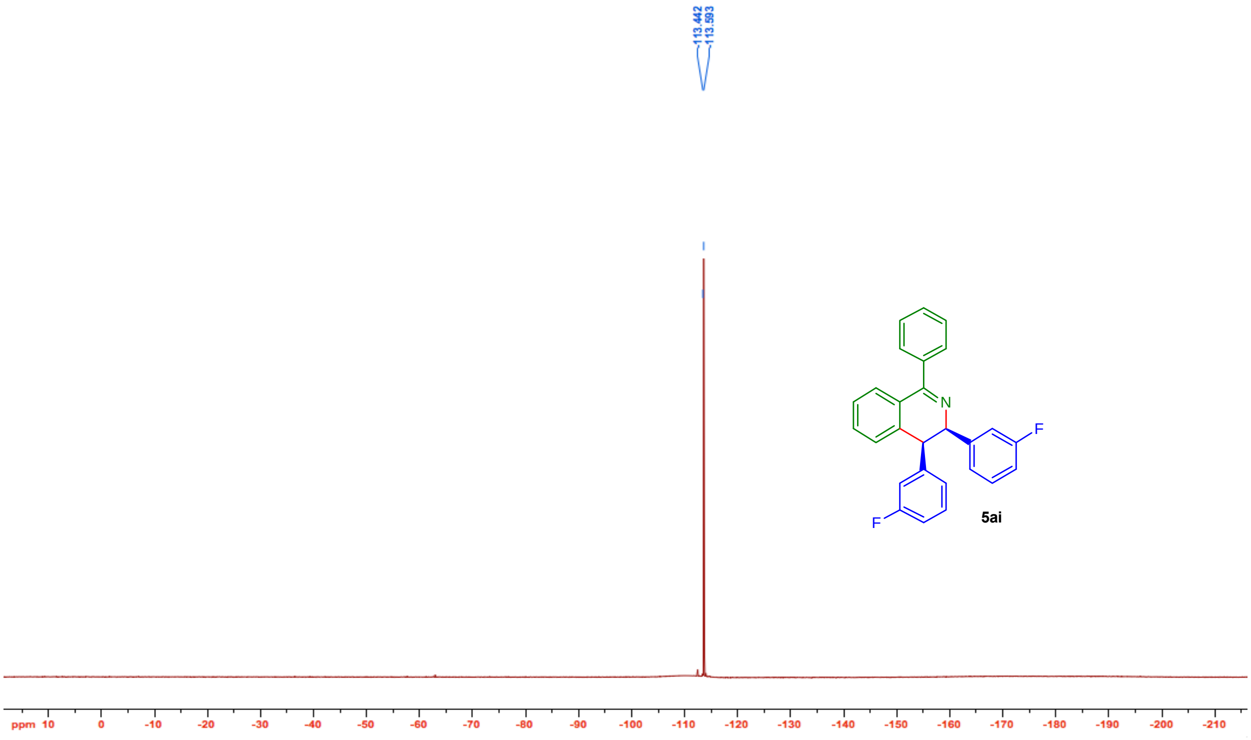

Supplementary Figure 22: ^{1}H NMR Spectrum of **5aj** (400 MHz, $CDCl_3$)

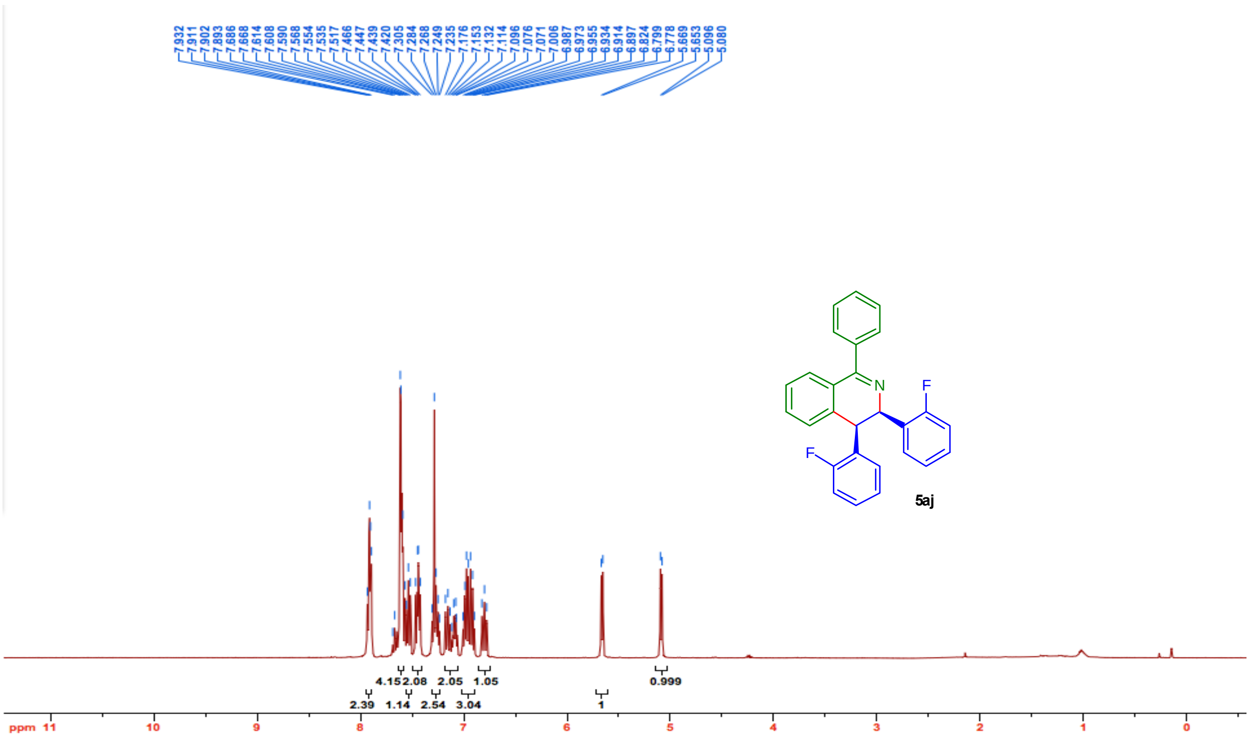

Supplementary Figure 23: ^{13}C NMR Spectrum of **5aj** (100 MHz, $CDCl_3$)

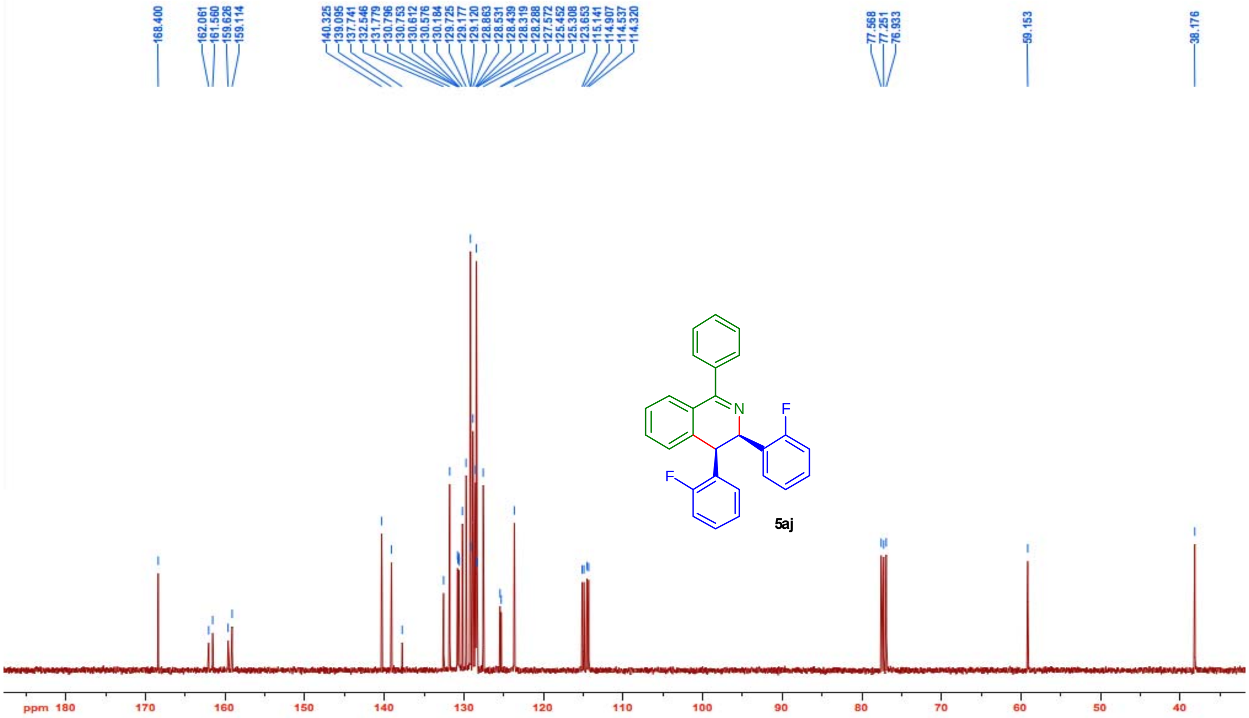

Supplementary Figure 24: ^{19}F NMR Spectrum of **5aj** (376 MHz, $CDCl_3$)

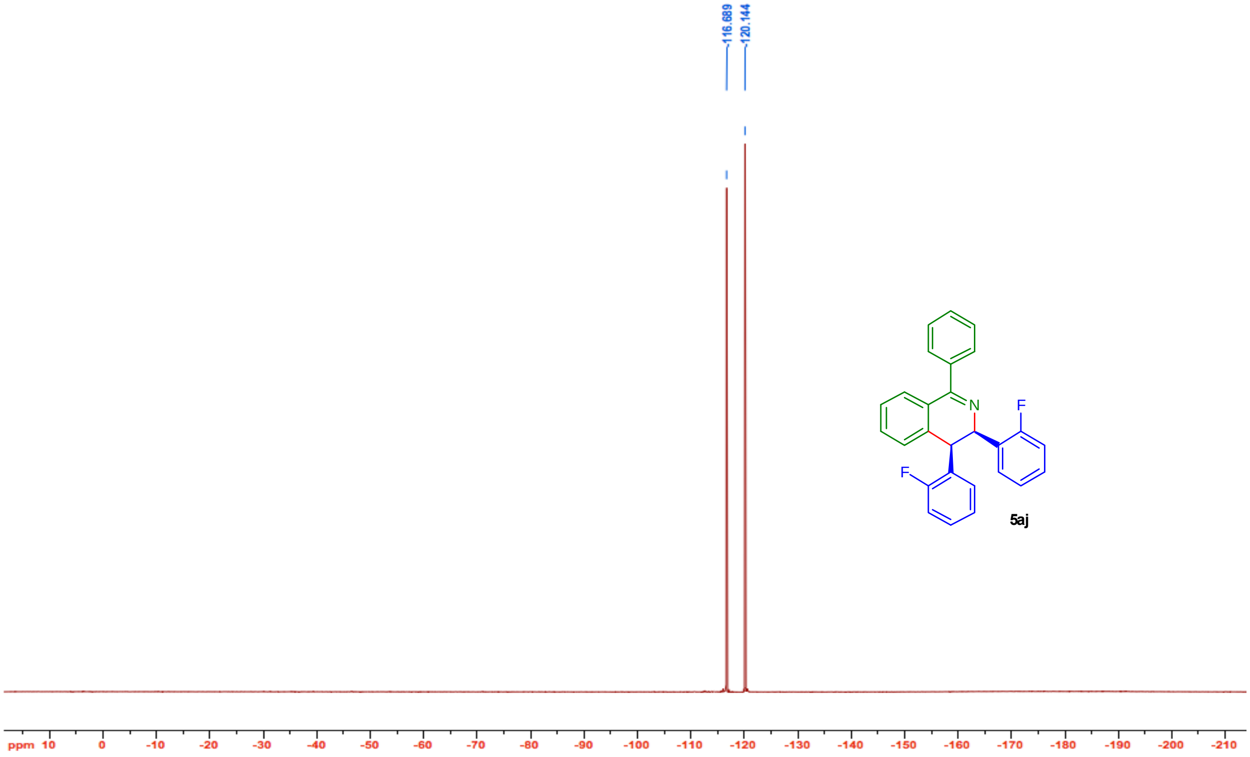

Supplementary Figure 25: ^{1}H NMR Spectrum of **5ak** (400 MHz, $CDCl_3$)

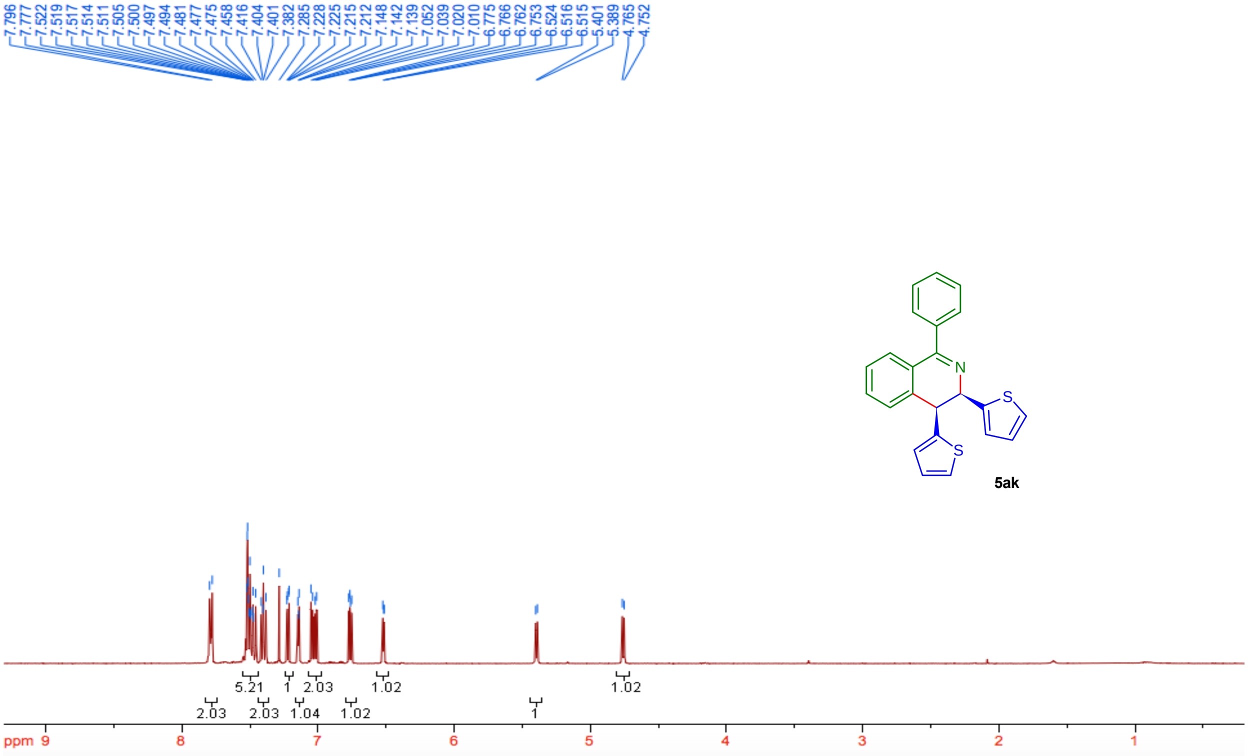

Supplementary Figure 26: ^{13}C NMR Spectrum of **5ak** (100 MHz, $CDCl_3$)

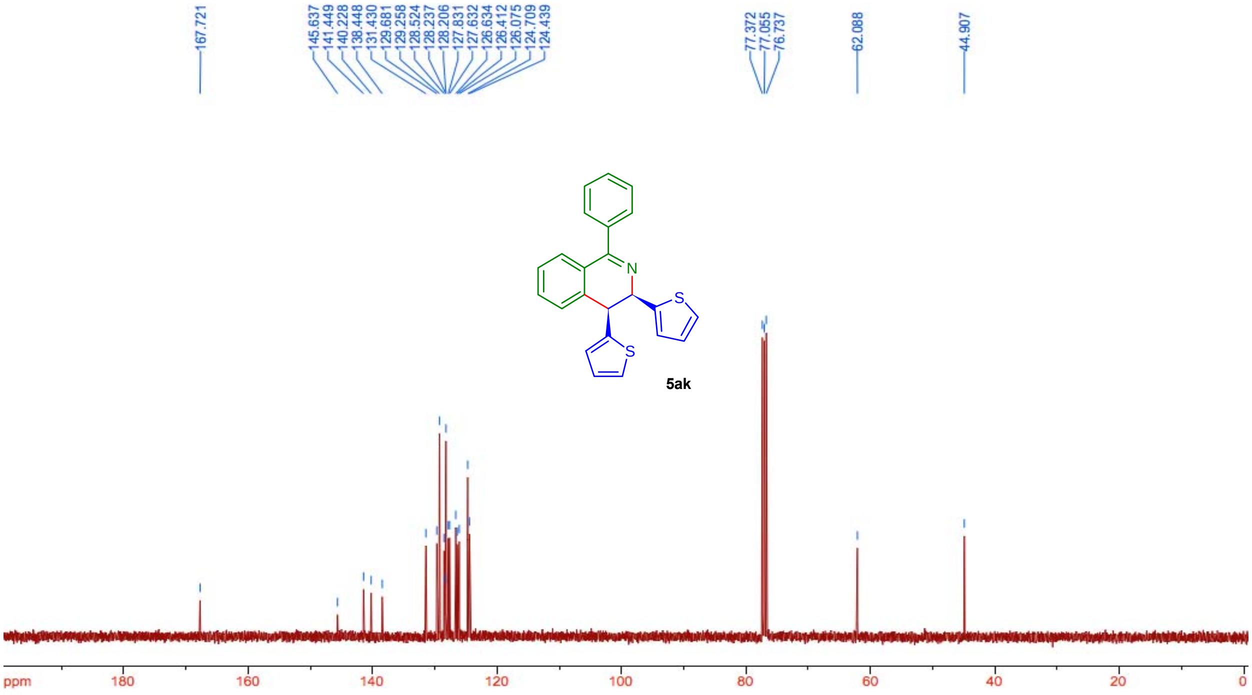

Supplementary Figure 27: ^{1}H NMR Spectrum of **5al** (400 MHz, $CDCl_3$)

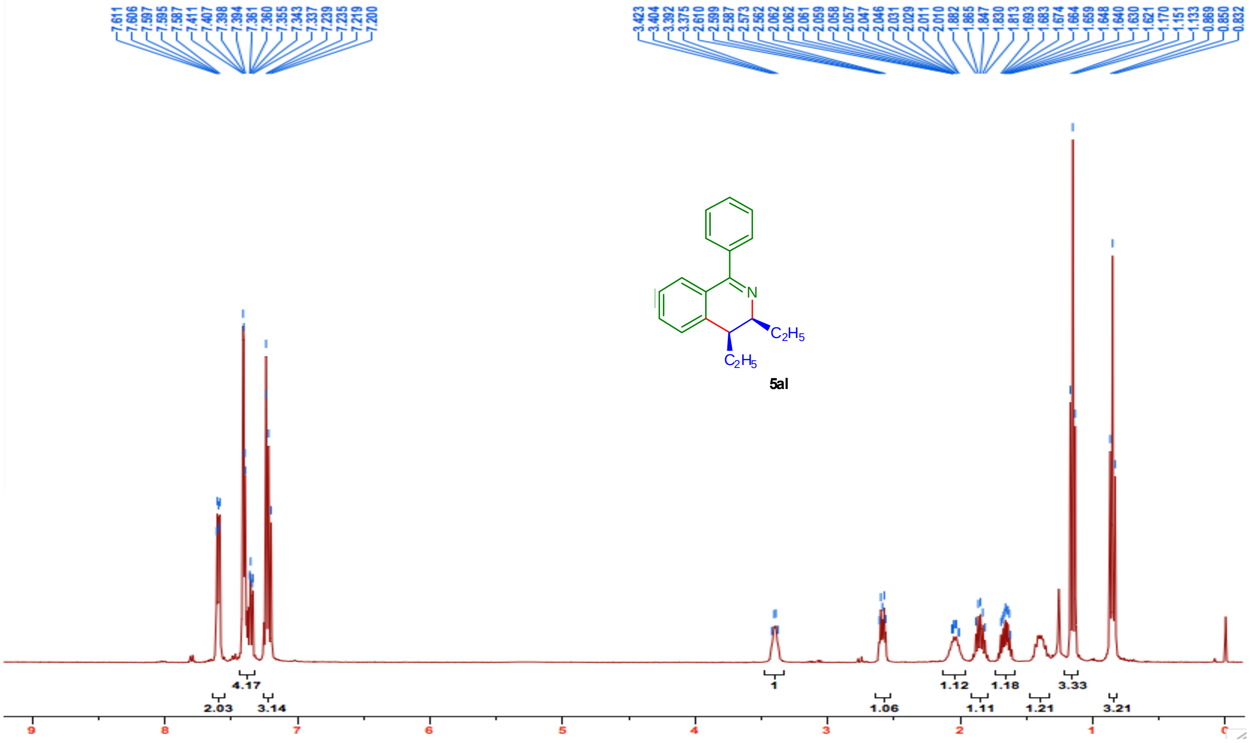

Supplementary Figure 28: ^{13}C NMR Spectrum of **5al** (100 MHz, $CDCl_3$)

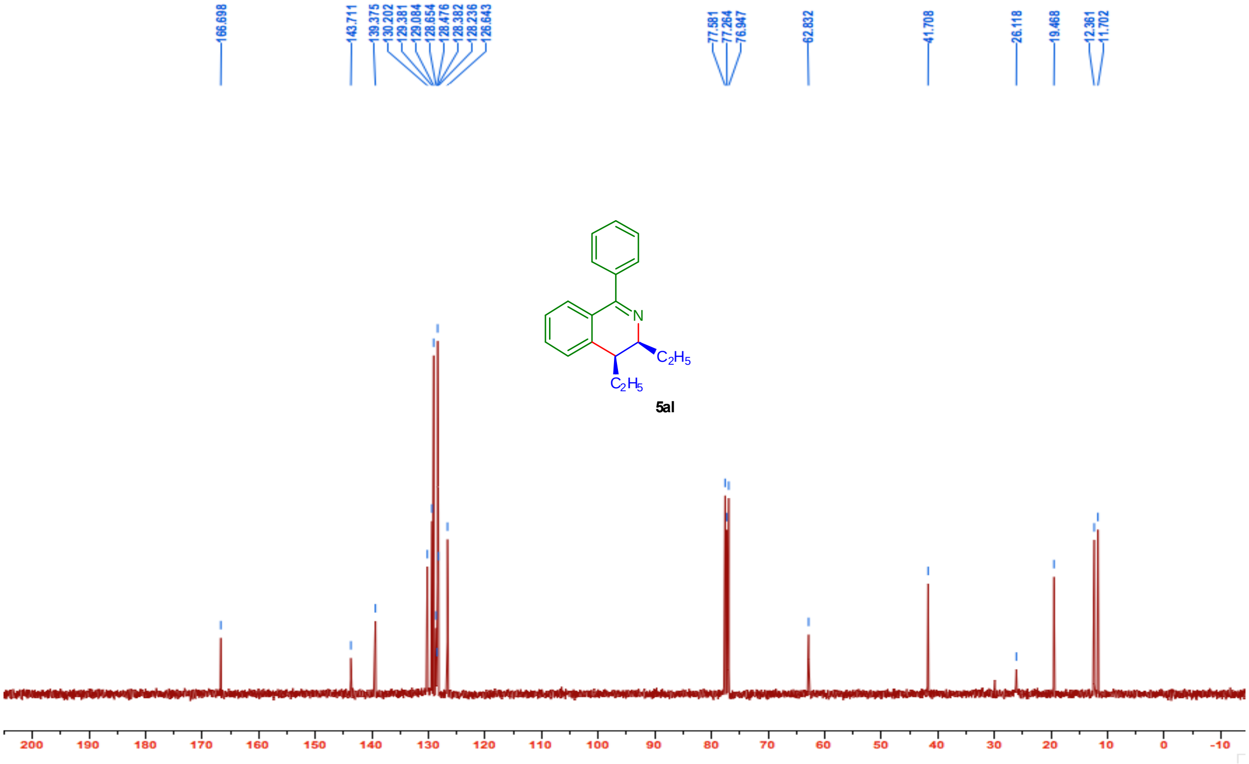

Supplementary Figure 29: ^{1}H NMR Spectrum of **5am** (400 MHz, $CDCl_3$)

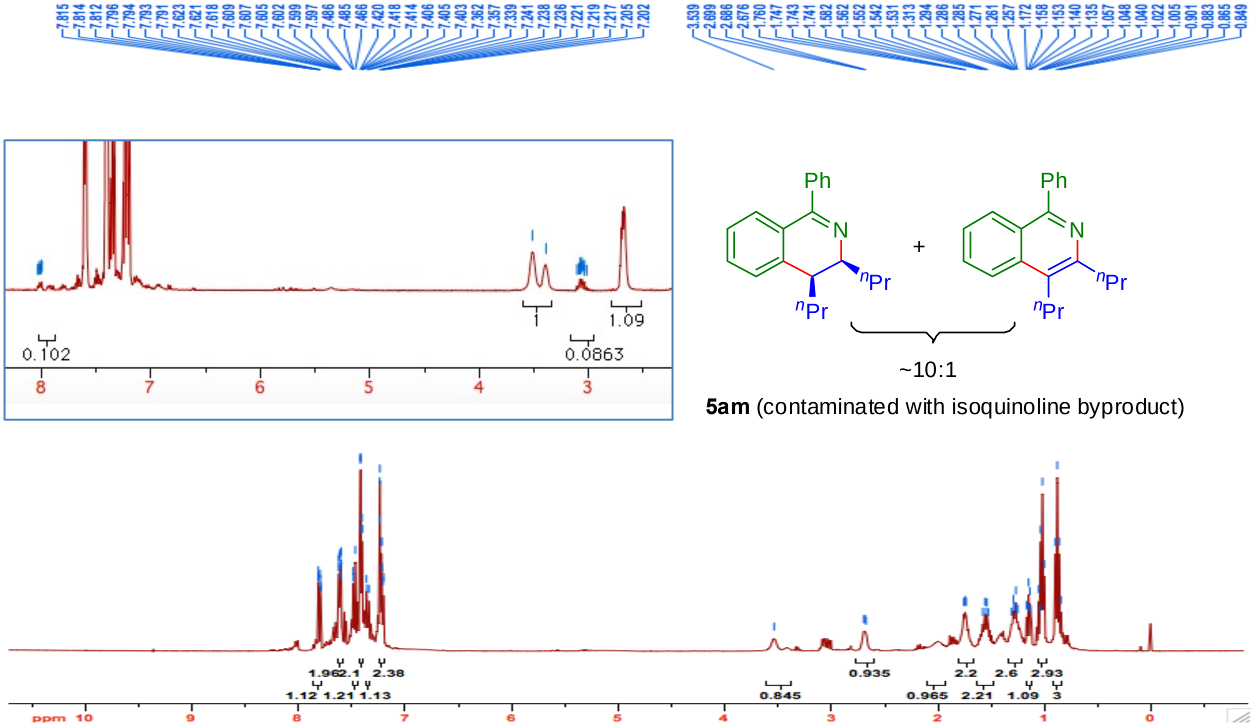

Supplementary Figure 30: ^{13}C NMR Spectrum of **5am** (100 MHz, $CDCl_3$)

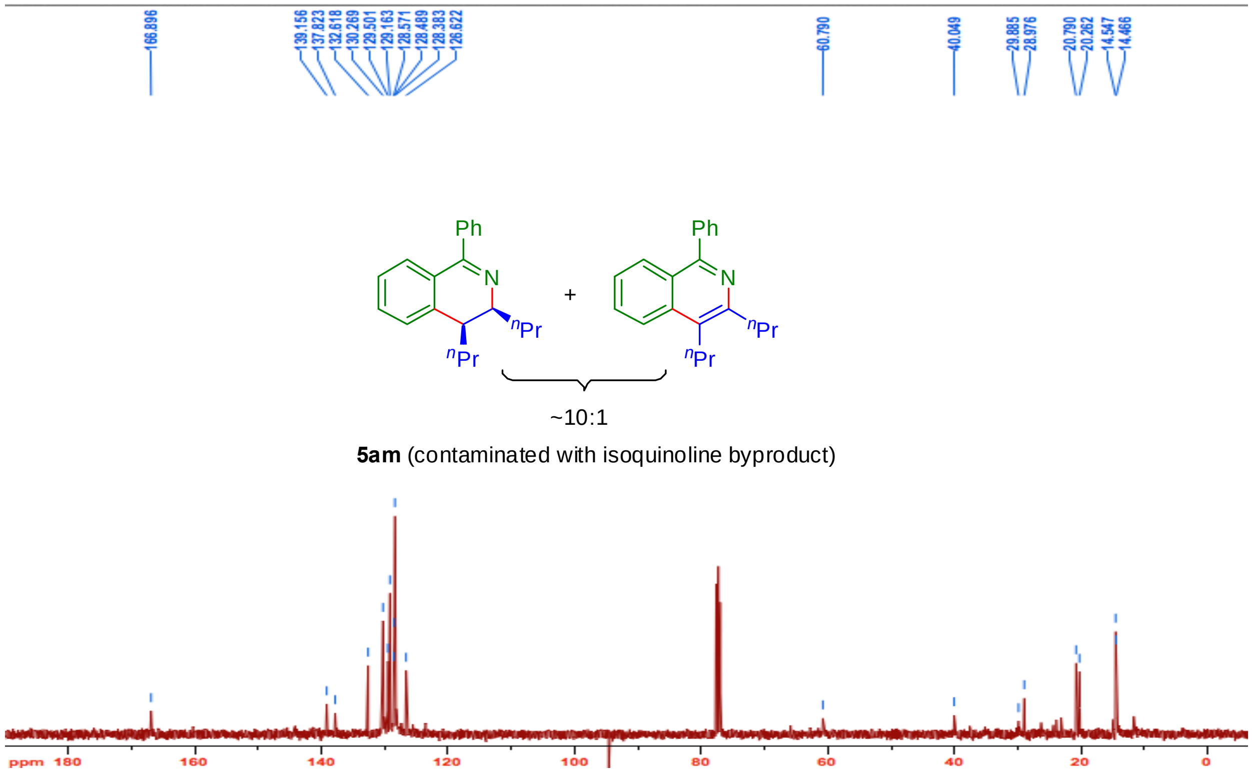

Supplementary Figure 31: ^{1}H NMR Spectrum of **5an** (400 MHz, $CDCl_3$)

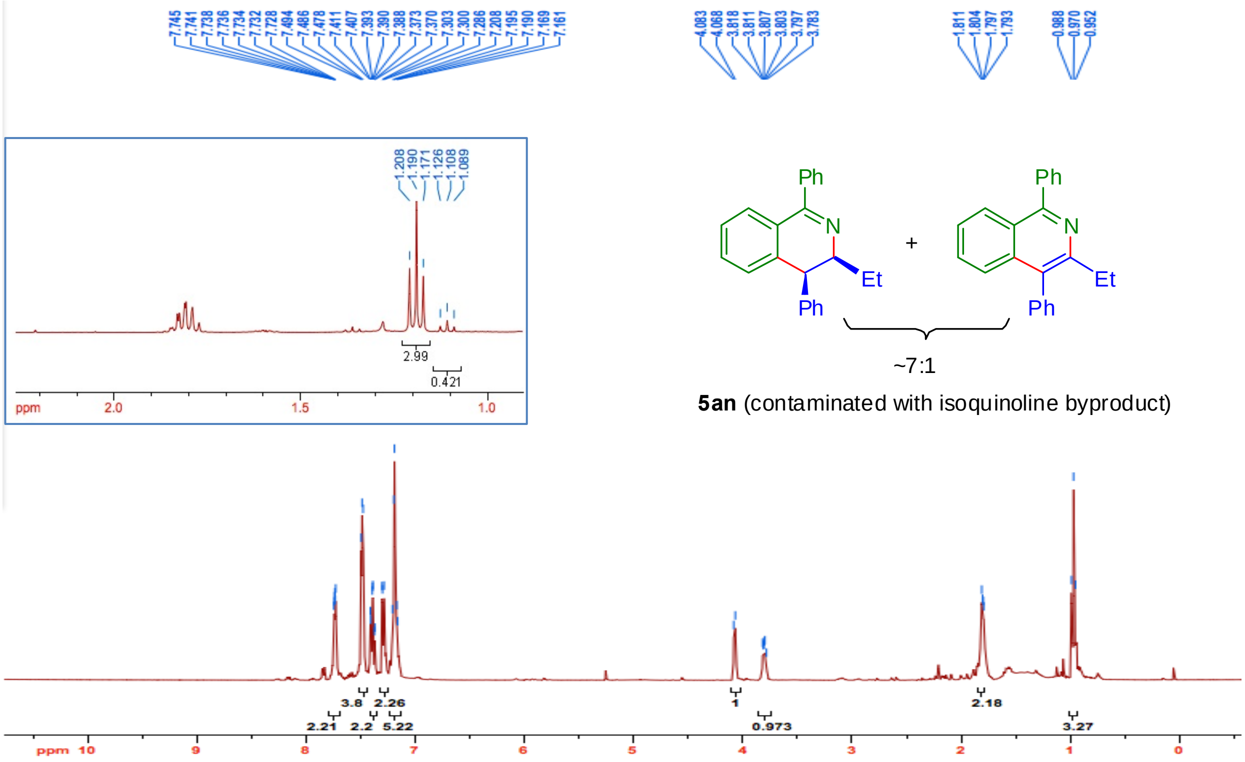

Supplementary Figure 32: ^{13}C NMR Spectrum of **5an** (100 MHz, $CDCl_3$)

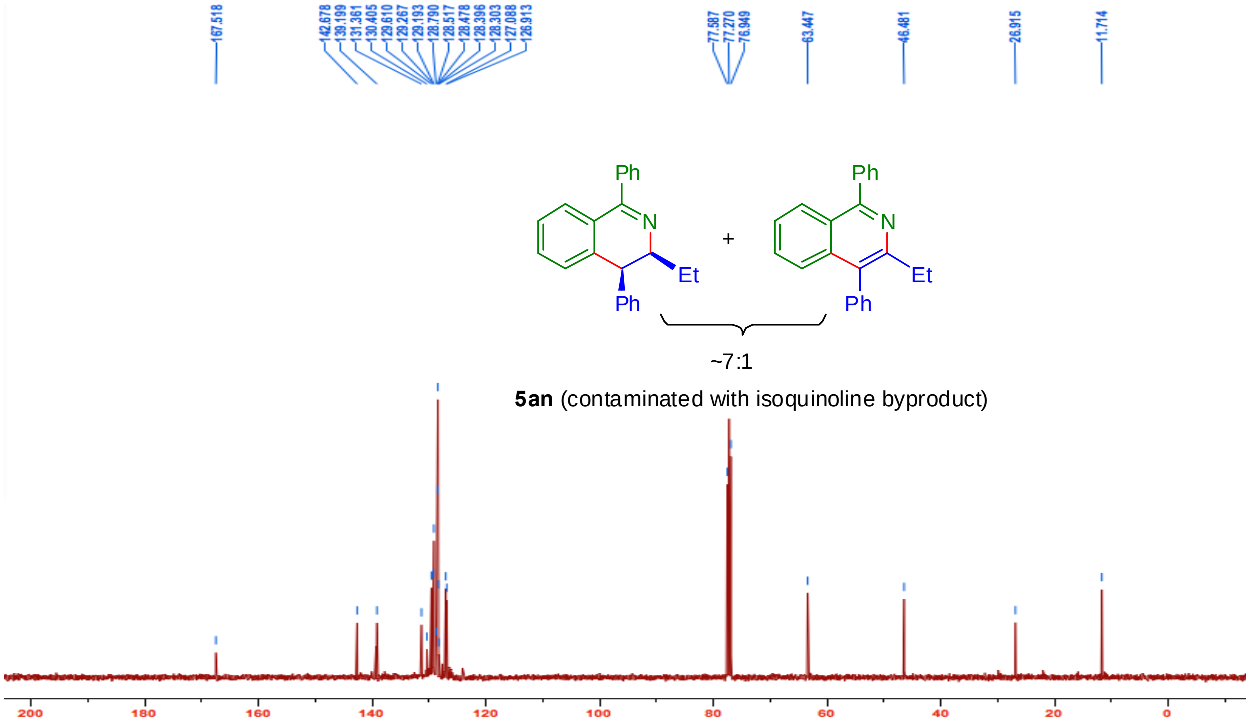

Supplementary Figure 33: ^{1}H NMR Spectrum of **5ao** (400 MHz, $CDCl_3$)

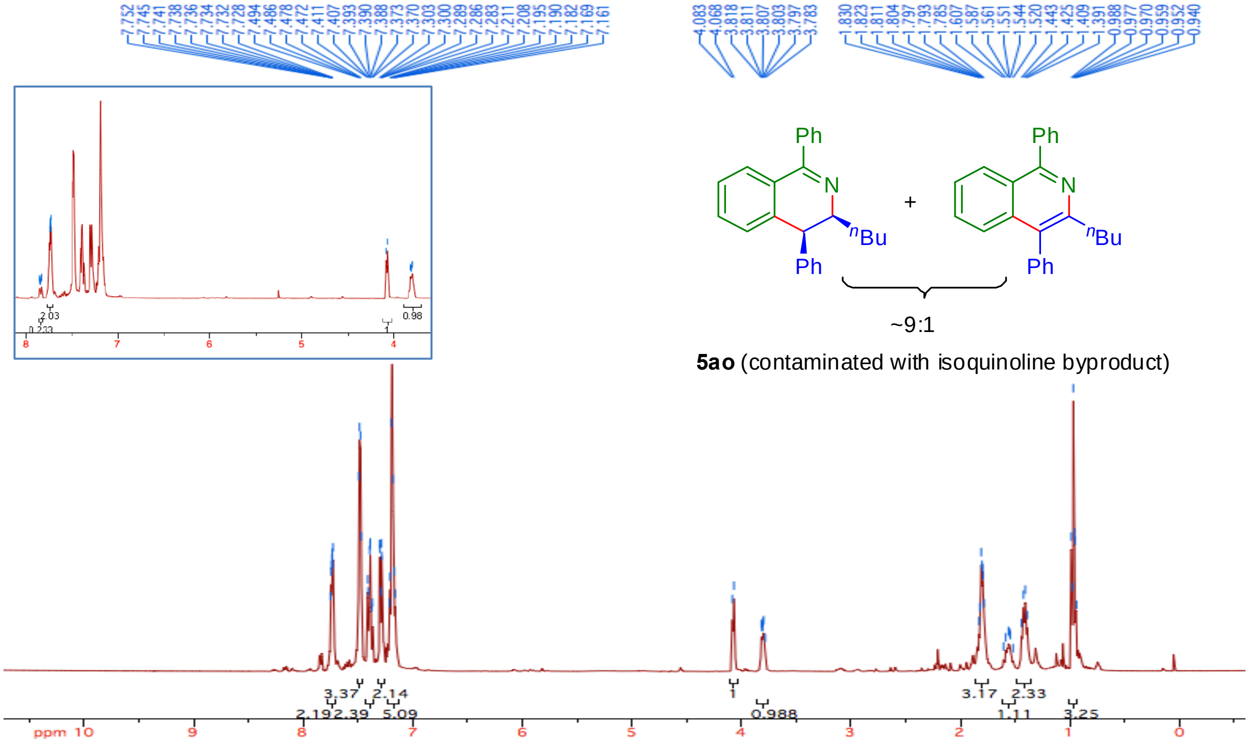

Supplementary Figure 34: ^{13}C NMR Spectrum of **5ao** (100 MHz, $CDCl_3$)

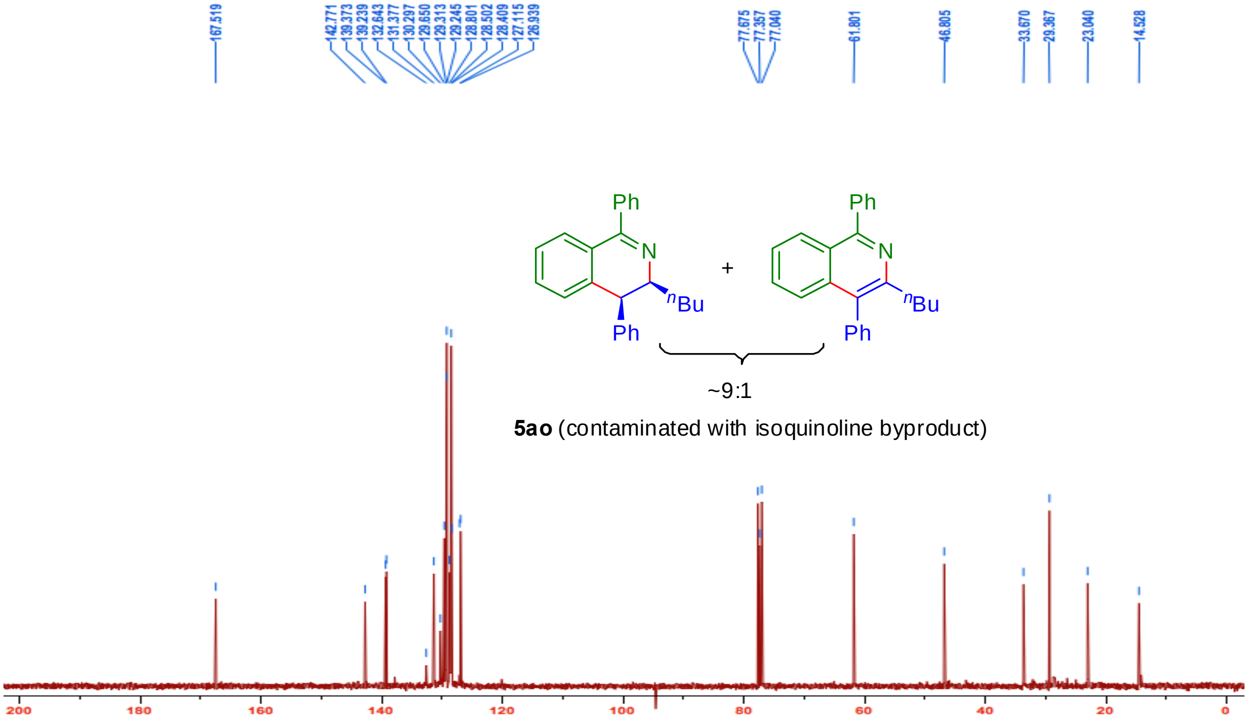

Supplementary Figure 35: ^{1}H NMR Spectrum of **5ap** (400 MHz, $CDCl_3$)

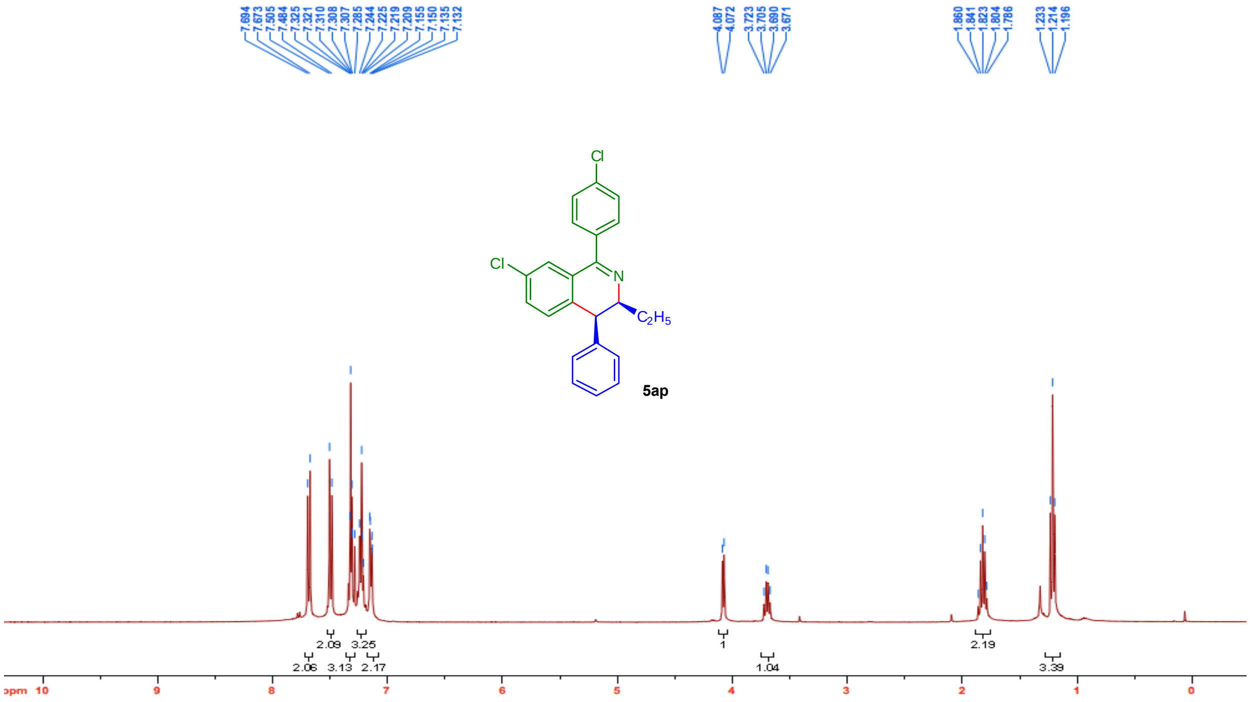

Supplementary Figure 36: ^{13}C NMR Spectrum of **5ap** (100 MHz, $CDCl_3$)

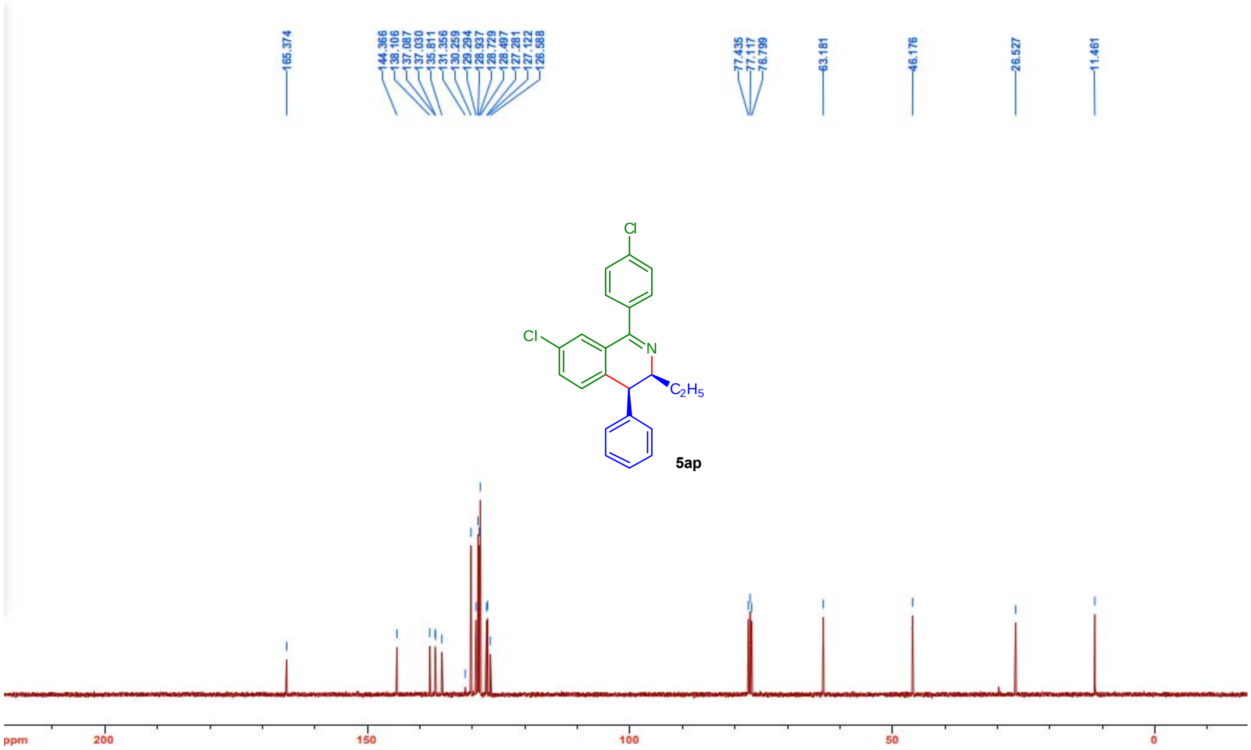

Supplementary Figure 37: ^{1}H NMR Spectrum of **5aq** (400 MHz, $CDCl_3$)

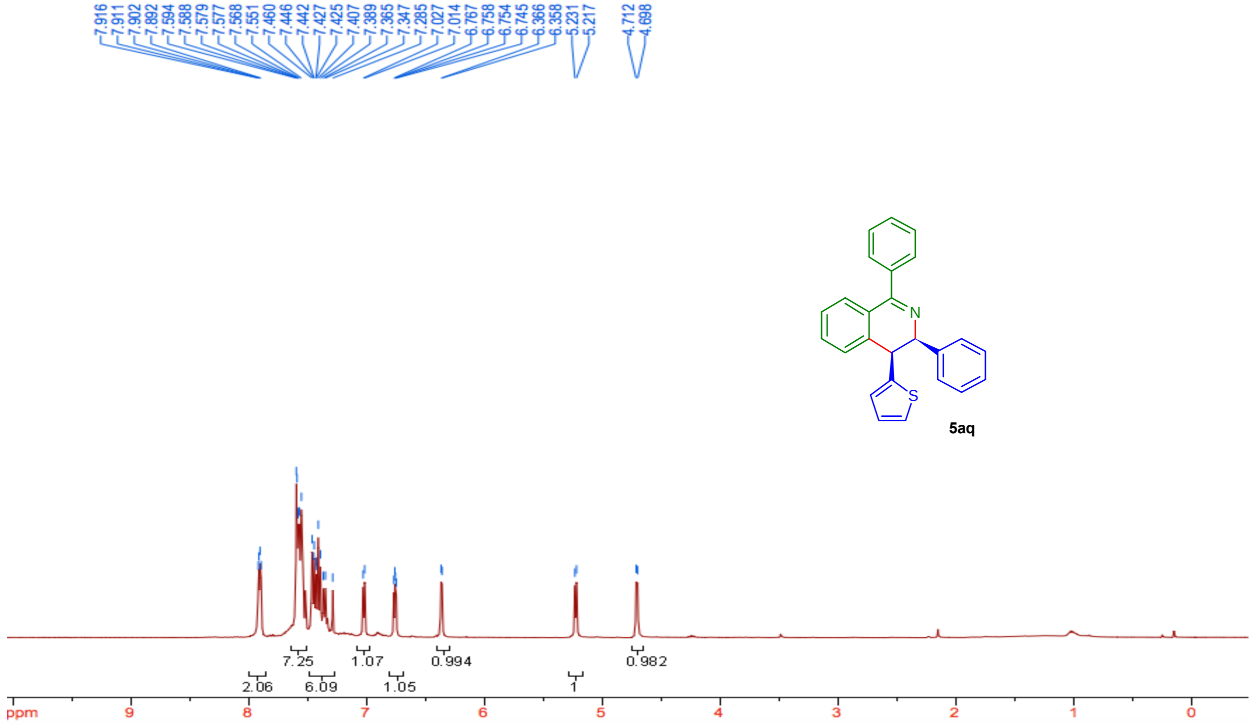

Supplementary Figure 38: ^{13}C NMR Spectrum of **5aq** (100 MHz, $CDCl_3$)

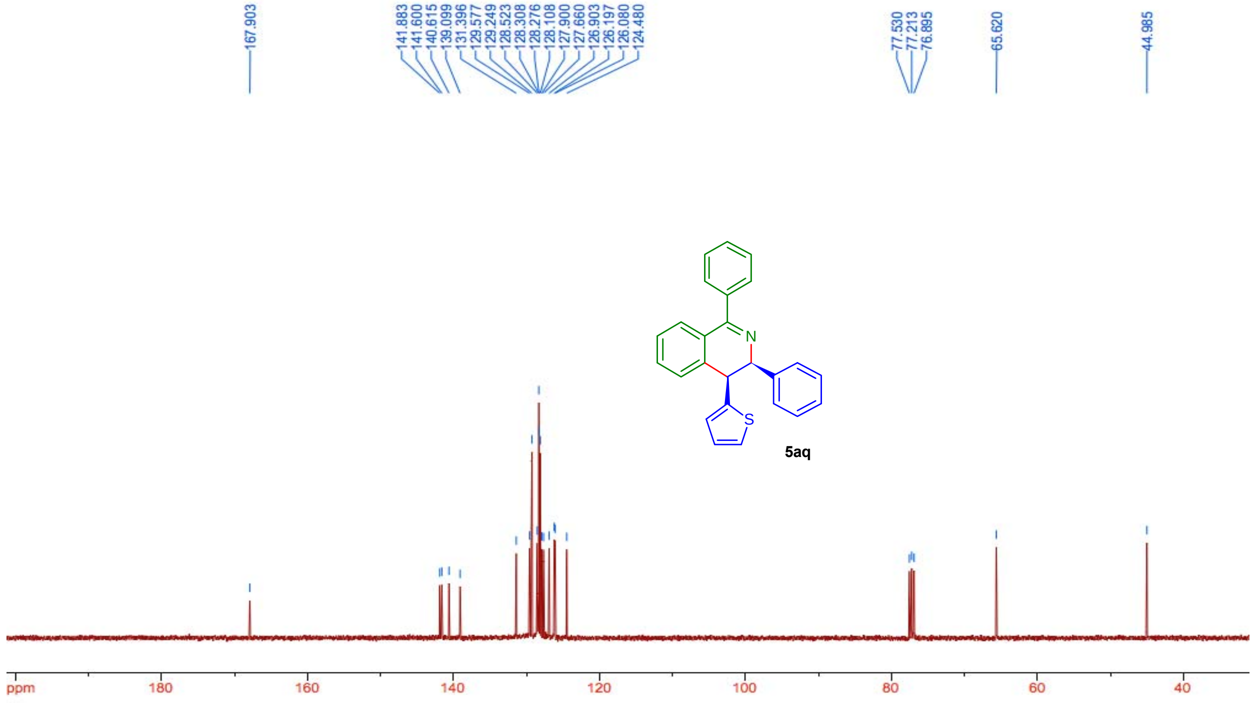

Supplementary Figure 39: 2D NOESY Spectrum of **5aq** (400 MHz, $CDCl_3$)

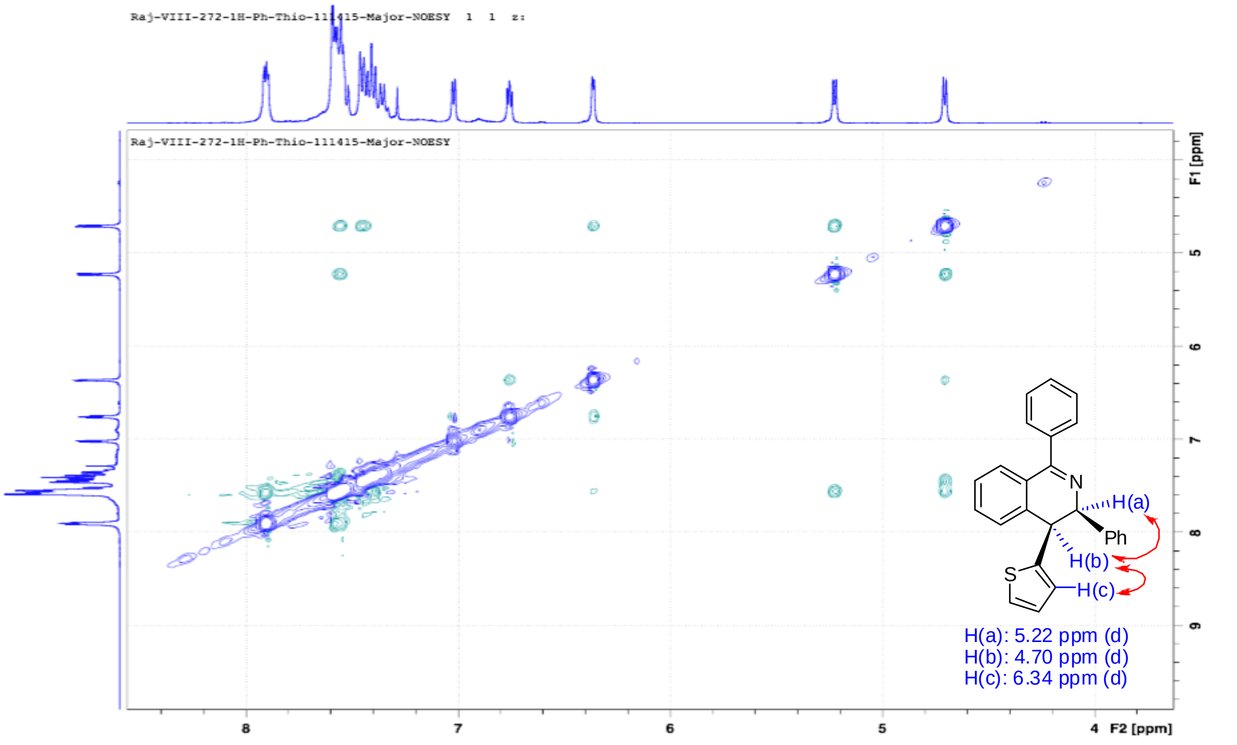

Supplementary Figure 40: ^{1}H NMR Spectrum of **5ba** (400 MHz, $CDCl_3$)

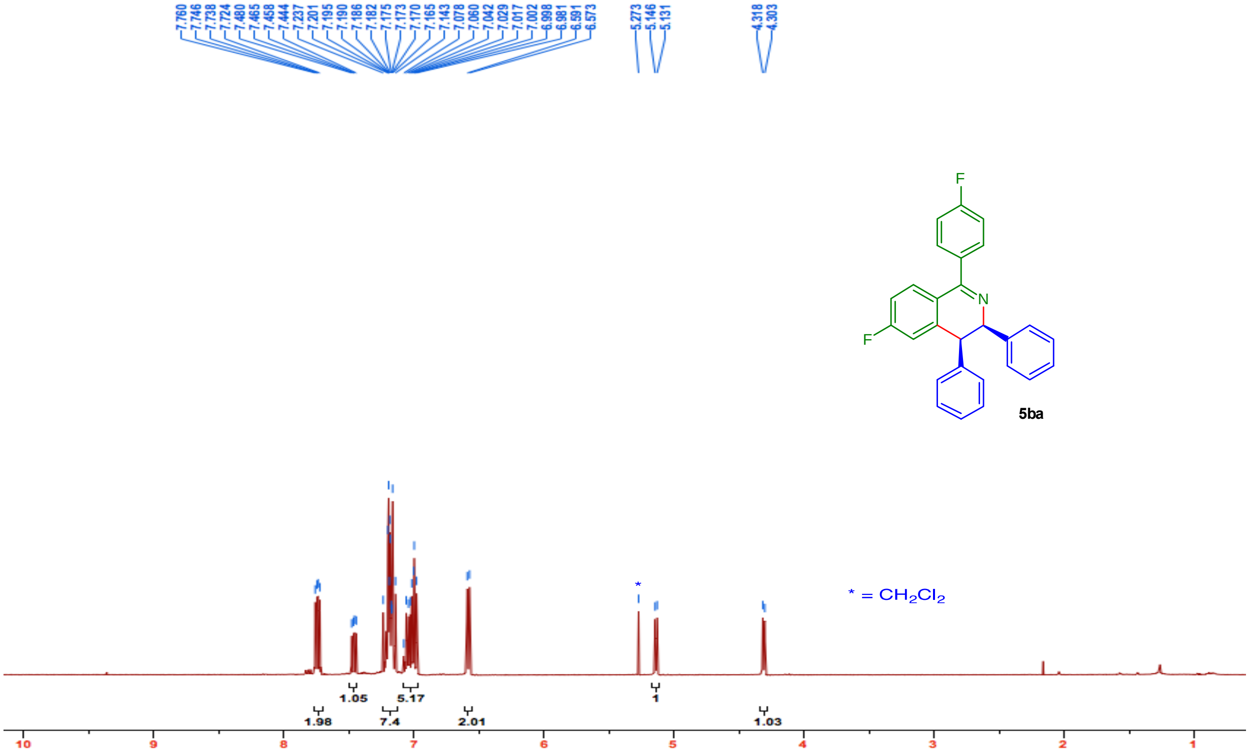

Supplementary Figure 41: ^{13}C NMR Spectrum of **5ba** (100 MHz, $CDCl_3$)

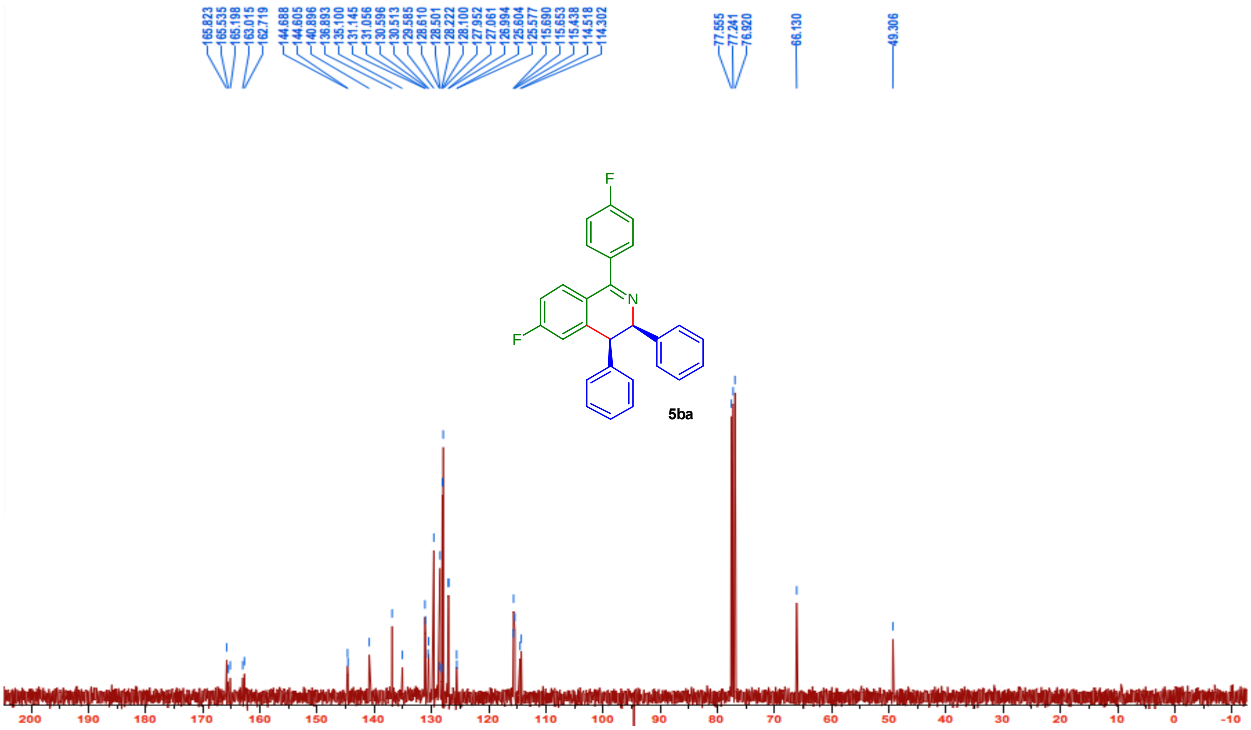

Supplementary Figure 42: ^{19}F NMR Spectrum of **5ba** (376 MHz, $CDCl_3$)

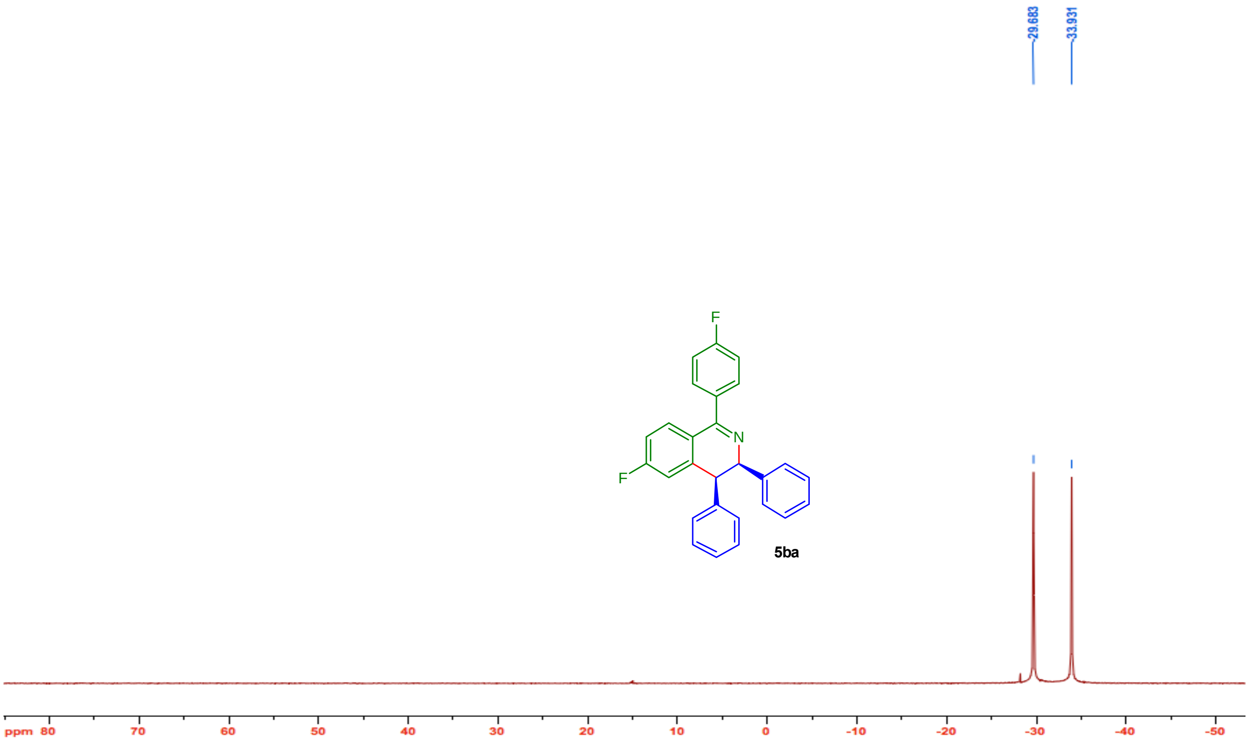

Supplementary Figure 43: ^{1}H NMR Spectrum of **5ca** (400 MHz, $CDCl_3$)

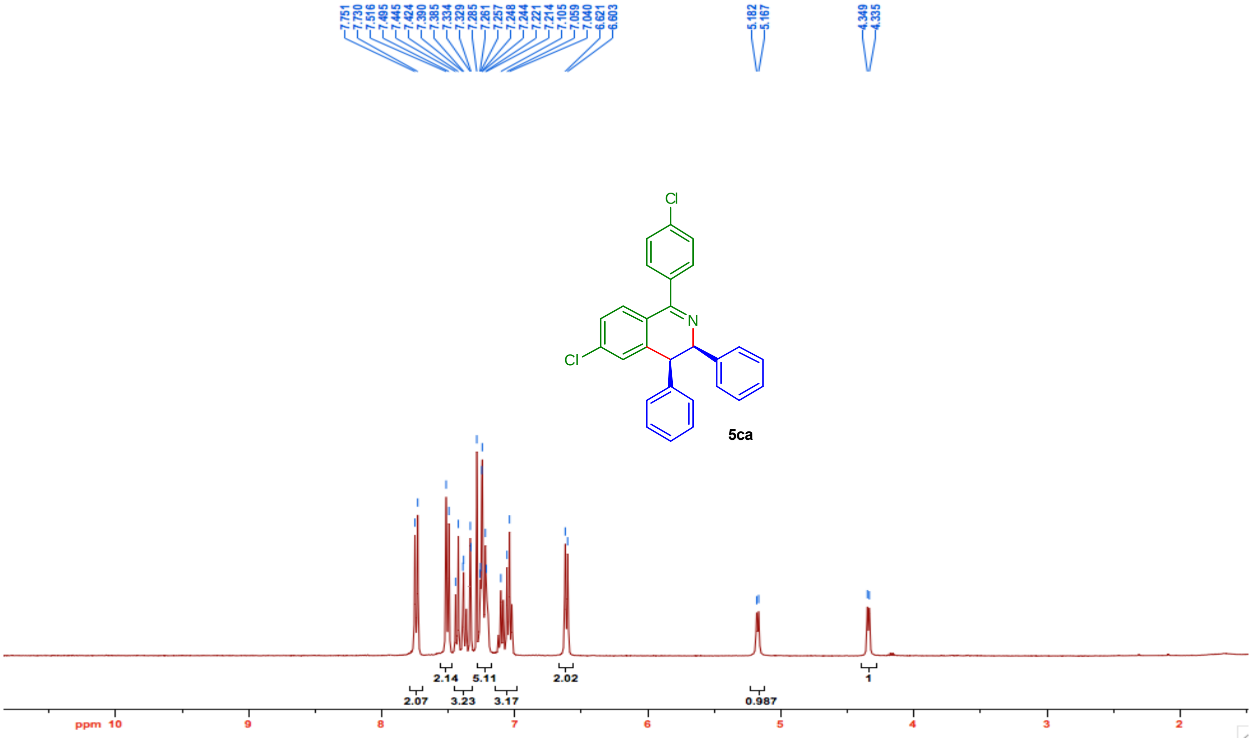

Supplementary Figure 44: ^{13}C NMR Spectrum of **5ca** (100 MHz, $CDCl_3$)

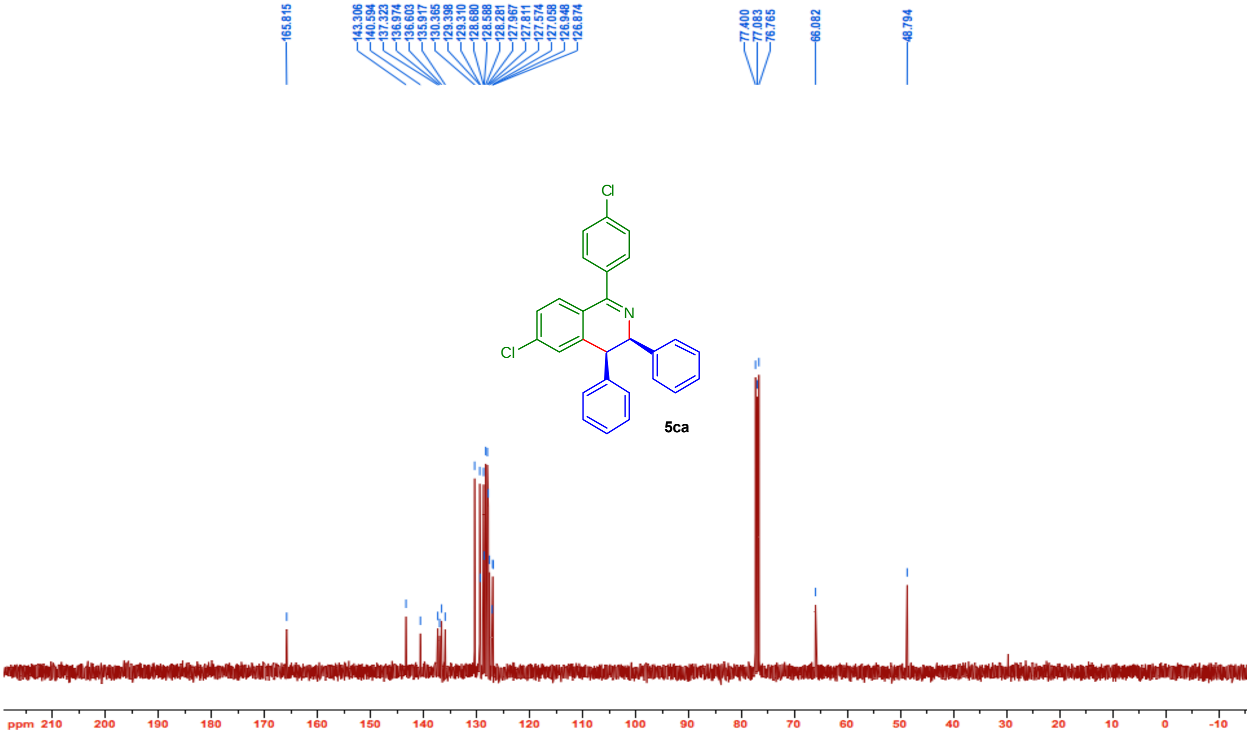

Supplementary Figure 45: ^{1}H NMR Spectrum of **5da** (400 MHz, $CDCl_3$)

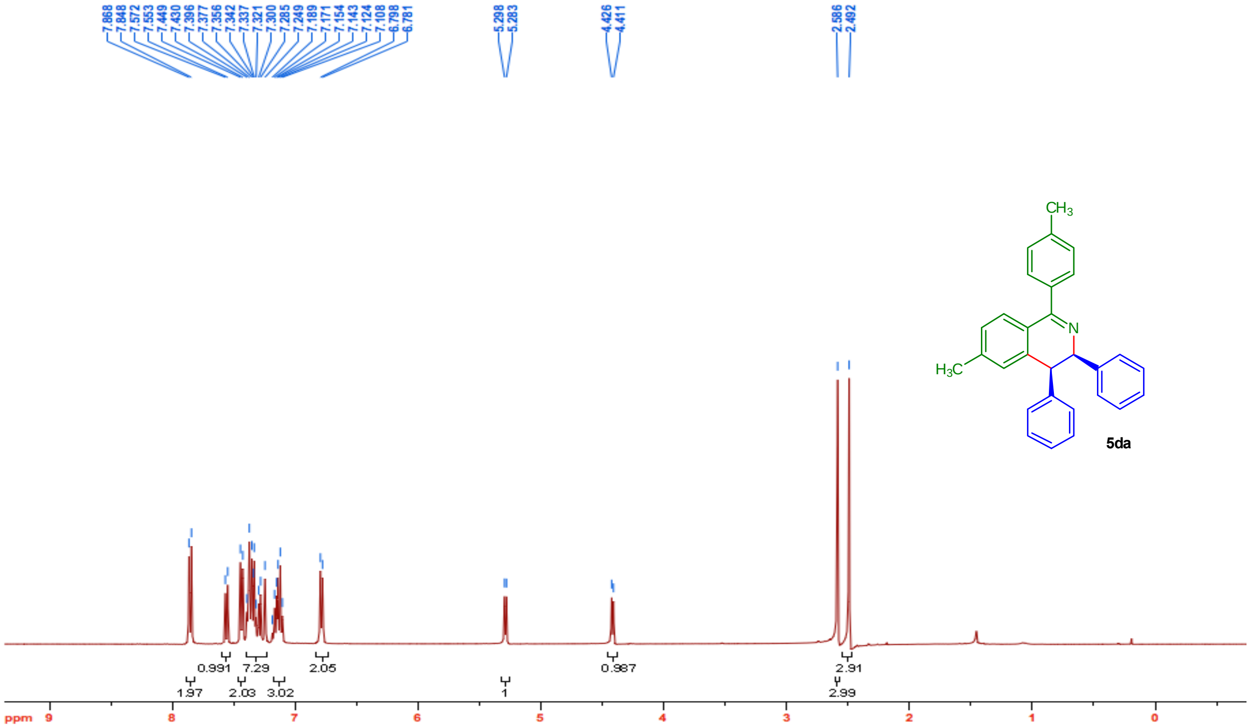

Supplementary Figure 46: ^{13}C NMR Spectrum of **5da** (100 MHz, $CDCl_3$)

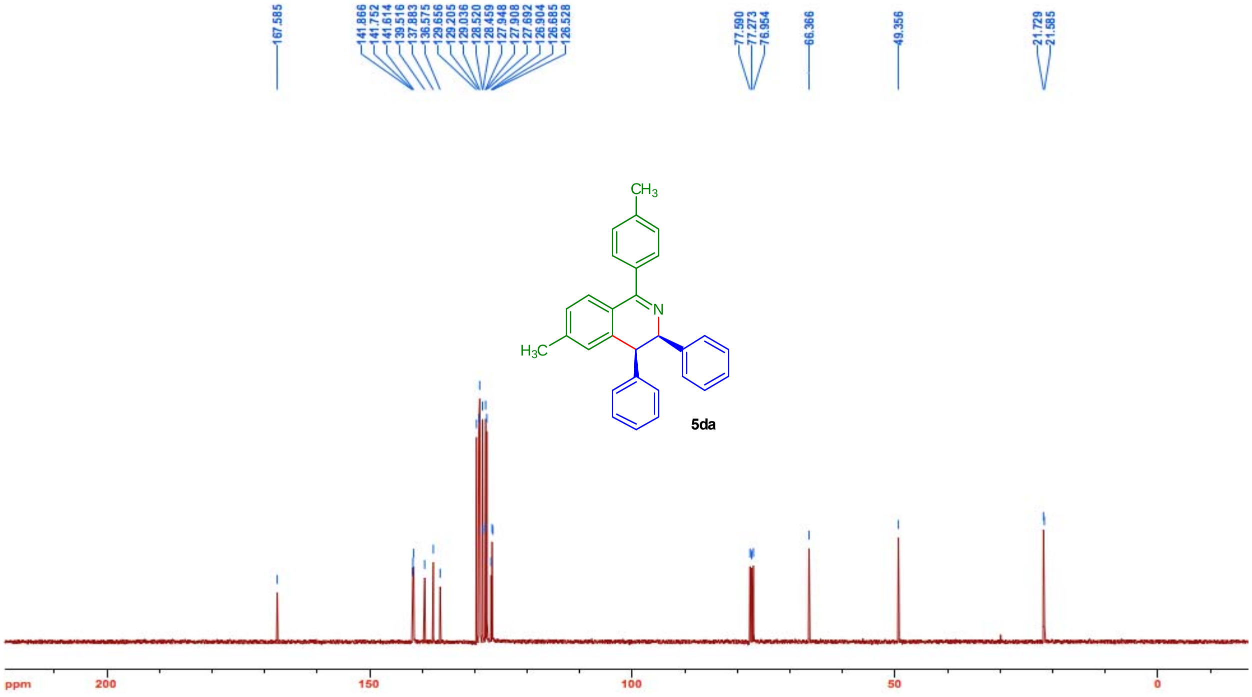

Supplementary Figure 47: ^{1}H NMR Spectrum of **5fa** (400 MHz, $CDCl_3$)

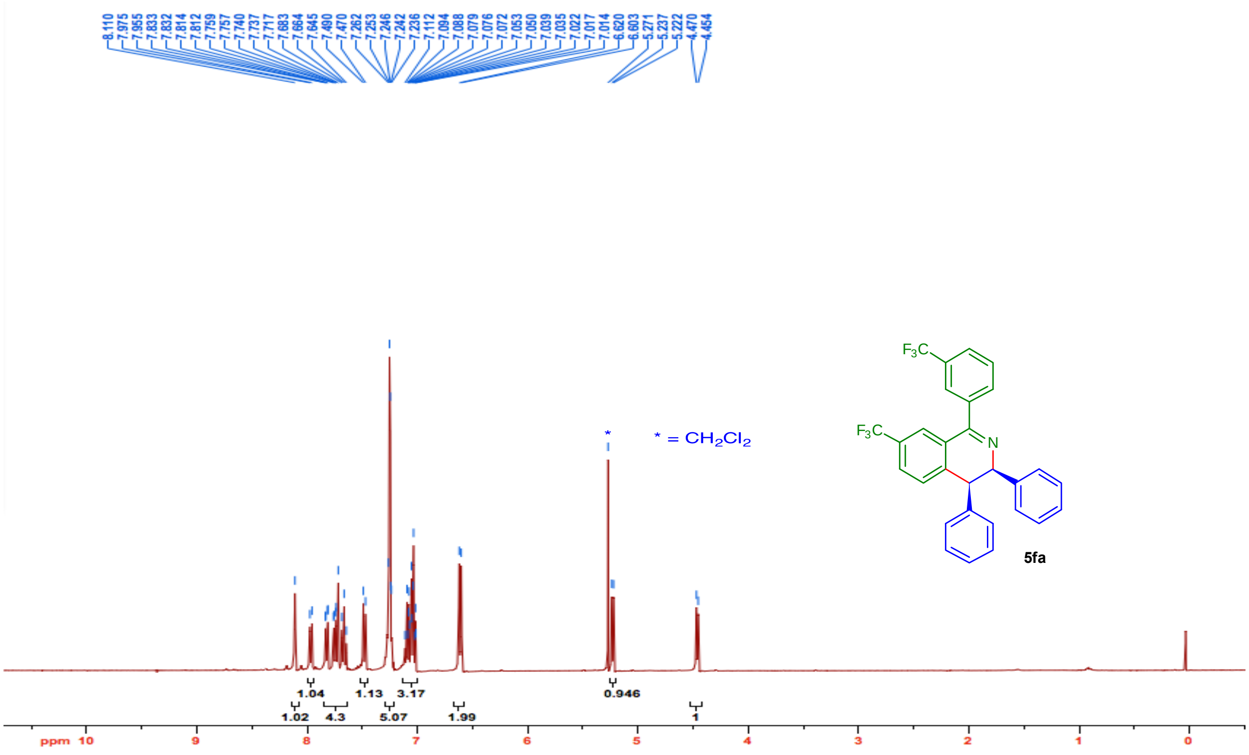

Supplementary Figure 48: ^{13}C NMR Spectrum of **5fa** (100 MHz, $CDCl_3$)

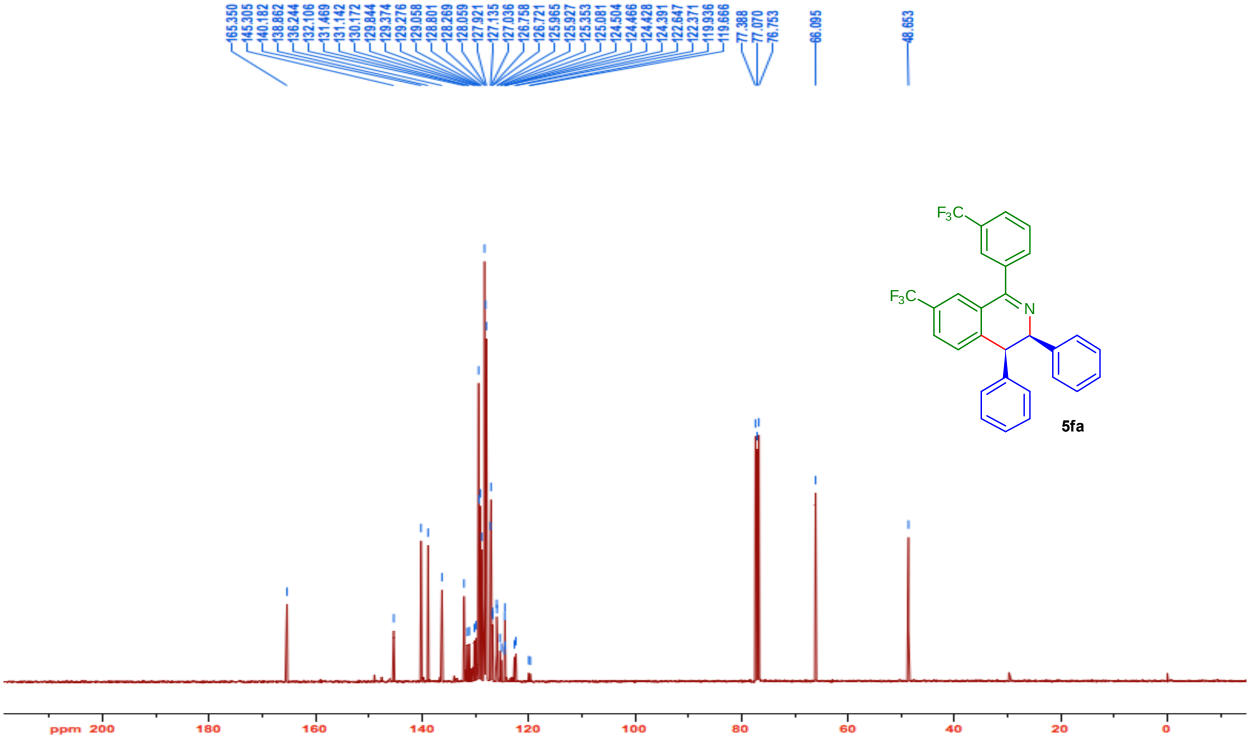

Supplementary Figure 49: ^{19}F NMR Spectrum of **5fa** (376 MHz, $CDCl_3$)

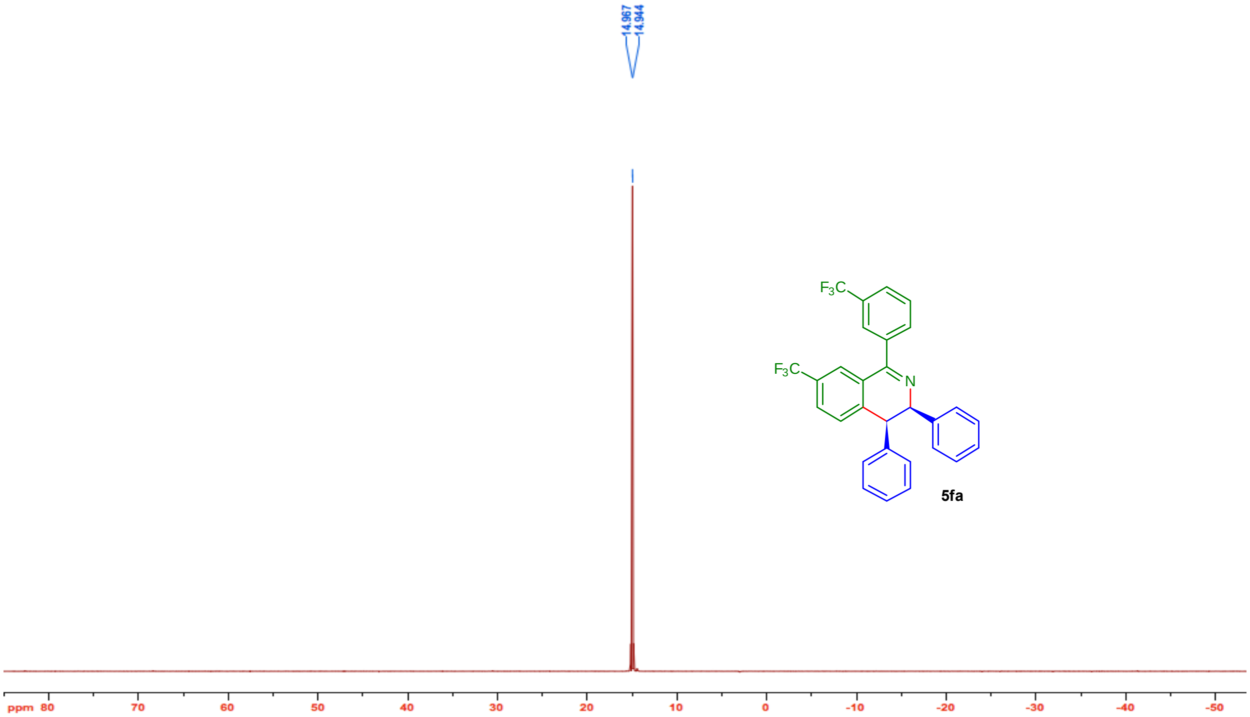

Supplementary Figure 50: ^{1}H NMR Spectrum of **5ga** (400 MHz, $CDCl_3$)

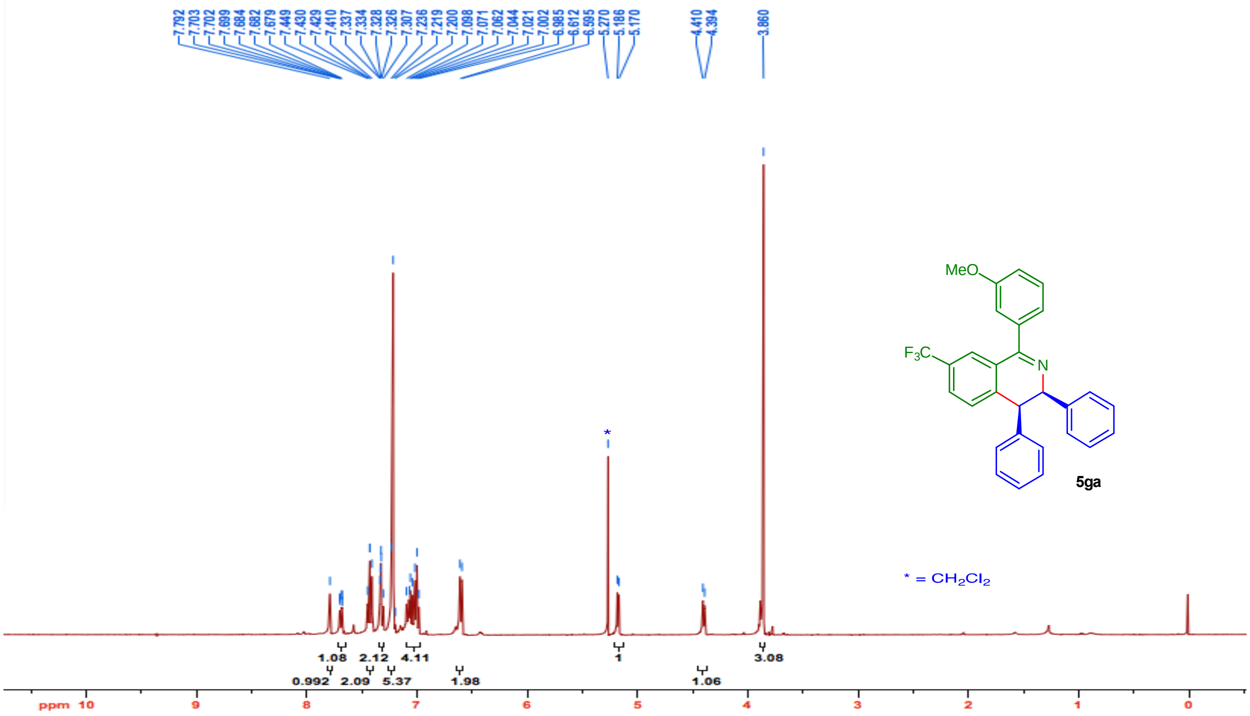

Supplementary Figure 51: ^{13}C NMR Spectrum of **5ga** (100 MHz, $CDCl_3$)

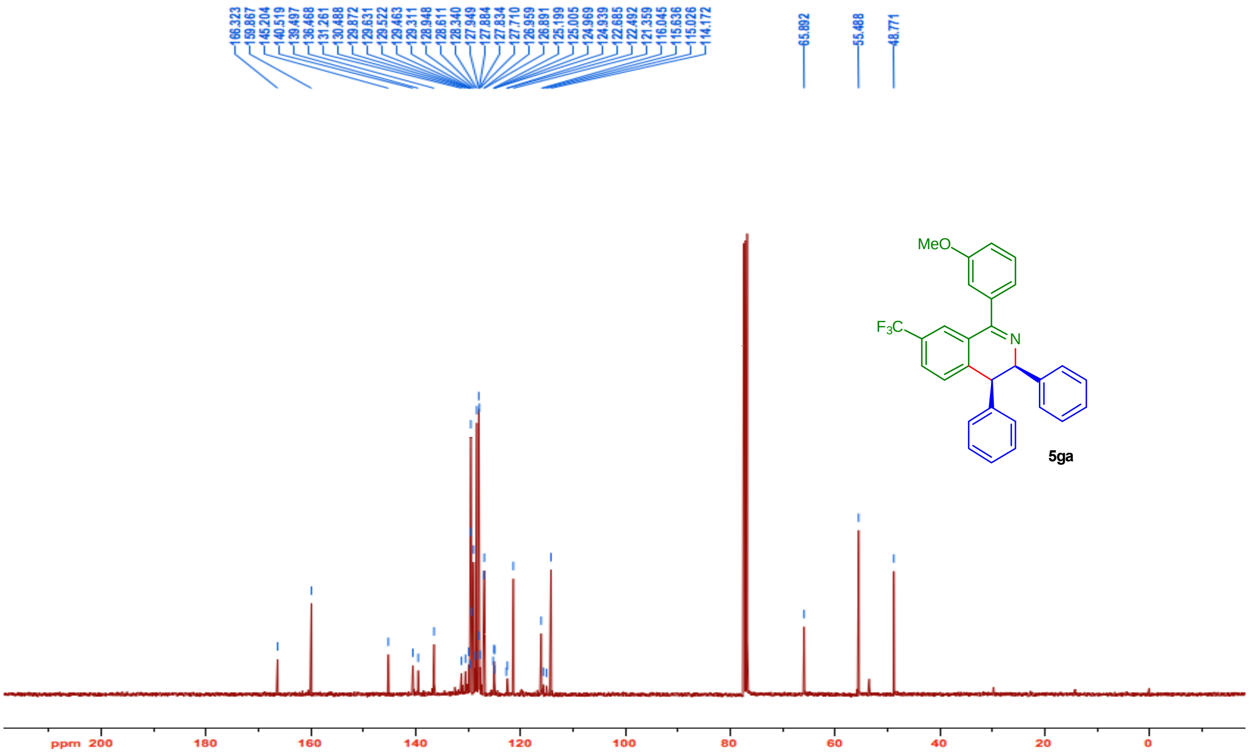

Supplementary Figure 52: ^{19}F NMR Spectrum of **5ga** (376 MHz, $CDCl_3$)

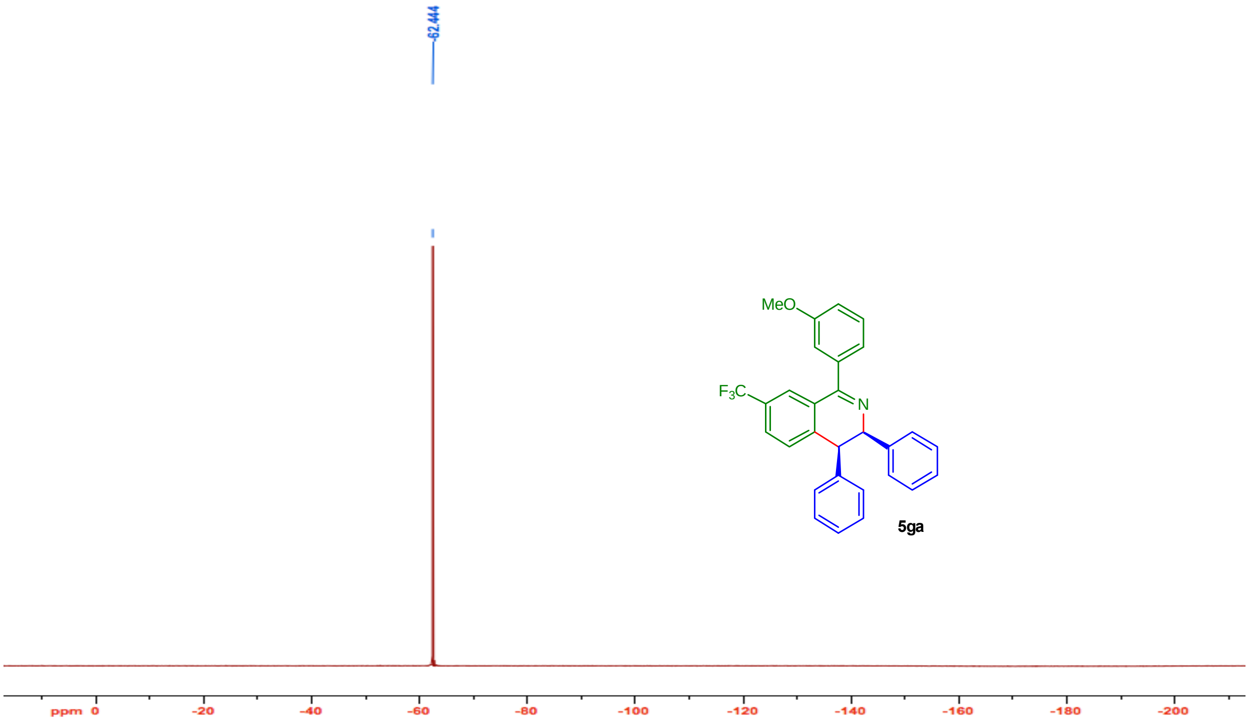

Supplementary Figure 53: 2D-NOESY Spectrum of **5ga** (400 MHz, $CDCl_3$)

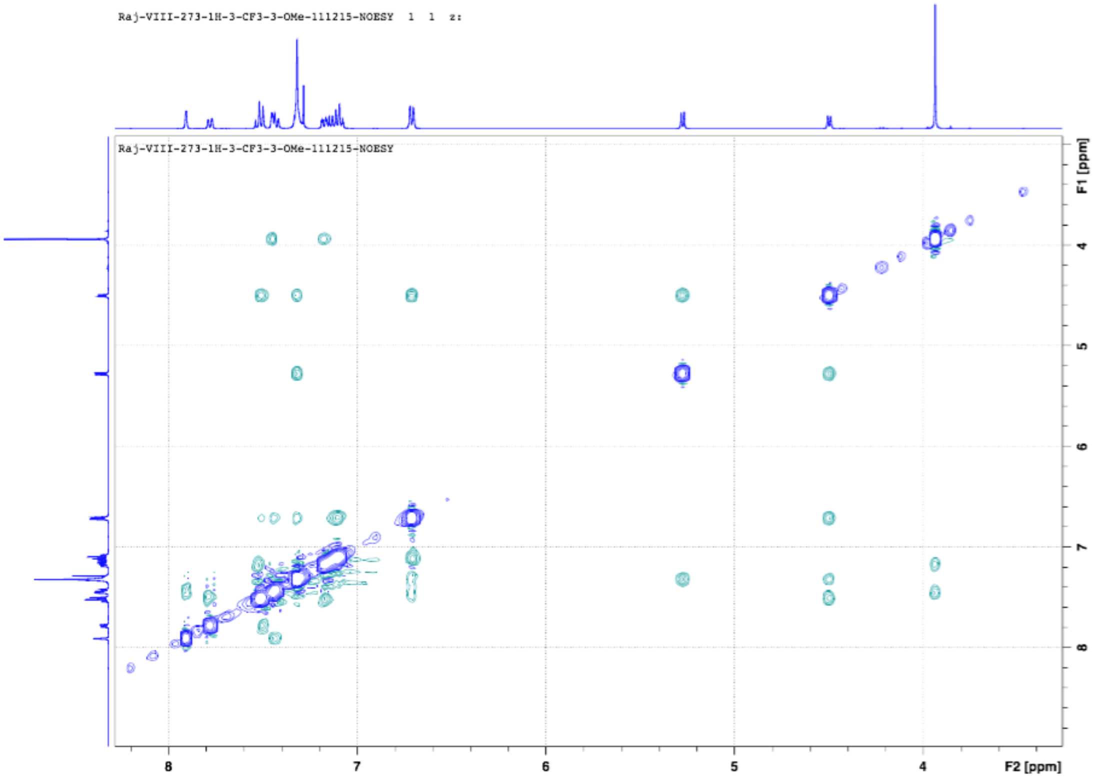

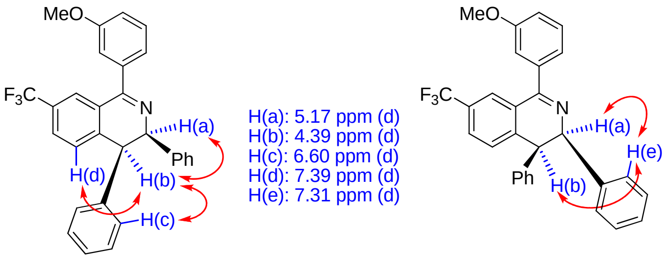

Supplementary Figure 54: ^{1}H NMR Spectrum of **5ha** (400 MHz, $CDCl_3$)

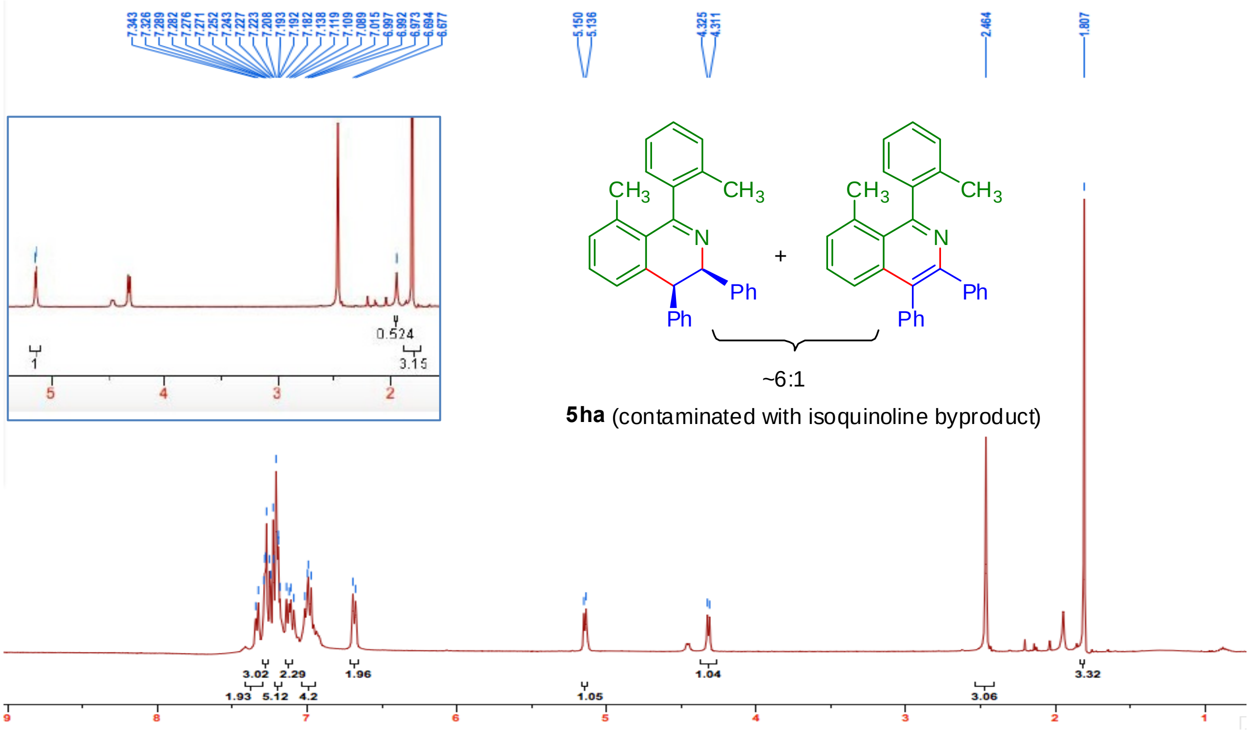

Supplementary Figure 55: ^{13}C NMR Spectrum of **5ha** (100 MHz, $CDCl_3$)

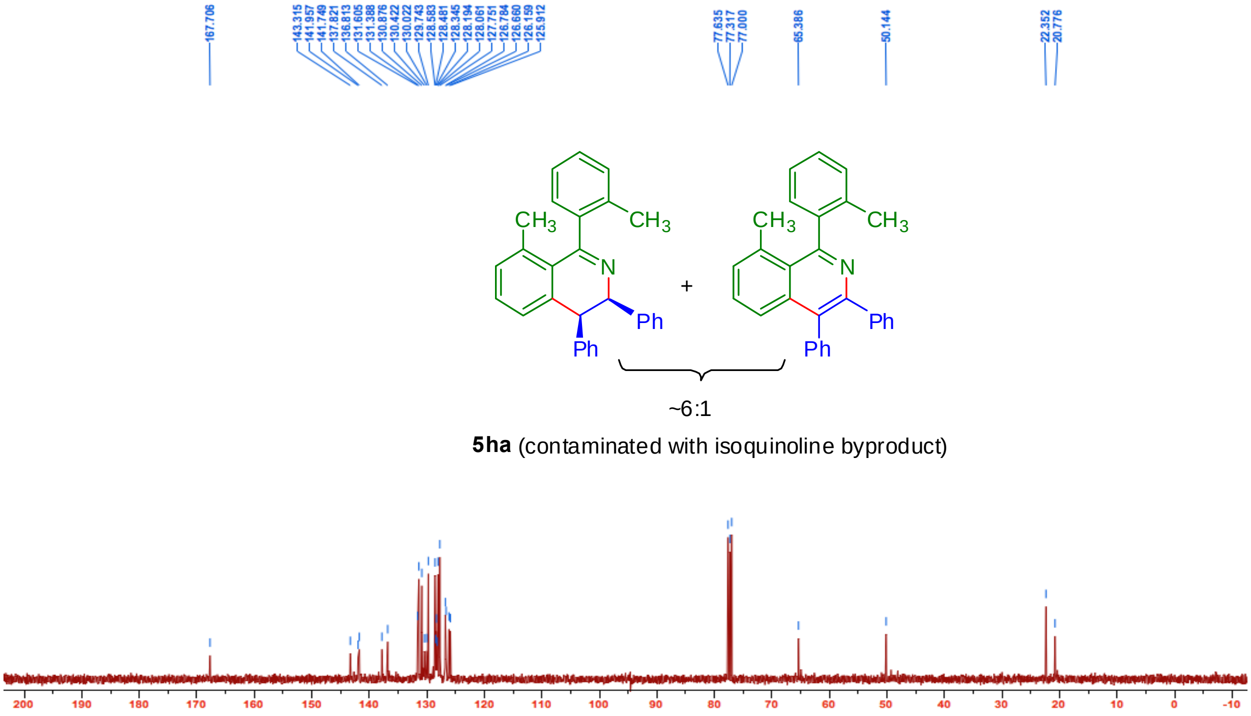

Supplementary Figure 56: ^{1}H NMR Spectrum of **5ia** (400 MHz, $CDCl_3$)

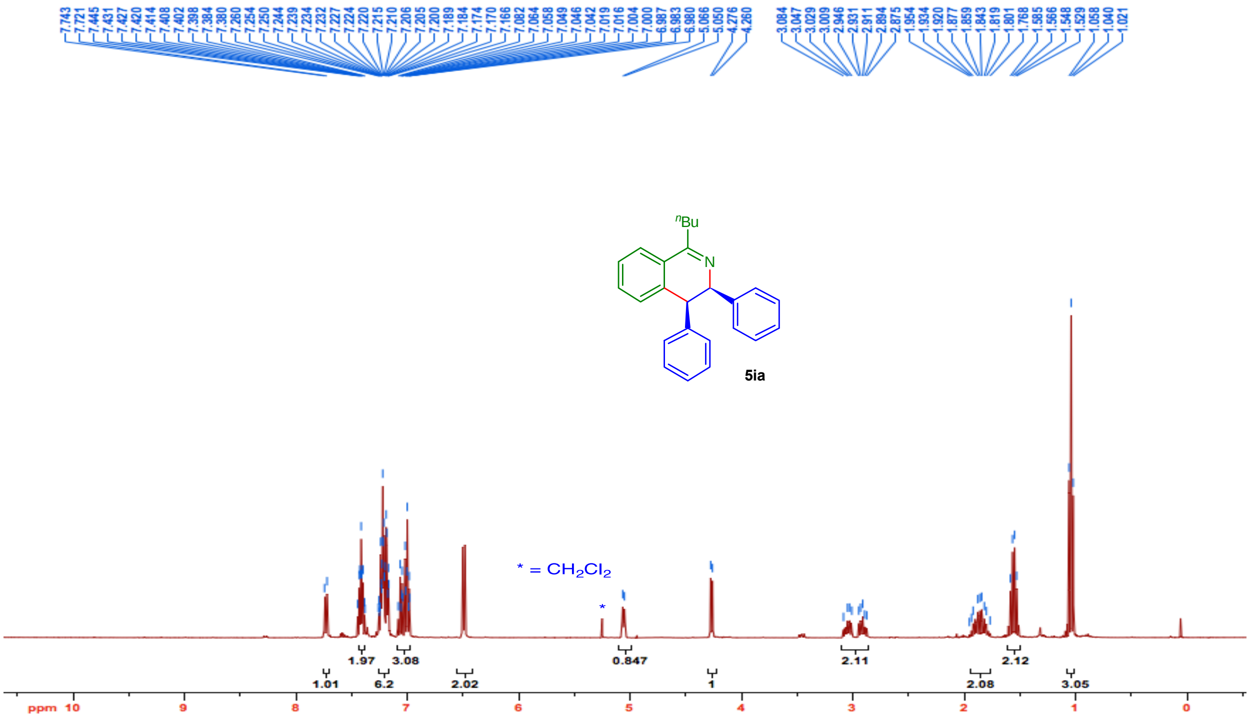

Supplementary Figure 57: ^{13}C NMR Spectrum of **5ia** (100 MHz, $CDCl_3$)

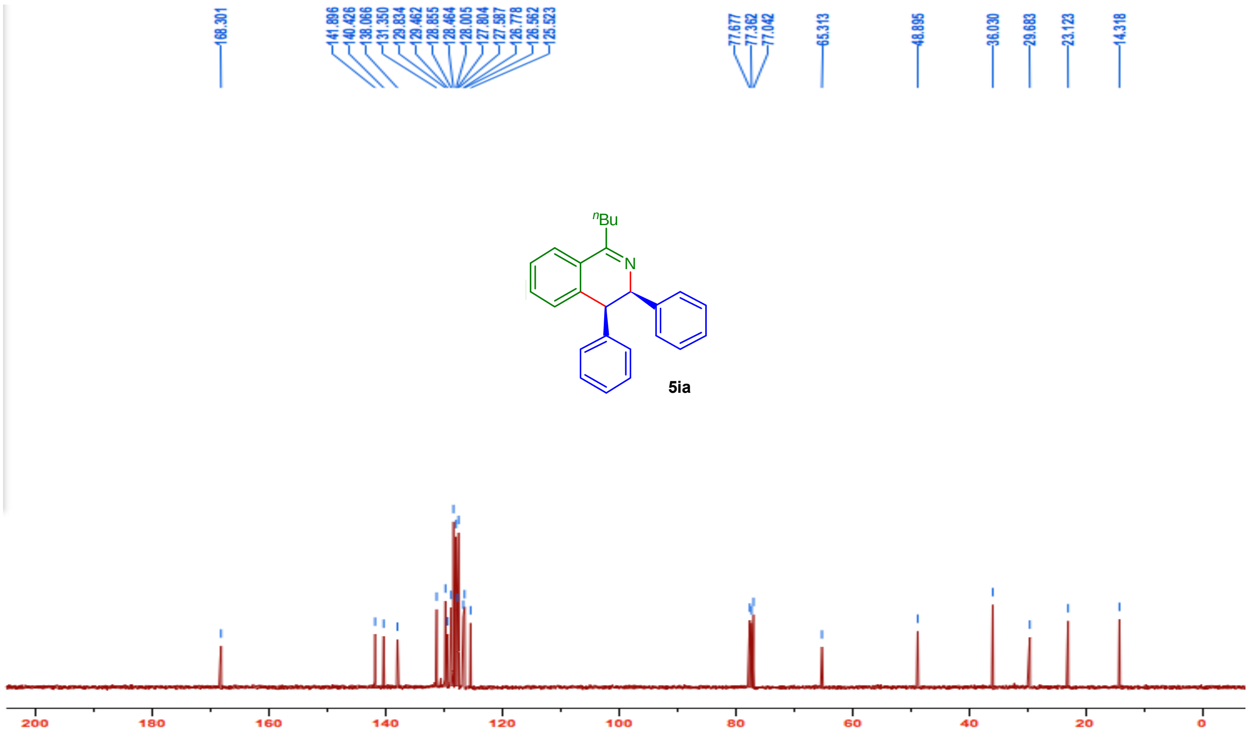

Supplementary Figure 58: ^{1}H NMR Spectrum of **5ja** (400 MHz, $CDCl_3$)

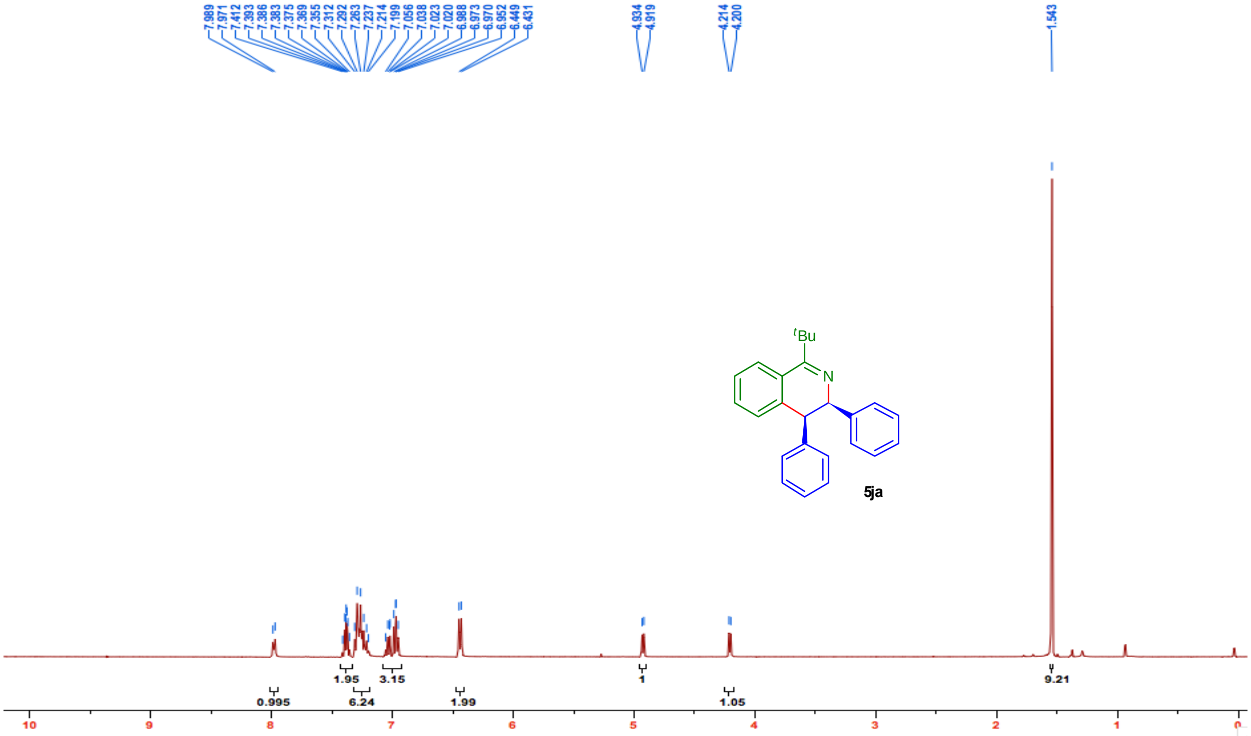

Supplementary Figure 59: ^{13}C NMR Spectrum of **5ja** (100 MHz, $CDCl_3$)

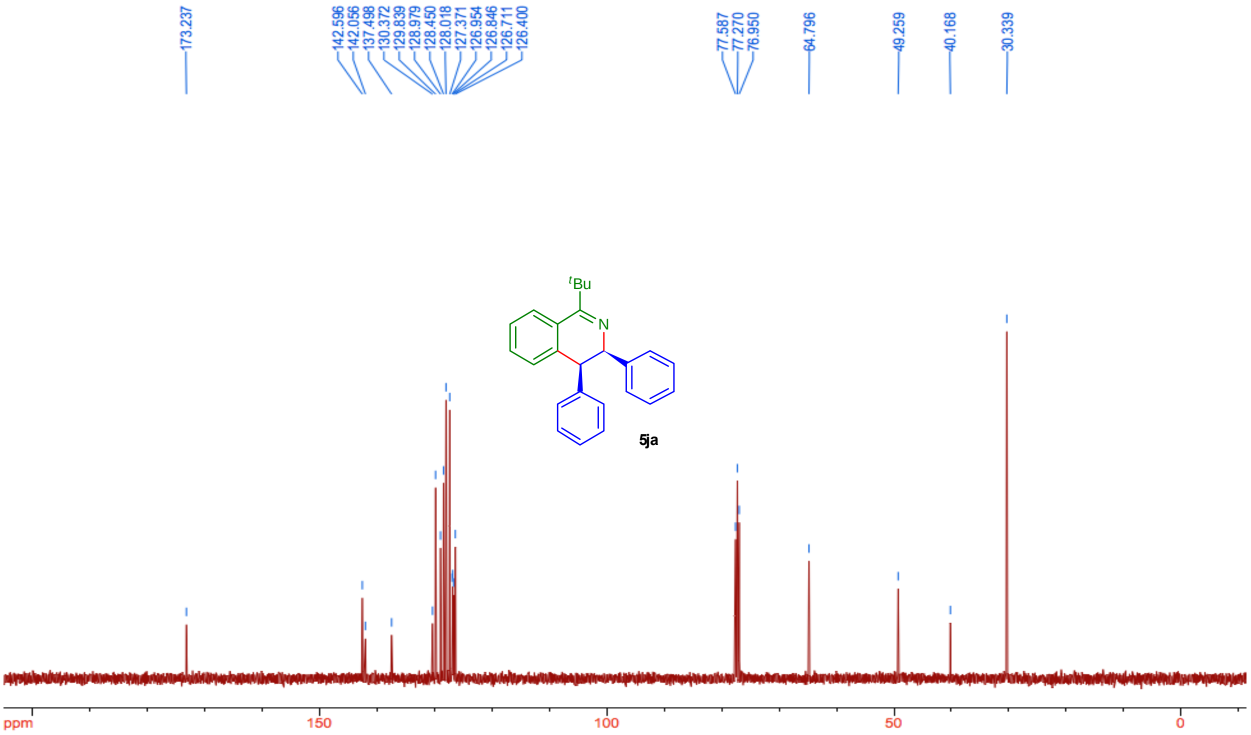

Supplementary Figure 60: ^{1}H NMR Spectrum of **5ka** (400 MHz, $CDCl_3$)

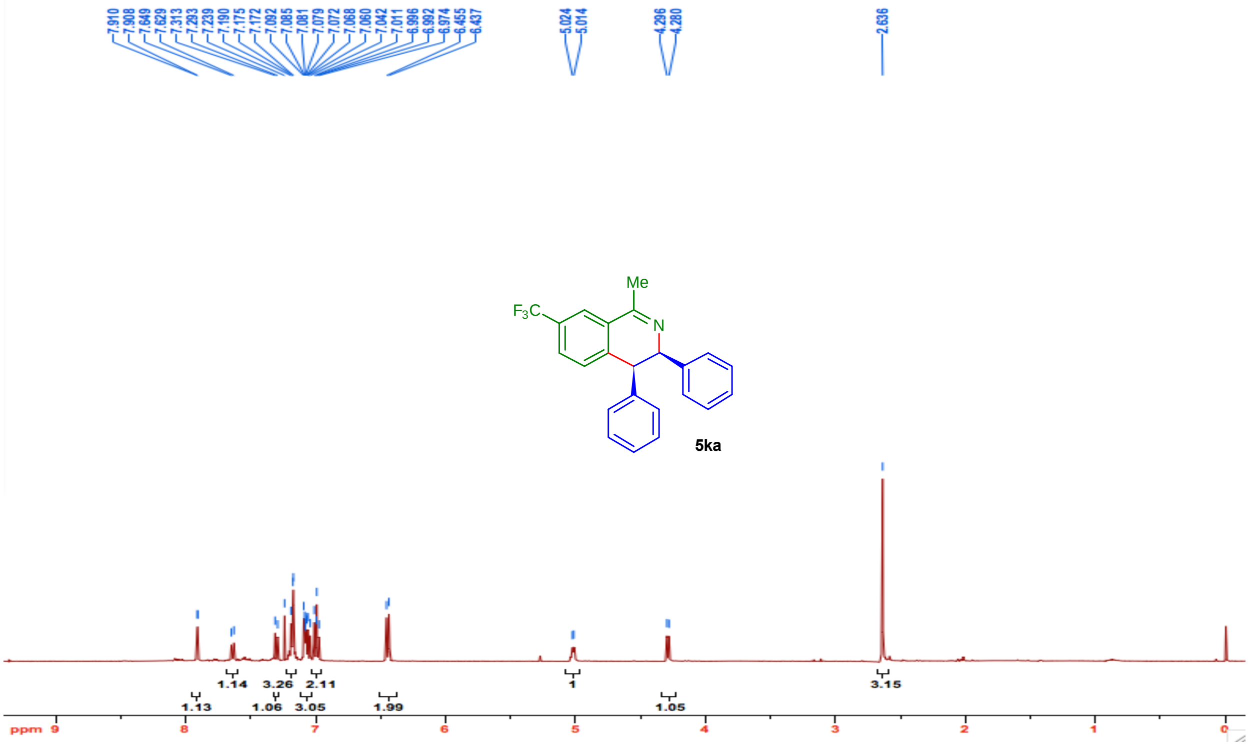

Supplementary Figure 61: ^{13}C NMR Spectrum of **5ka** (100 MHz, $CDCl_3$)

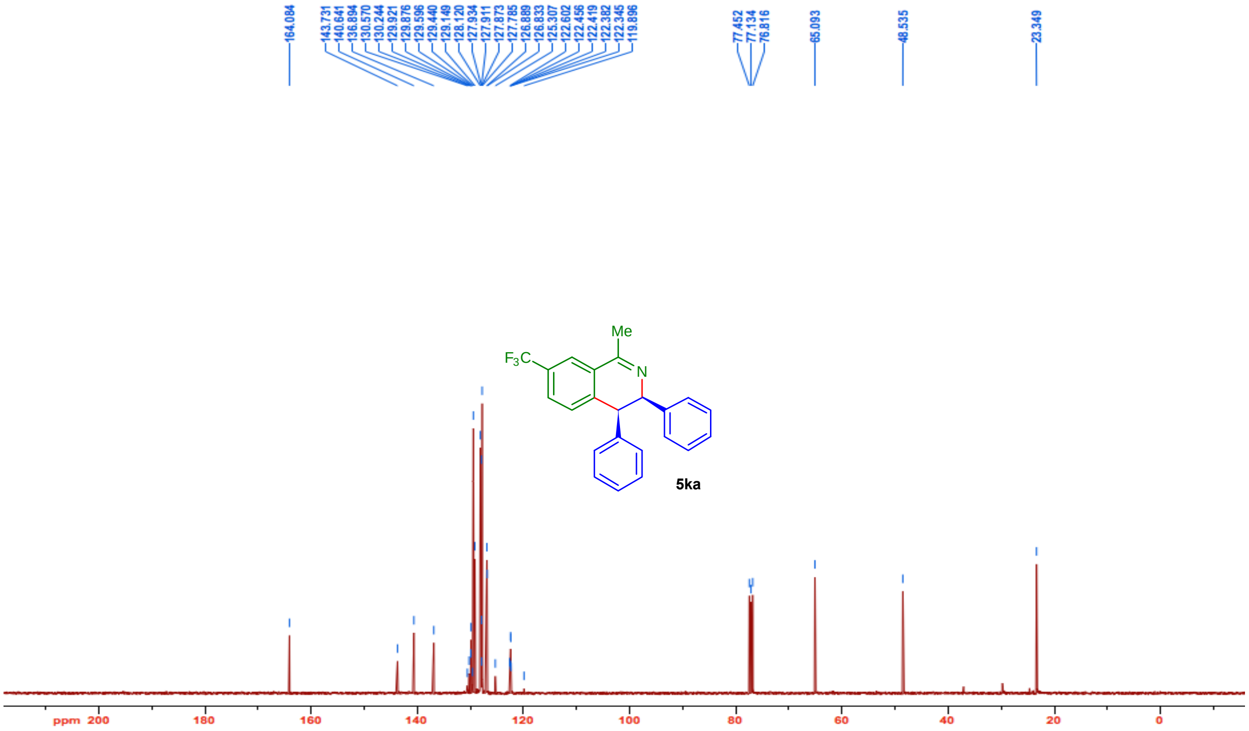

Supplementary Figure 62: ^{19}F NMR Spectrum of **5ka** (376 MHz, $CDCl_3$)

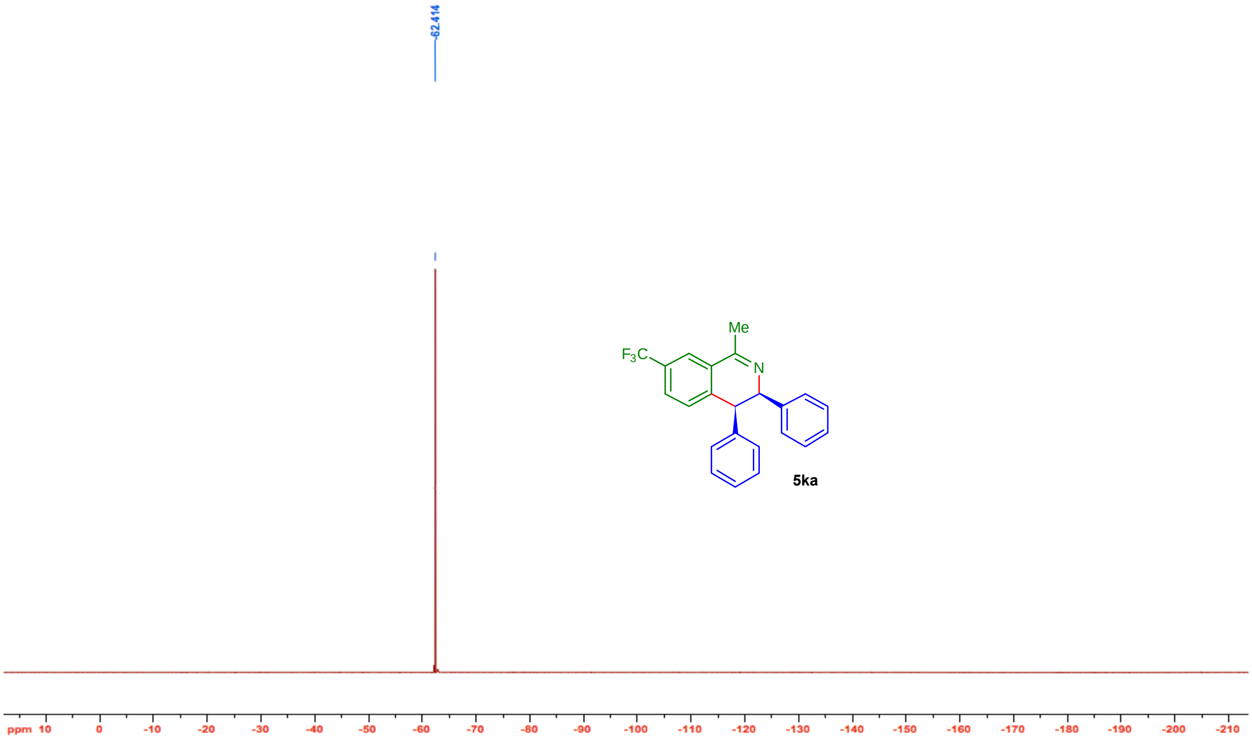

Supplementary Figure 63: ^{1}H NMR Spectrum of **5la** (400 MHz, $CDCl_3$)

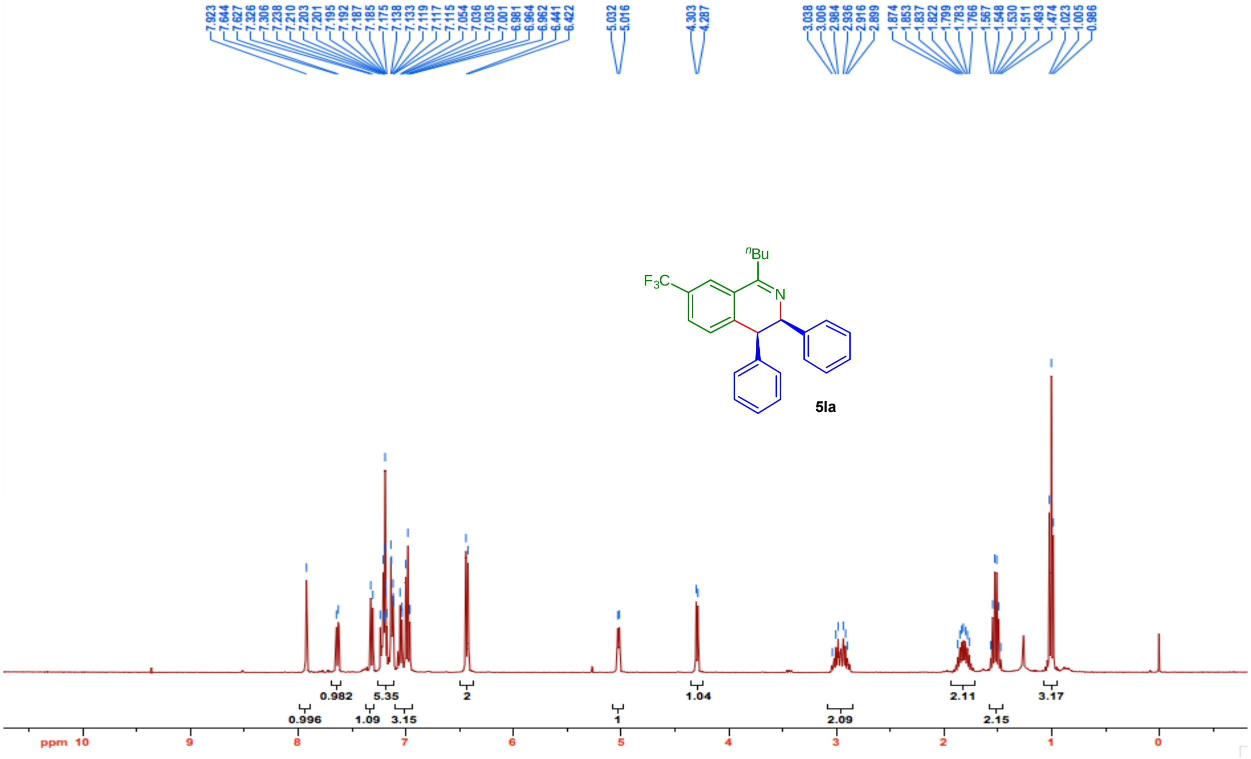

Supplementary Figure 64: ^{13}C NMR Spectrum of **5la** (100 MHz, $CDCl_3$)

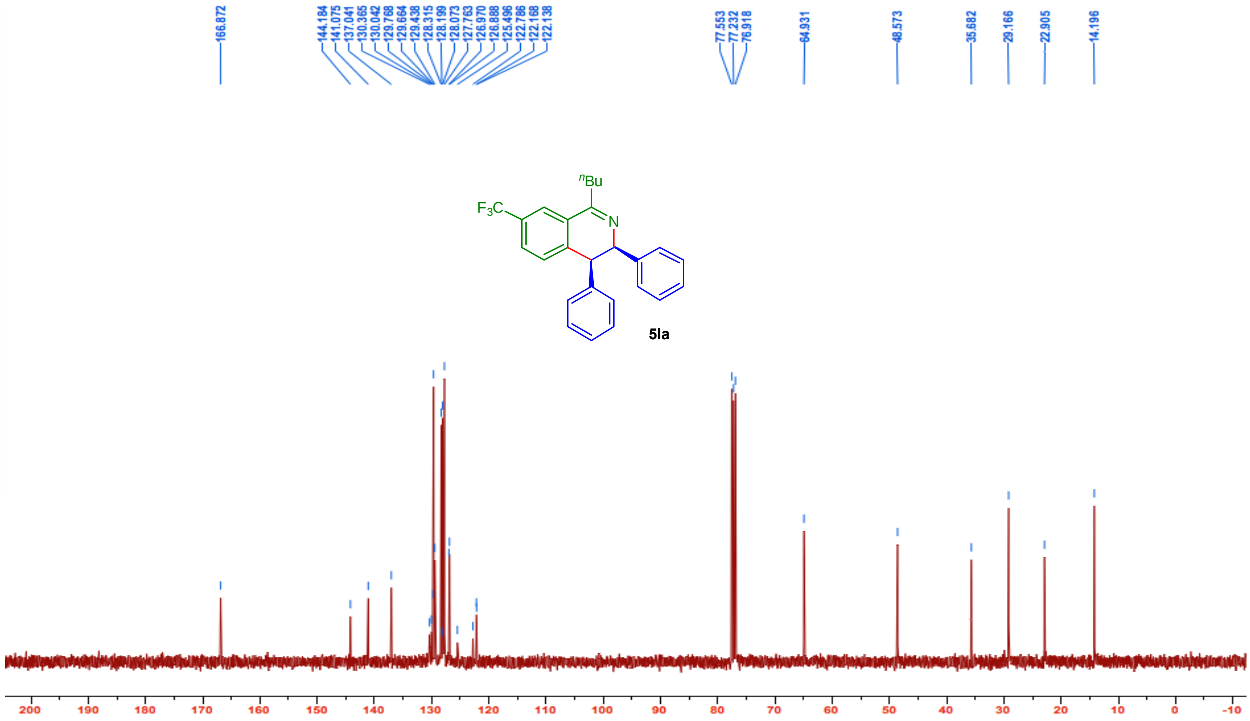

Supplementary Figure 65: ^{19}F NMR Spectrum of **5la** (376 MHz, $CDCl_3$)

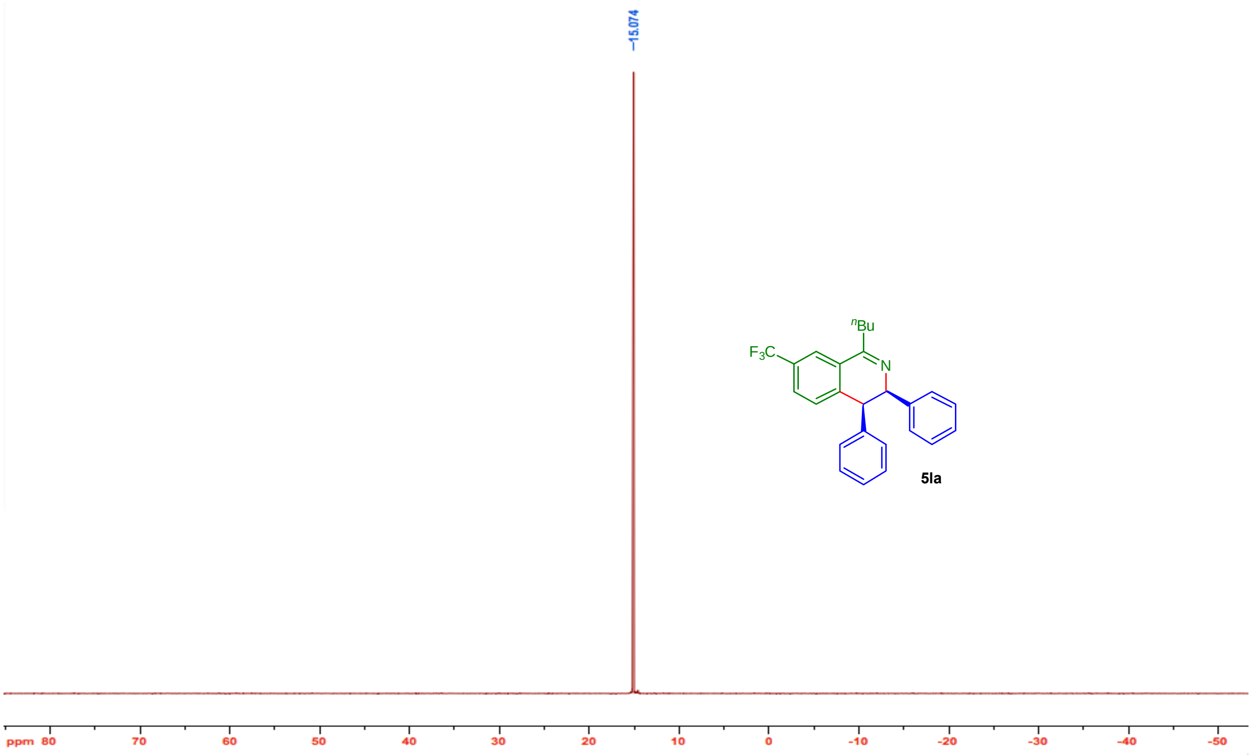

Supplementary Figure 66: ^{1}H NMR Spectrum of **5ma** (400 MHz, $CDCl_3$)

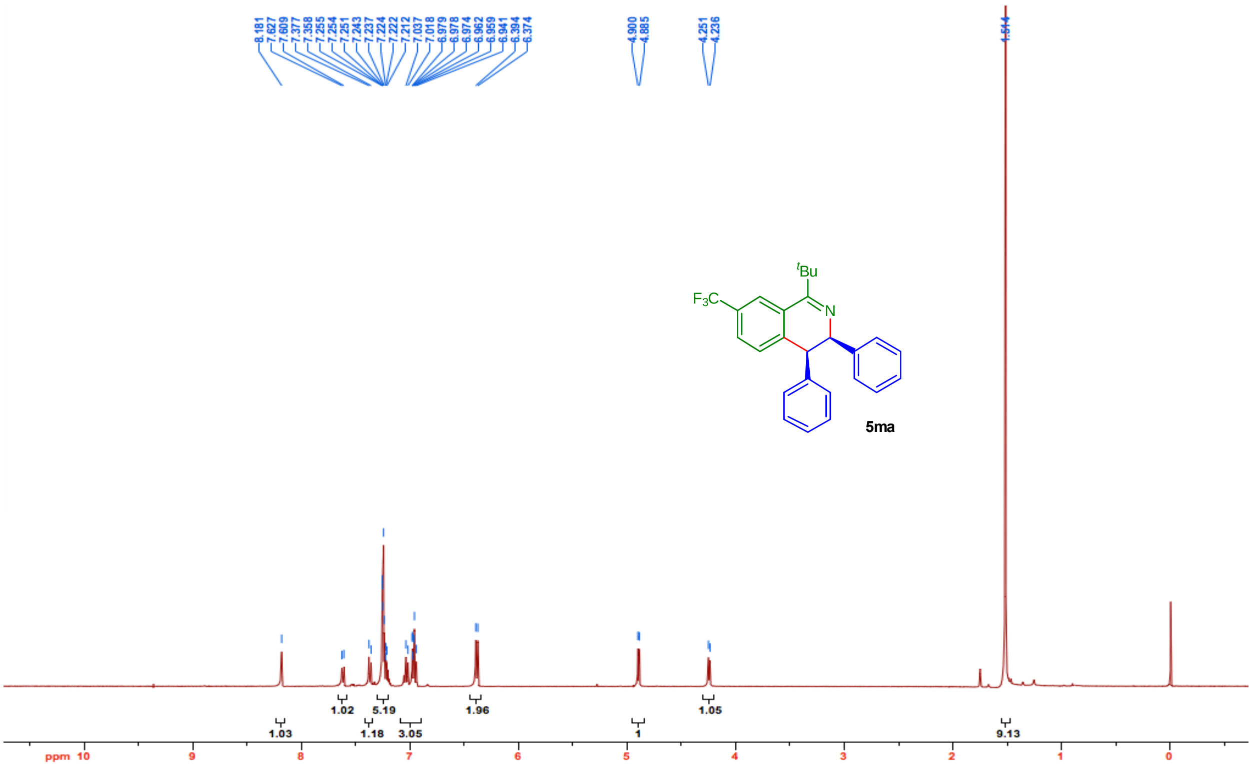

Supplementary Figure 67: ^{13}C NMR Spectrum of **5ma** (100 MHz, $CDCl_3$)

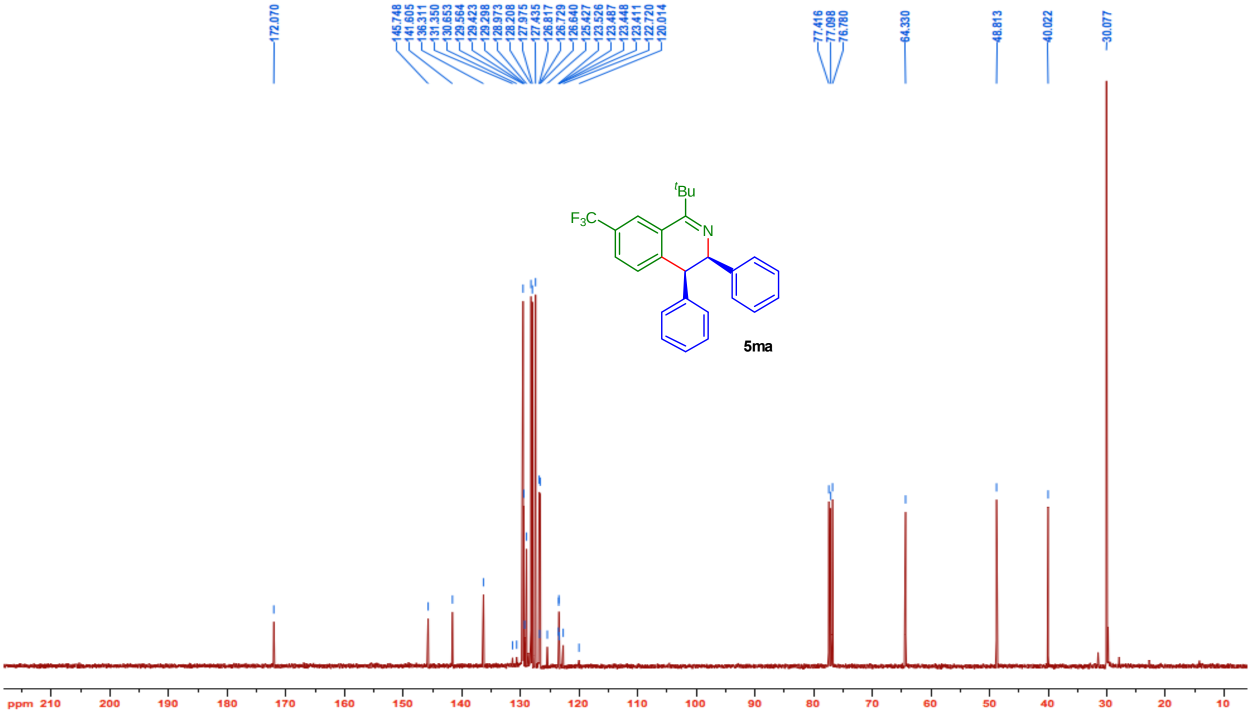

Supplementary Figure 68: ^{19}F NMR Spectrum of **5ma** (376 MHz, $CDCl_3$)

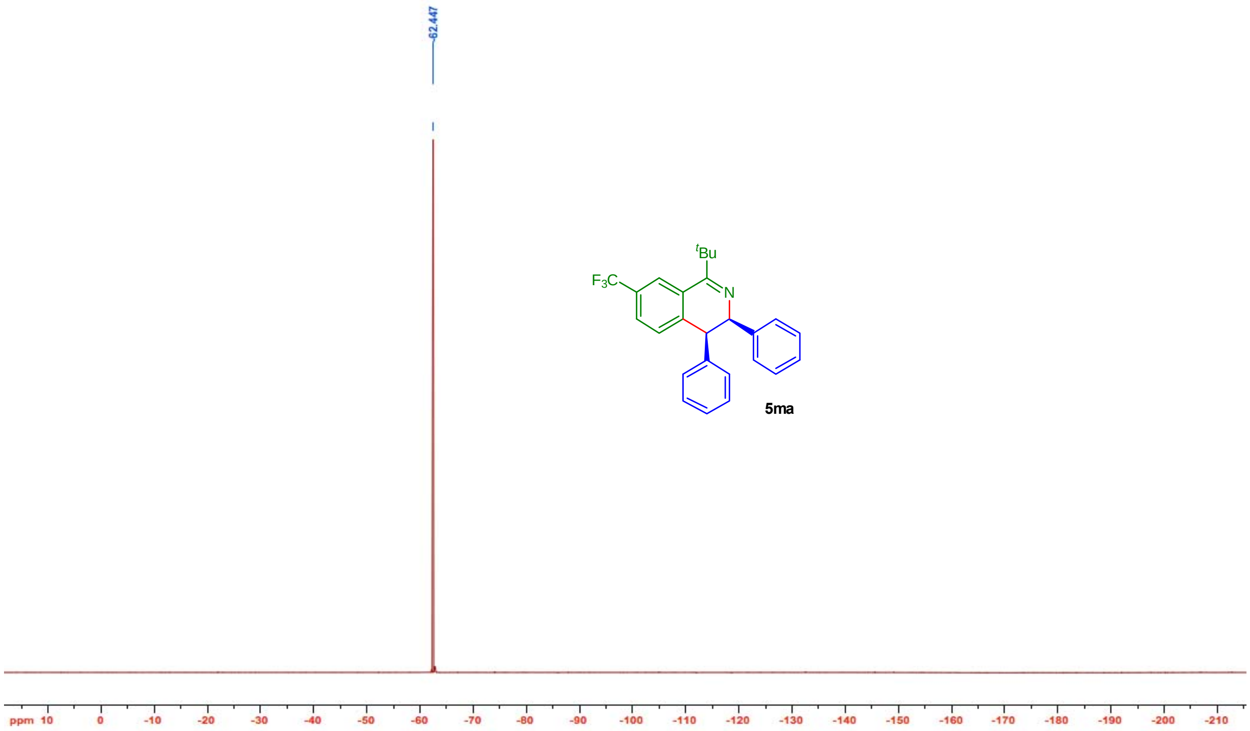

Supplementary Figure 69: ^{1}H NMR Spectrum of **5na** (400 MHz, $CDCl_3$)

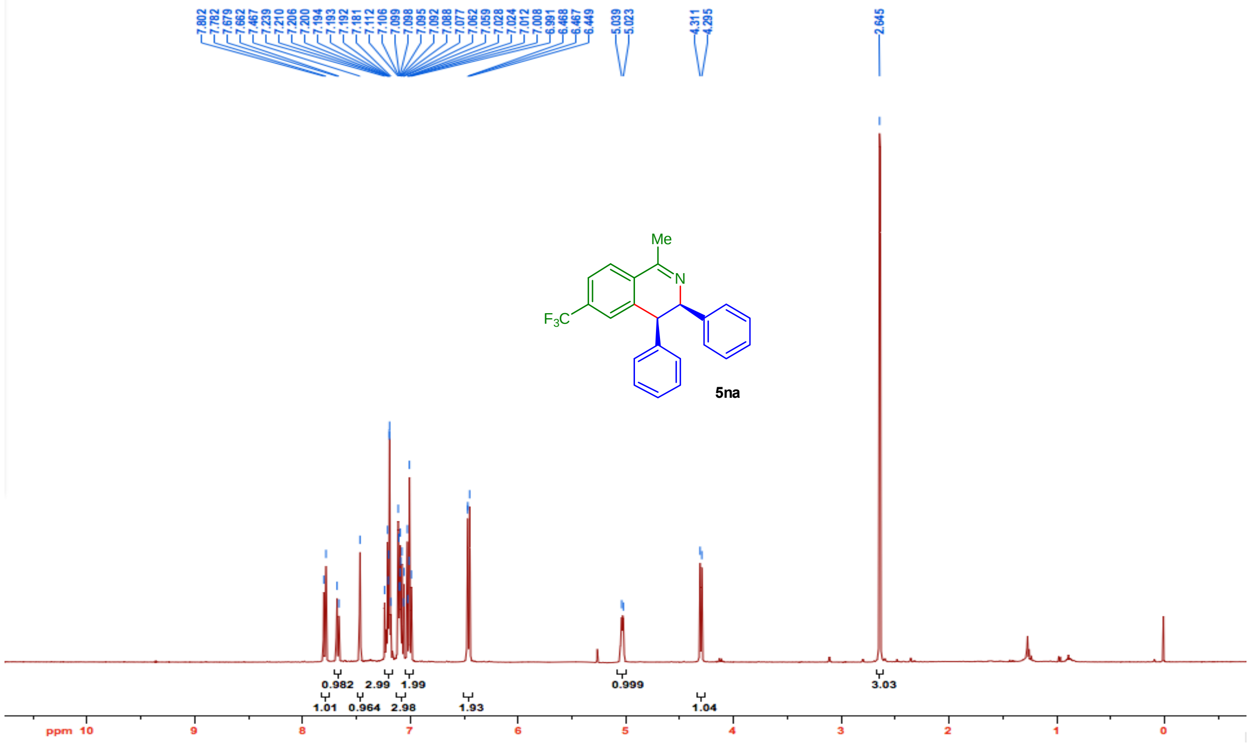

Supplementary Figure 70: ^{13}C NMR Spectrum of **5na** (100 MHz, $CDCl_3$)

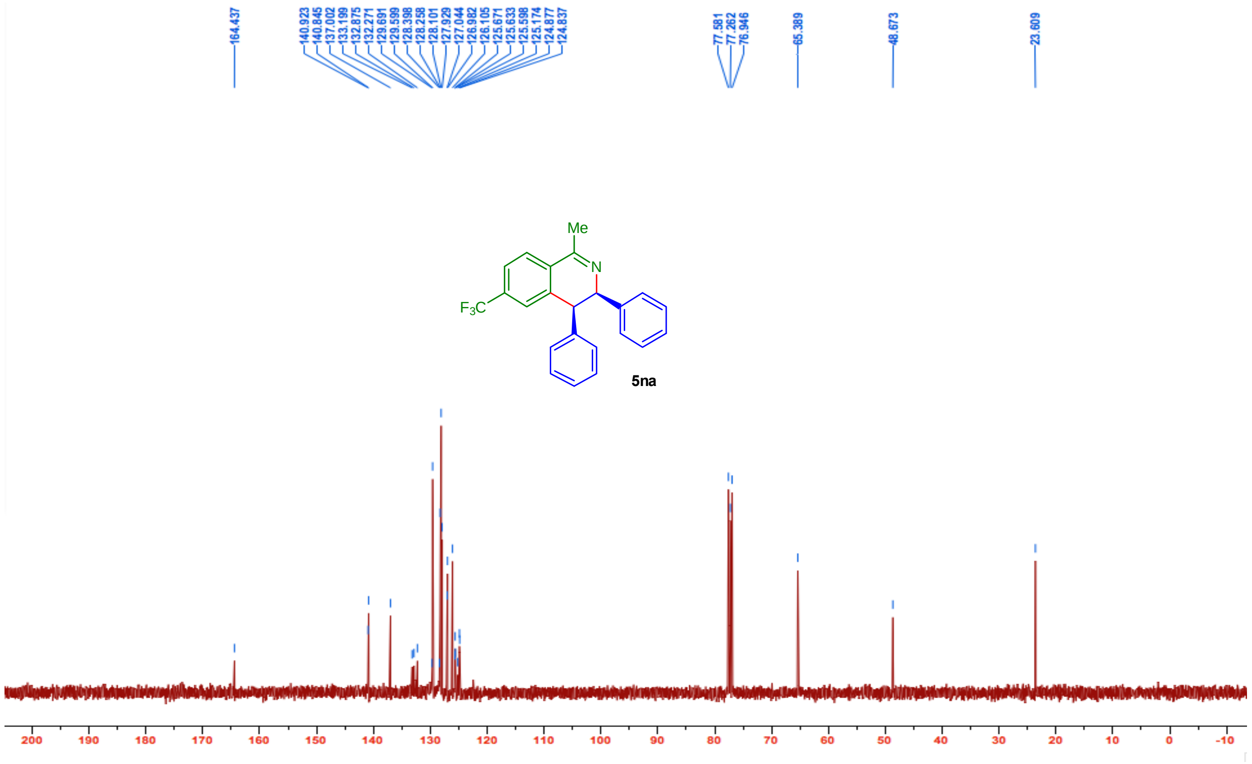

Supplementary Figure 71: ^{19}F NMR Spectrum of **5na** (376 MHz, $CDCl_3$)

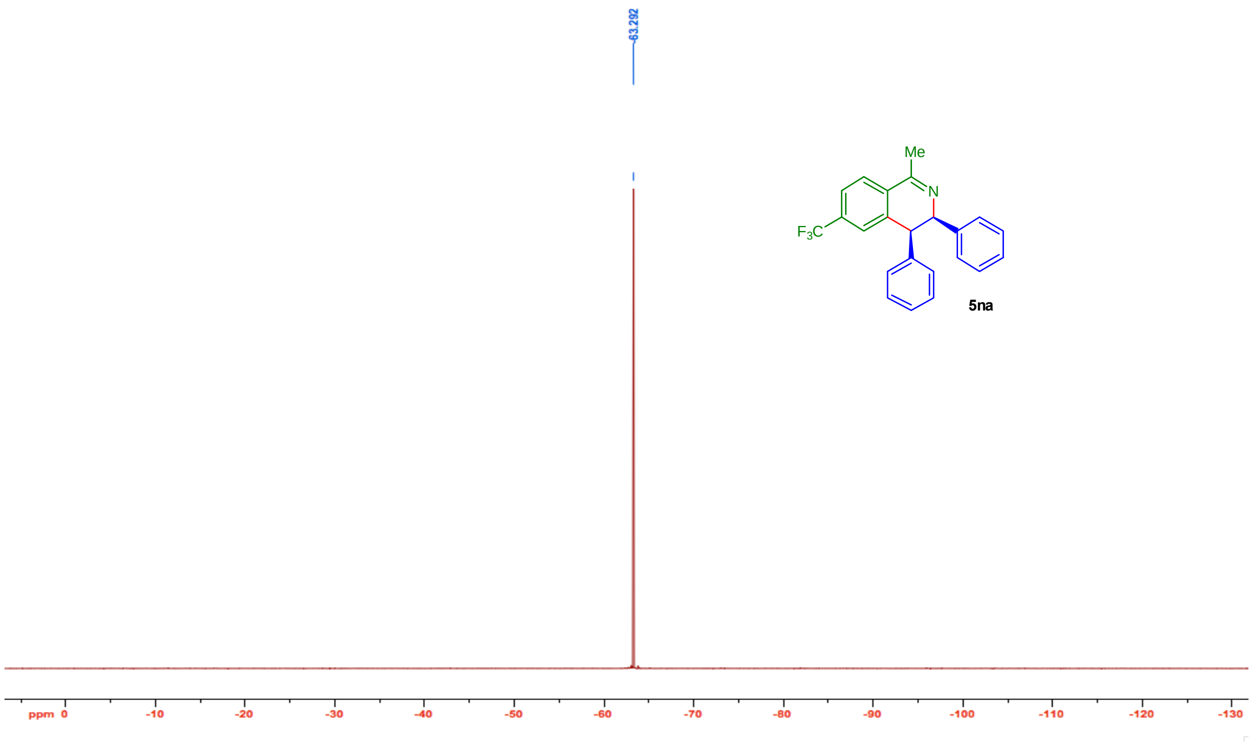

Supplementary Figure 72: ^{1}H NMR Spectrum of **5oa** (400 MHz, $CDCl_3$)

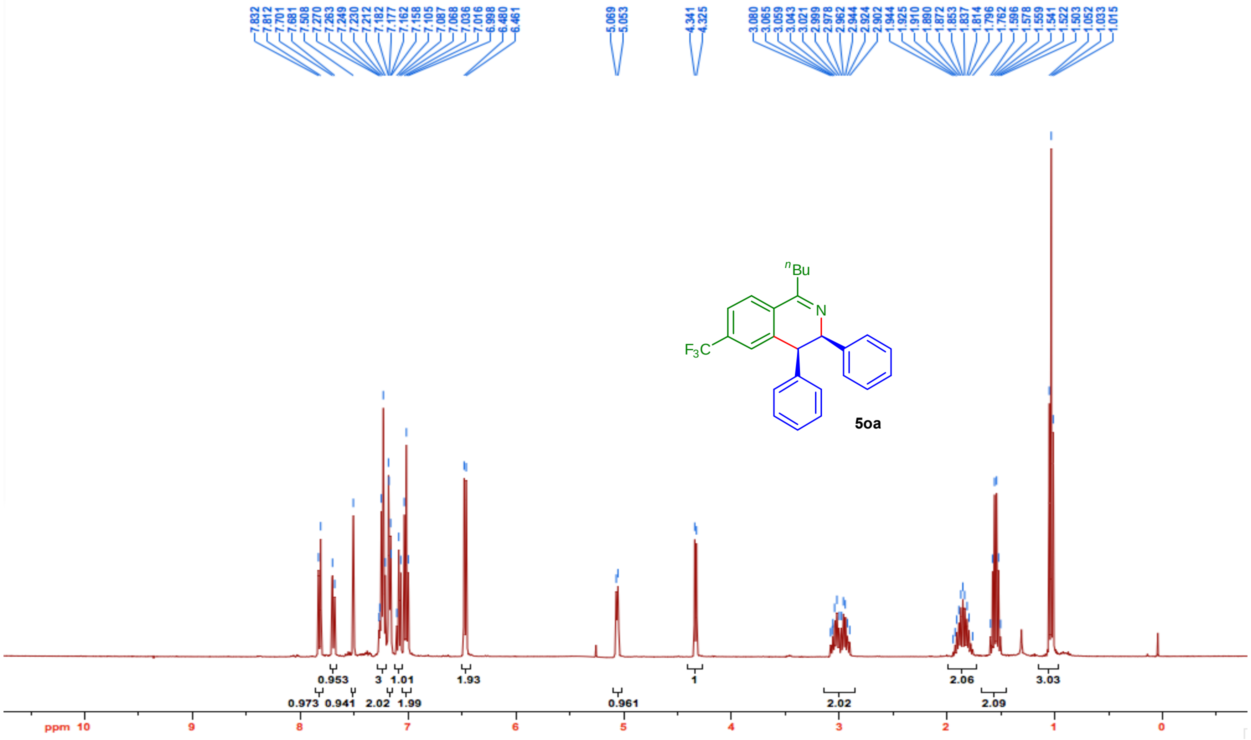

Supplementary Figure 73: ^{13}C NMR Spectrum of **5oa** (100 MHz, $CDCl_3$)

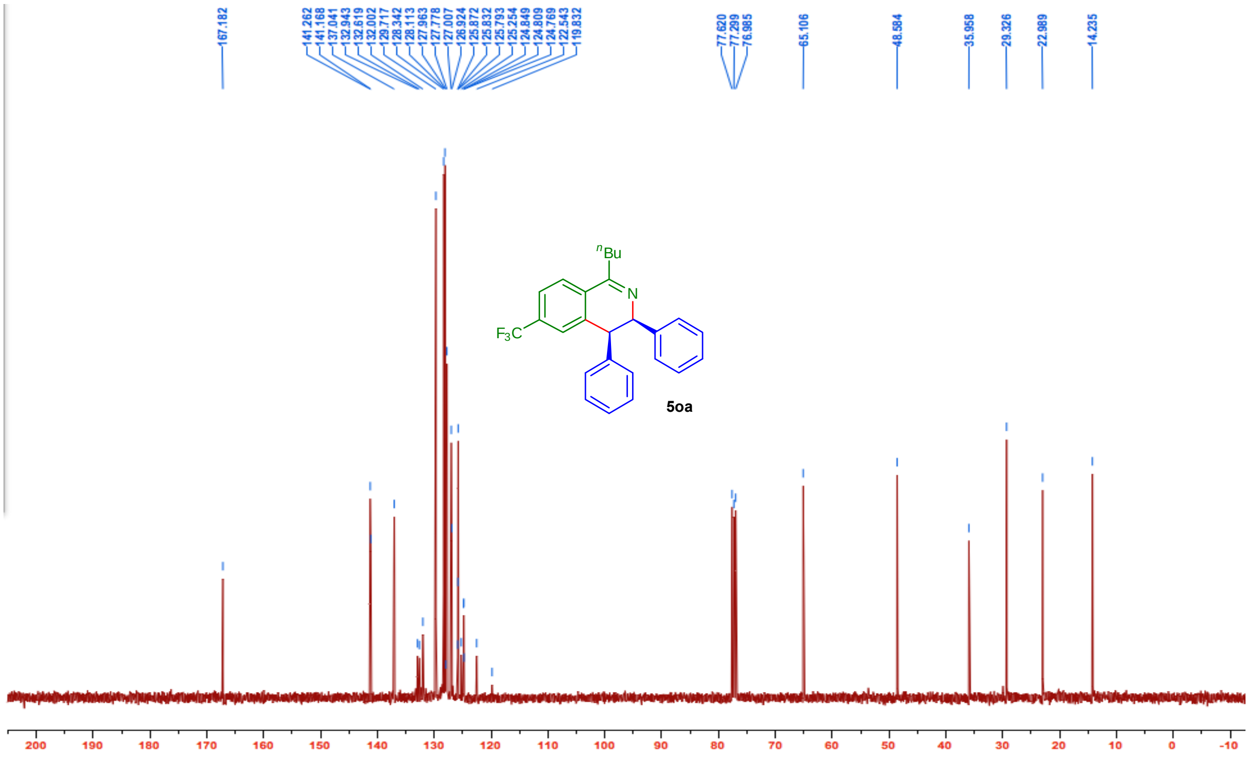

Supplementary Figure 74: ^{19}F NMR Spectrum of **5oa** (376 MHz, $CDCl_3$)

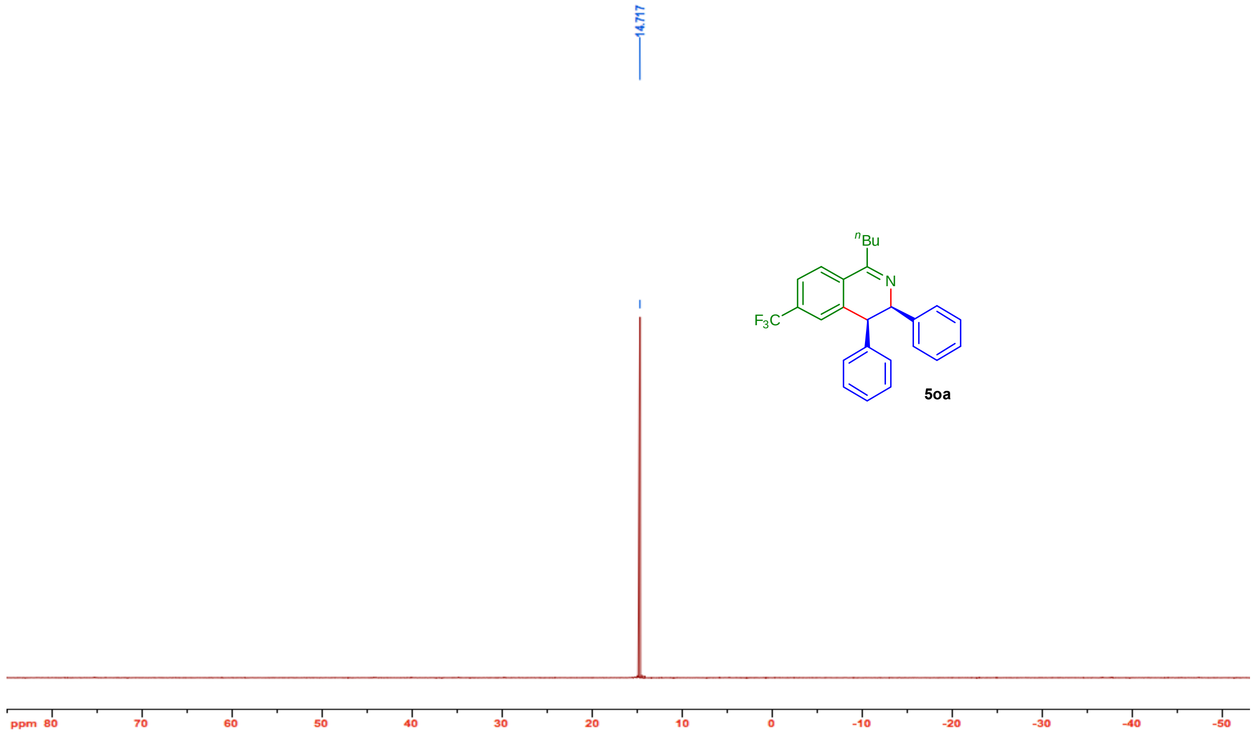

Supplementary Figure 75: ^{1}H NMR Spectrum of **8** (400 MHz, $CDCl_3$)

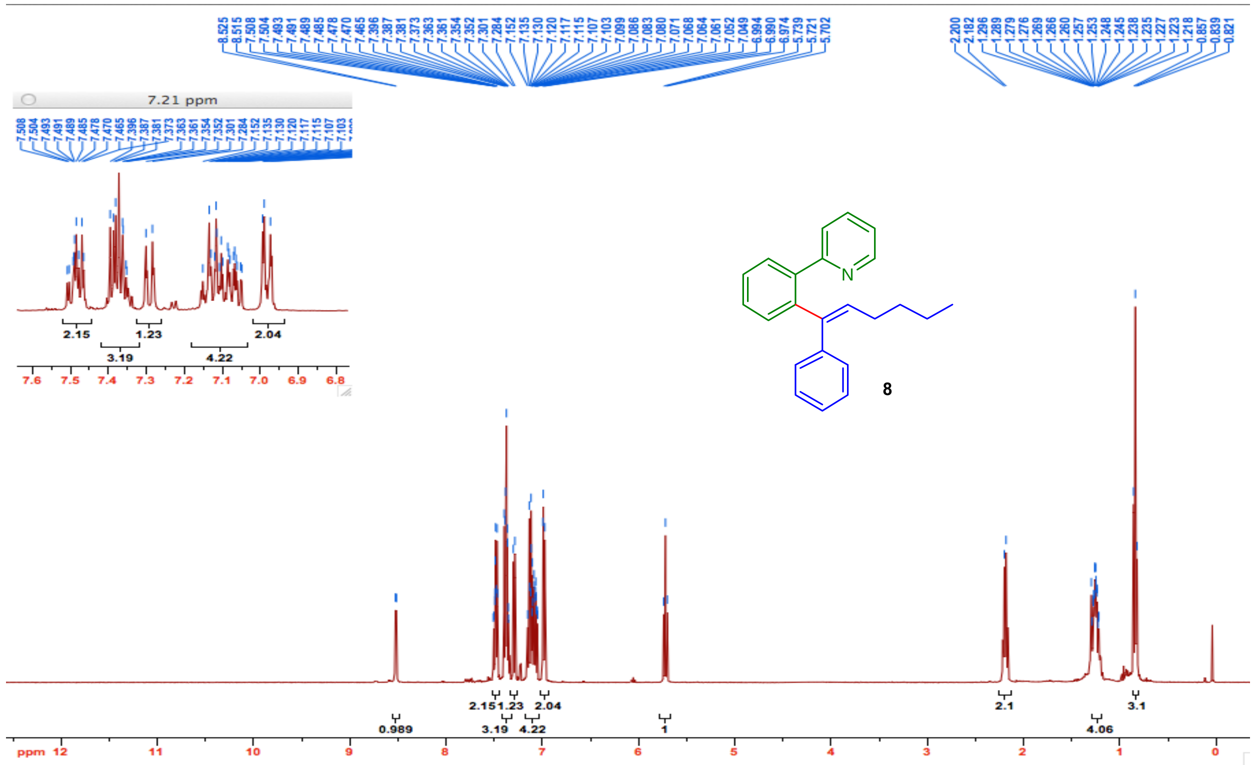

Supplementary Figure 76: ^{13}C NMR Spectrum of **8** (100 MHz, $CDCl_3$)

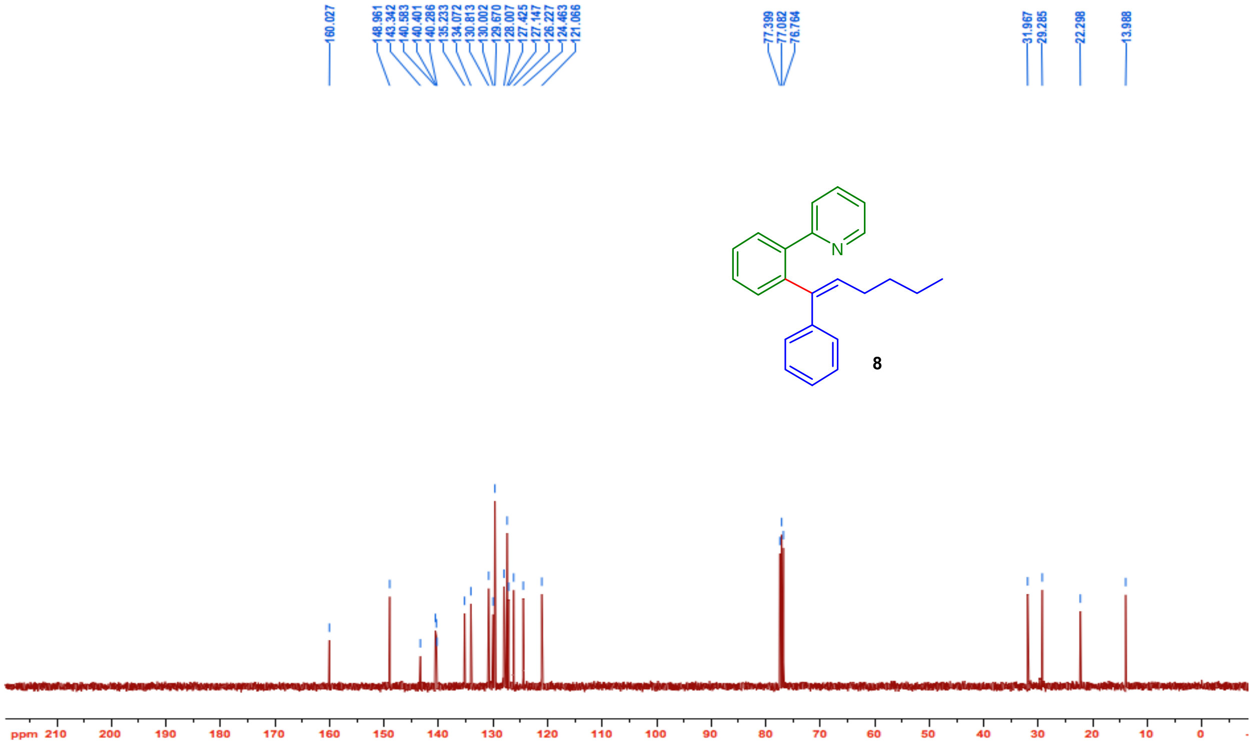

Supplementary Figure 77: 2D-NOESY Spectrum of **8** (400 MHz, $CDCl_3$)

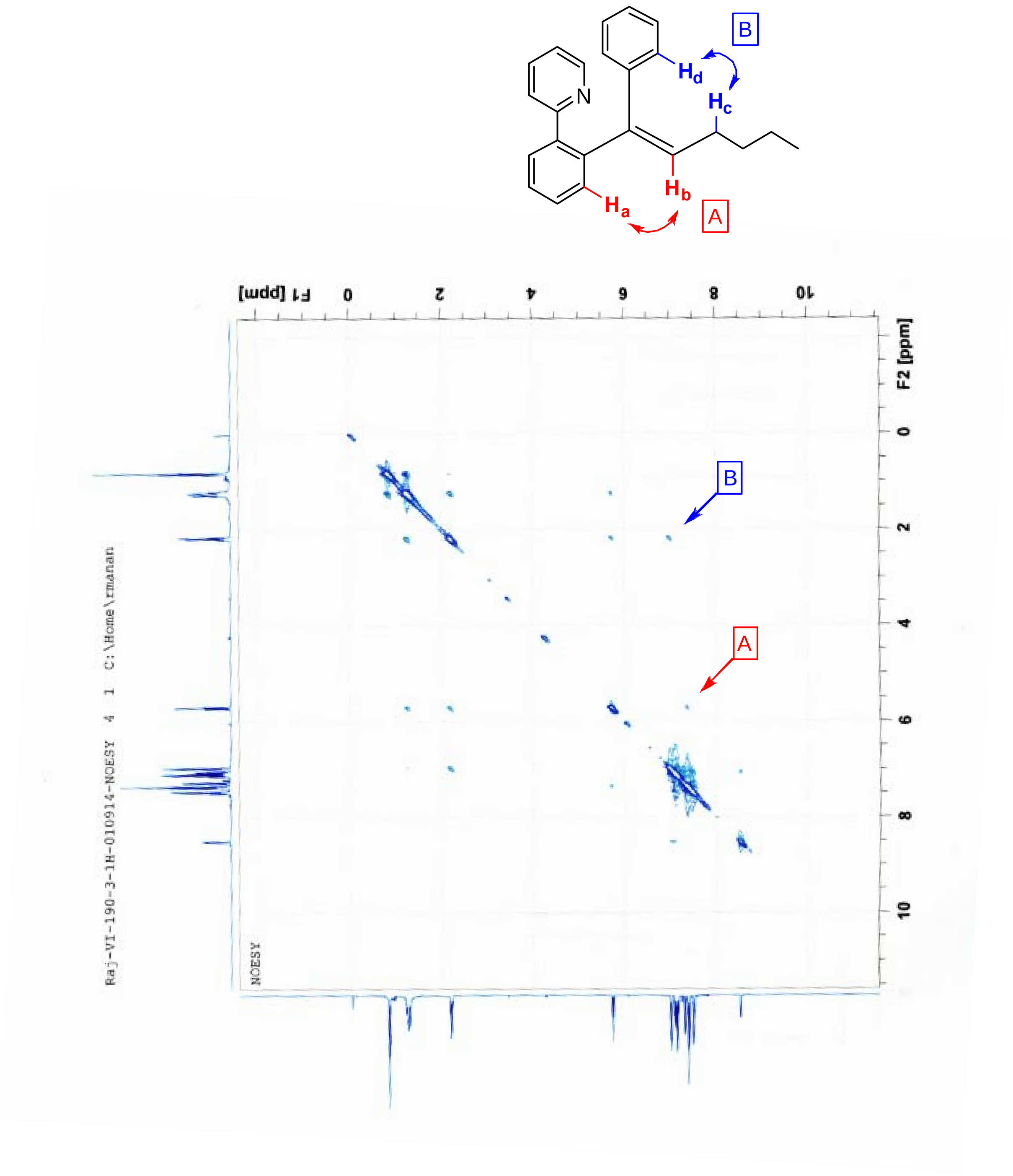

Supplementary Figure 78: ^{1}H NMR Spectrum of **9a** (400 MHz, $CDCl_3$)

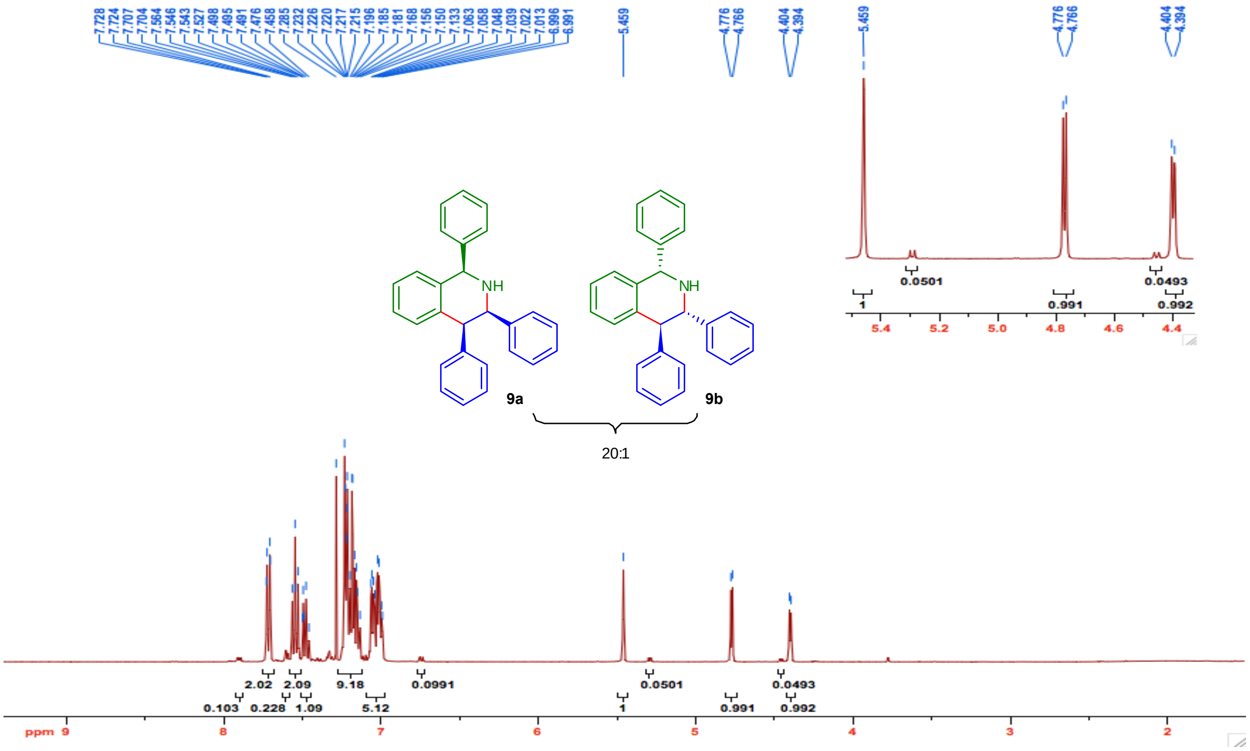

Supplementary Figure 79: ^{13}C NMR Spectrum of **9a** (100 MHz, $CDCl_3$)

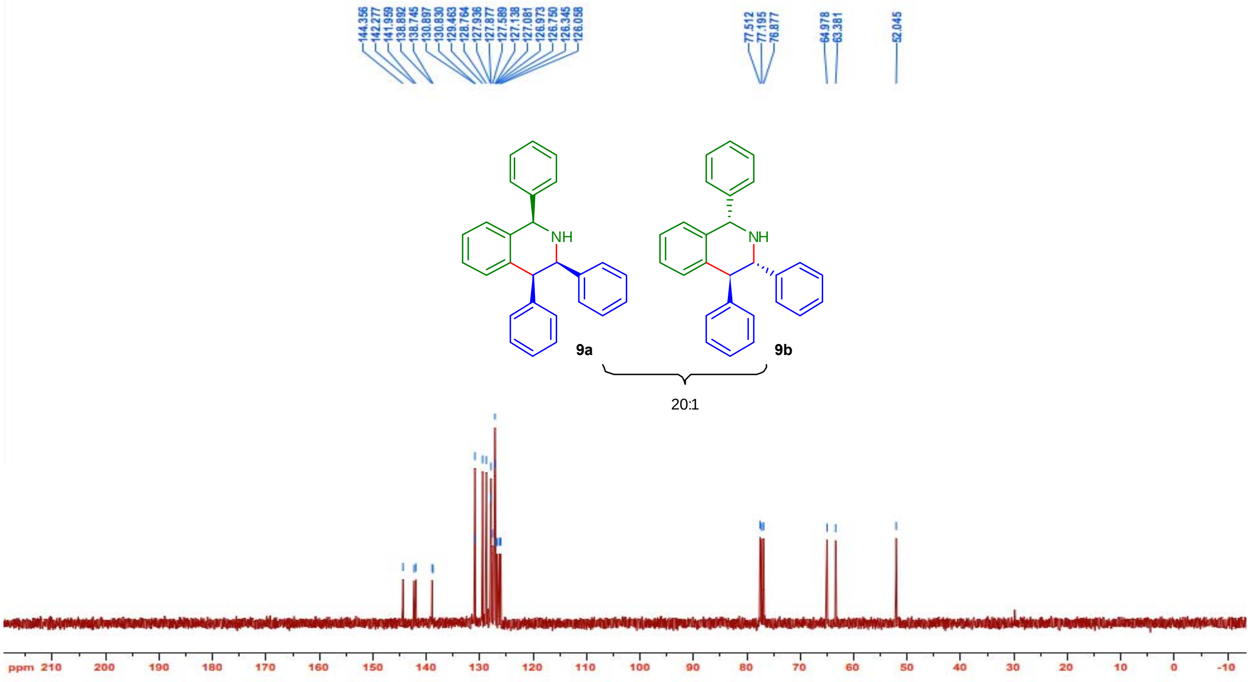

Supplementary Figure 80: ^{1}H NMR Spectrum of **5aac** (400 MHz, $CDCl_3$)

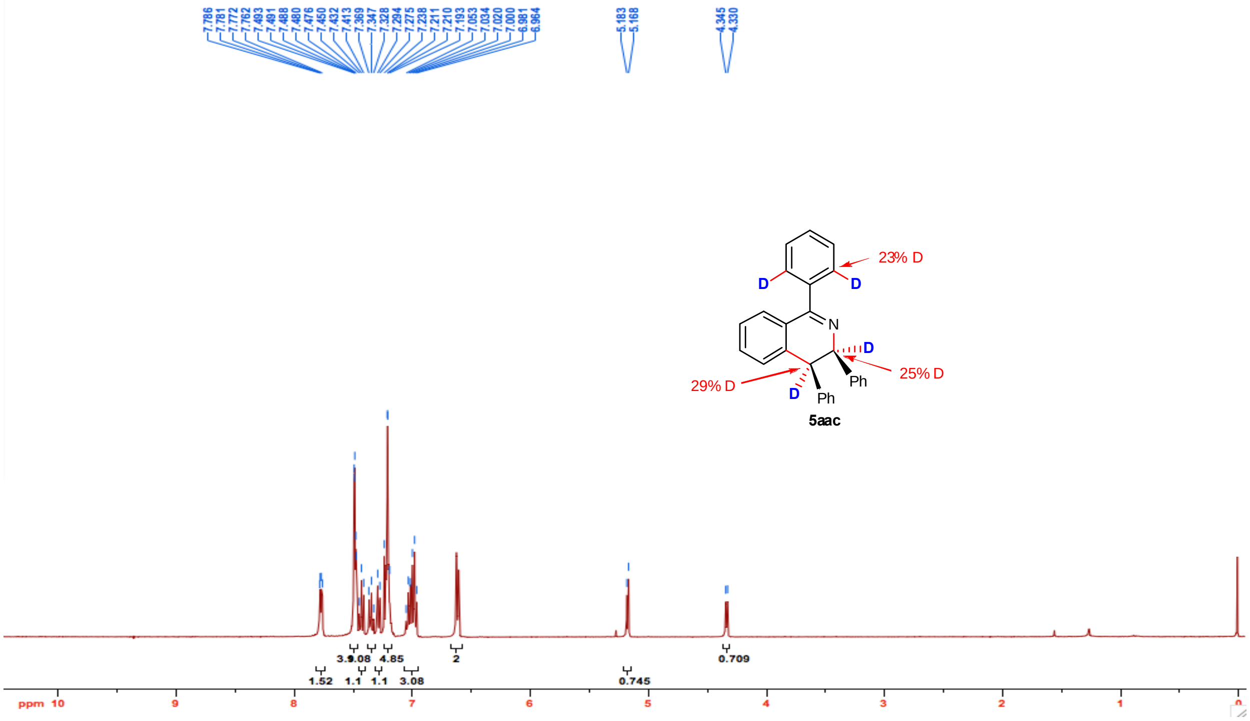

Supplementary Figure 81: ^{2}H NMR Spectrum of **5aac** (76.7 MHz, $CDCl_3$)

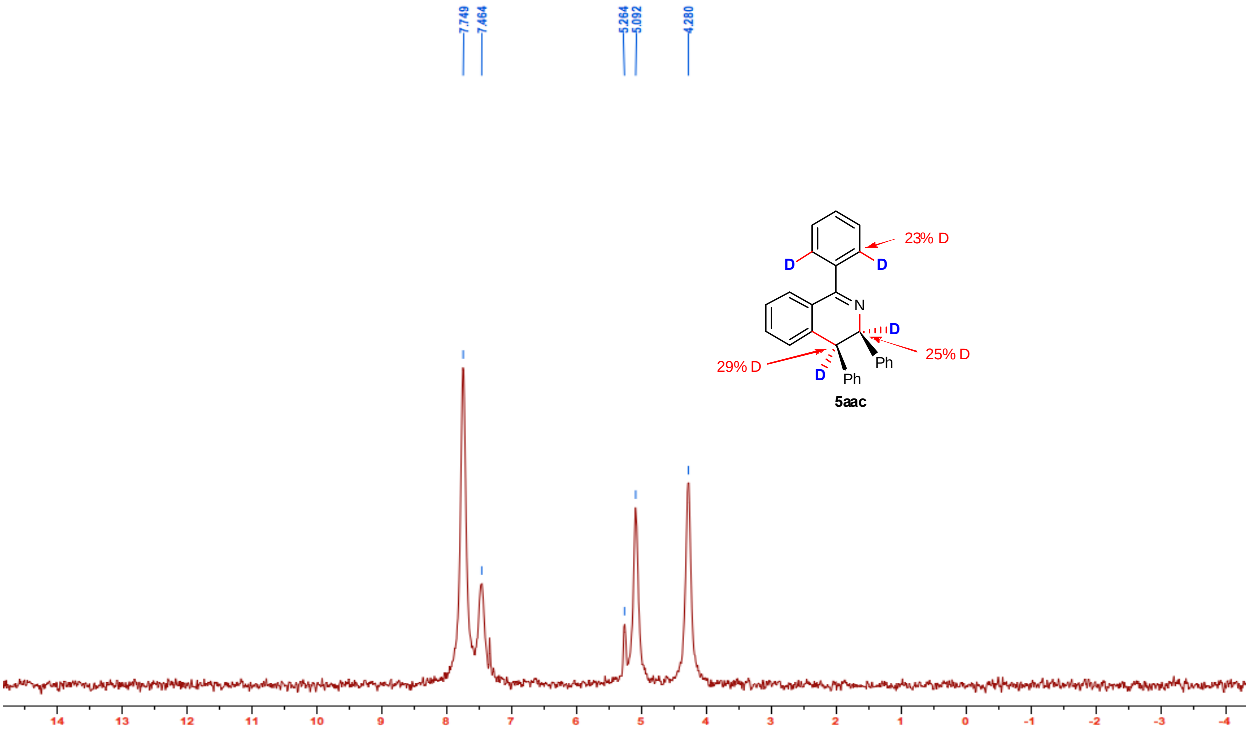

Supplementary Figure 82: ^{1}H NMR Spectrum of the mixture of **5aab/5aab’** (400 MHz, $CDCl_3$)

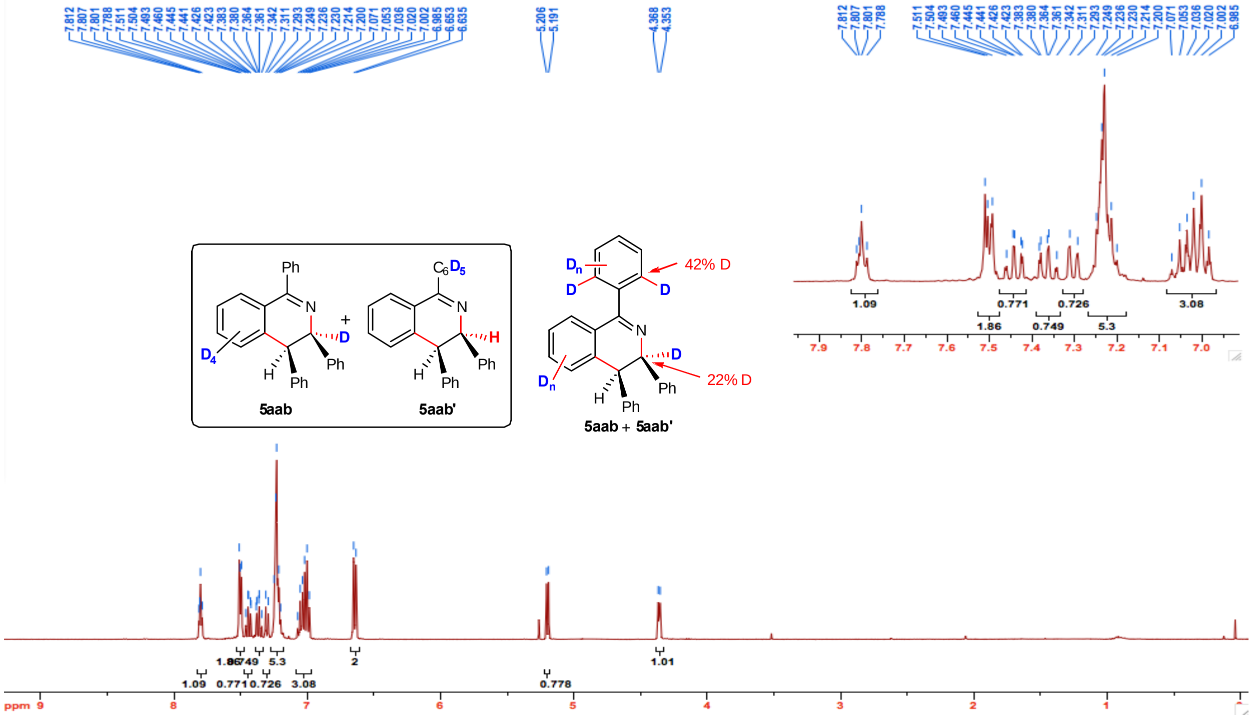

Supplementary Figure 83: ^{2}H NMR Spectrum of the mixture of **5aab/5aab’** (76.7 MHz, $CDCl_3$)

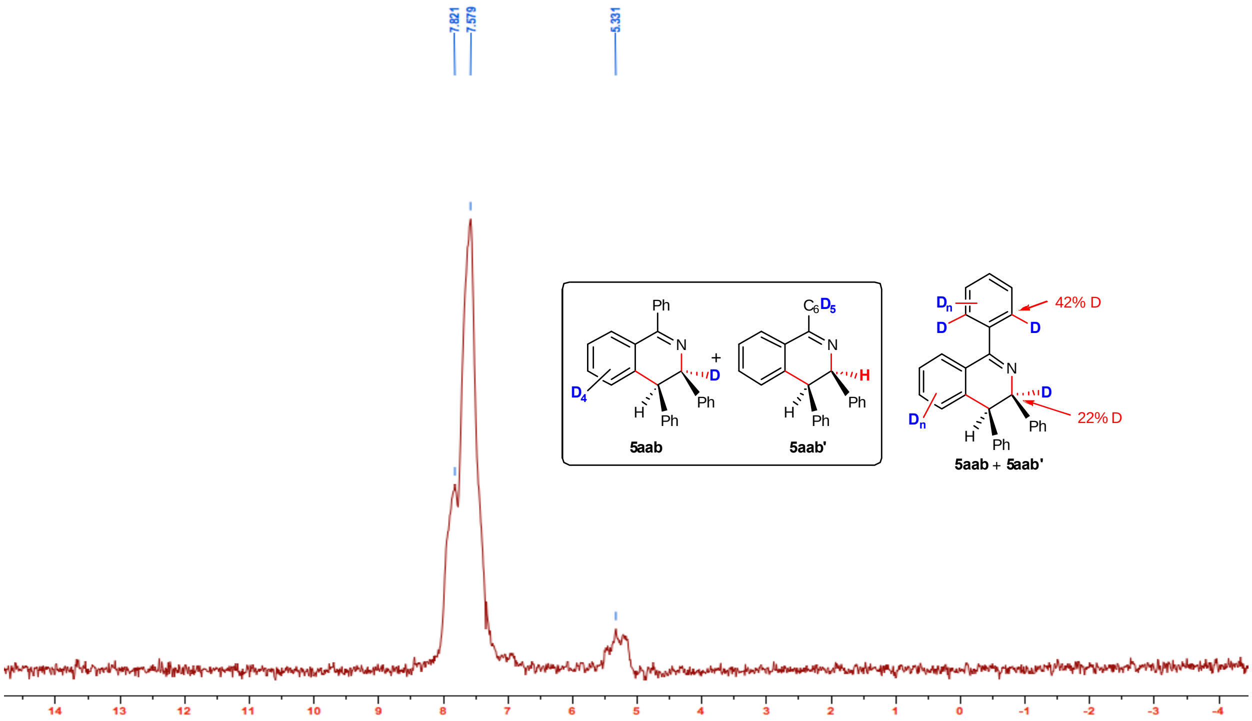

Supplementary Figure 84: ^{1}H NMR Spectrum of **12** (400 MHz, $CDCl_3$)

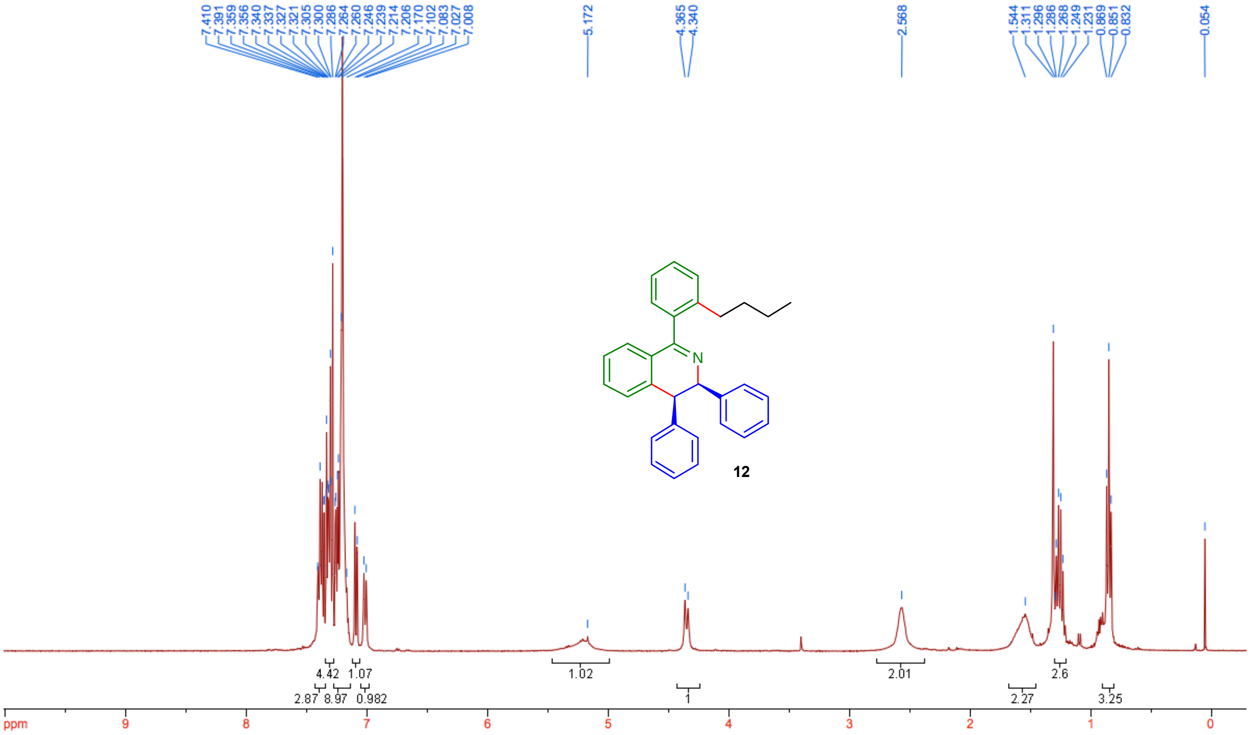

Supplementary Figure 85: ^{13}C NMR Spectrum of **12** (100 MHz, $CDCl_3$)

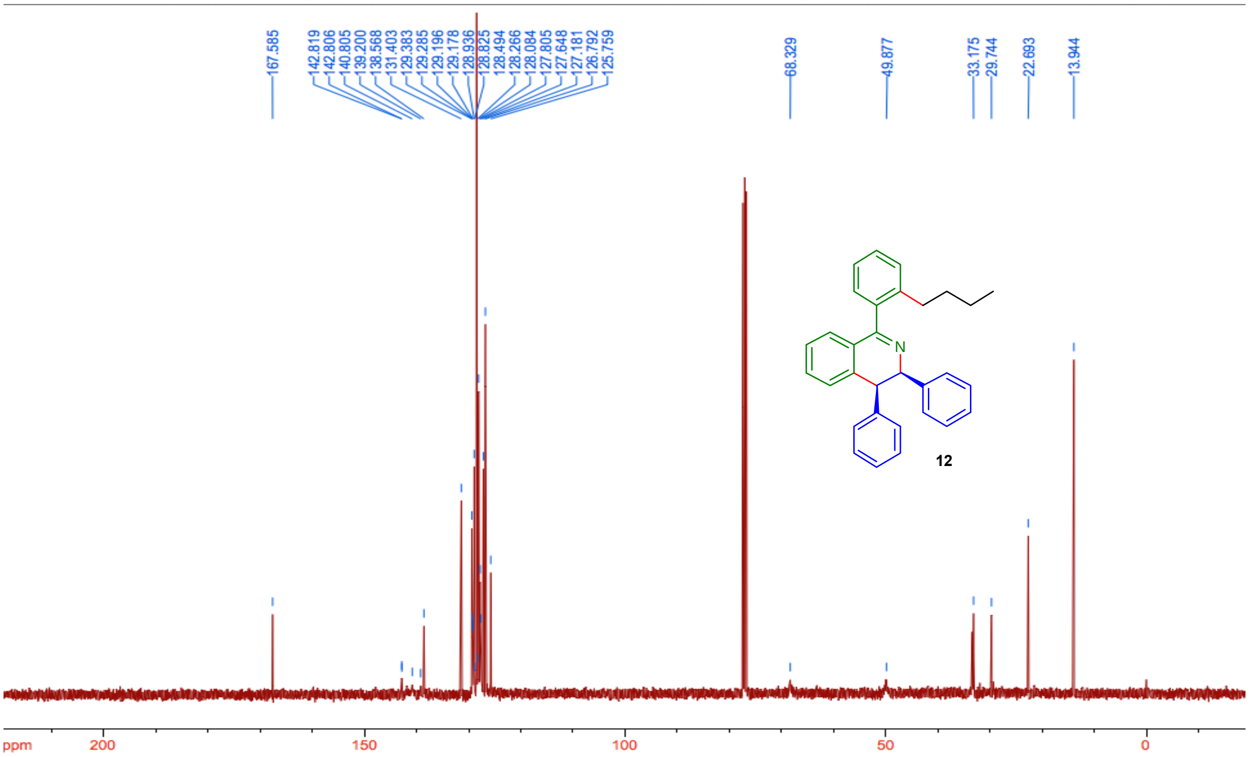

Supplementary Figure 86: ^{1}H NMR Spectrum of **13** (400 MHz, $CDCl_3$)

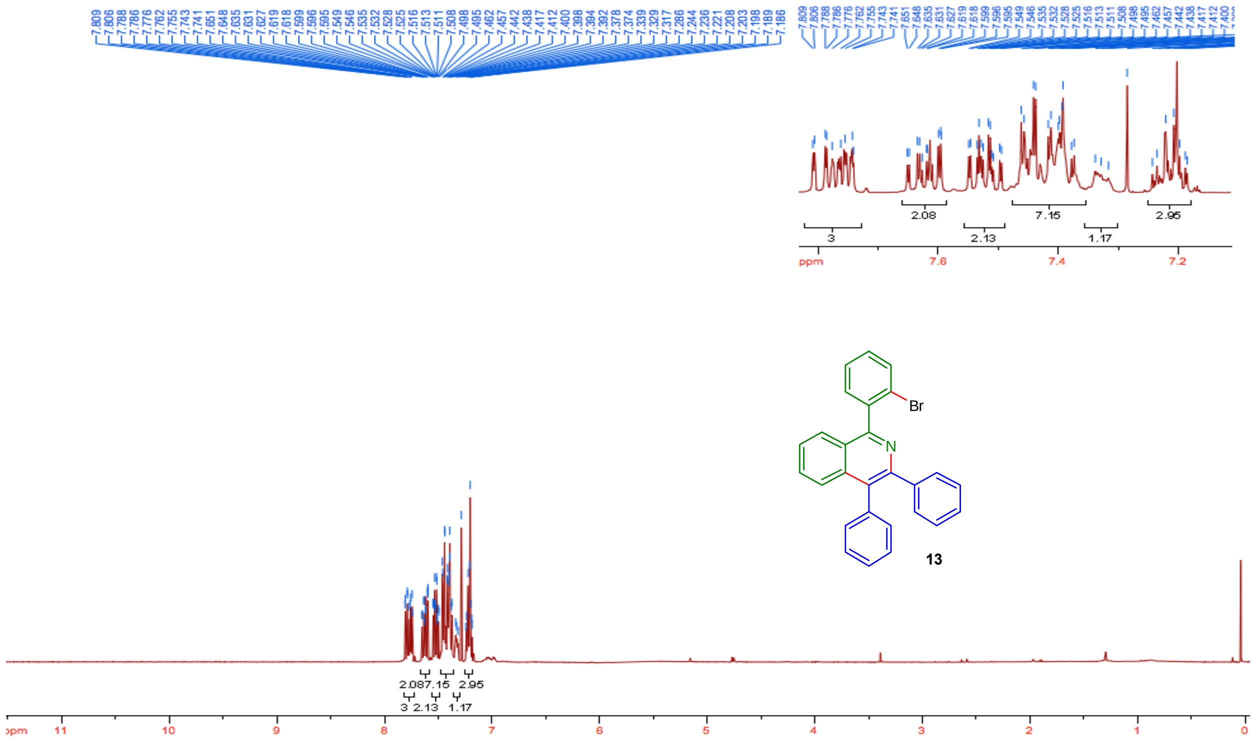

Supplementary Figure 87: ^{13}C NMR Spectrum of **13** (100 MHz, $CDCl_3$)

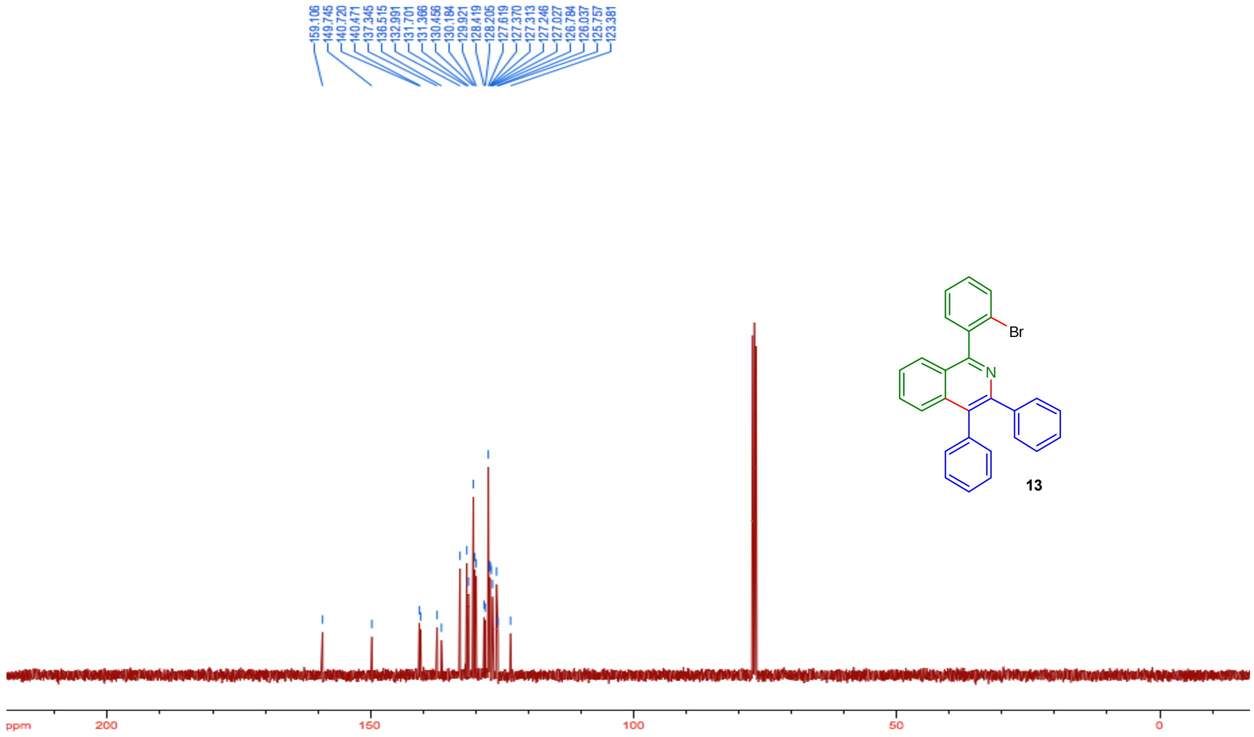

Supplementary Figure 88: Proposed pathways for Rh(I)-catalyzed N-H imine/alkyne annulations.

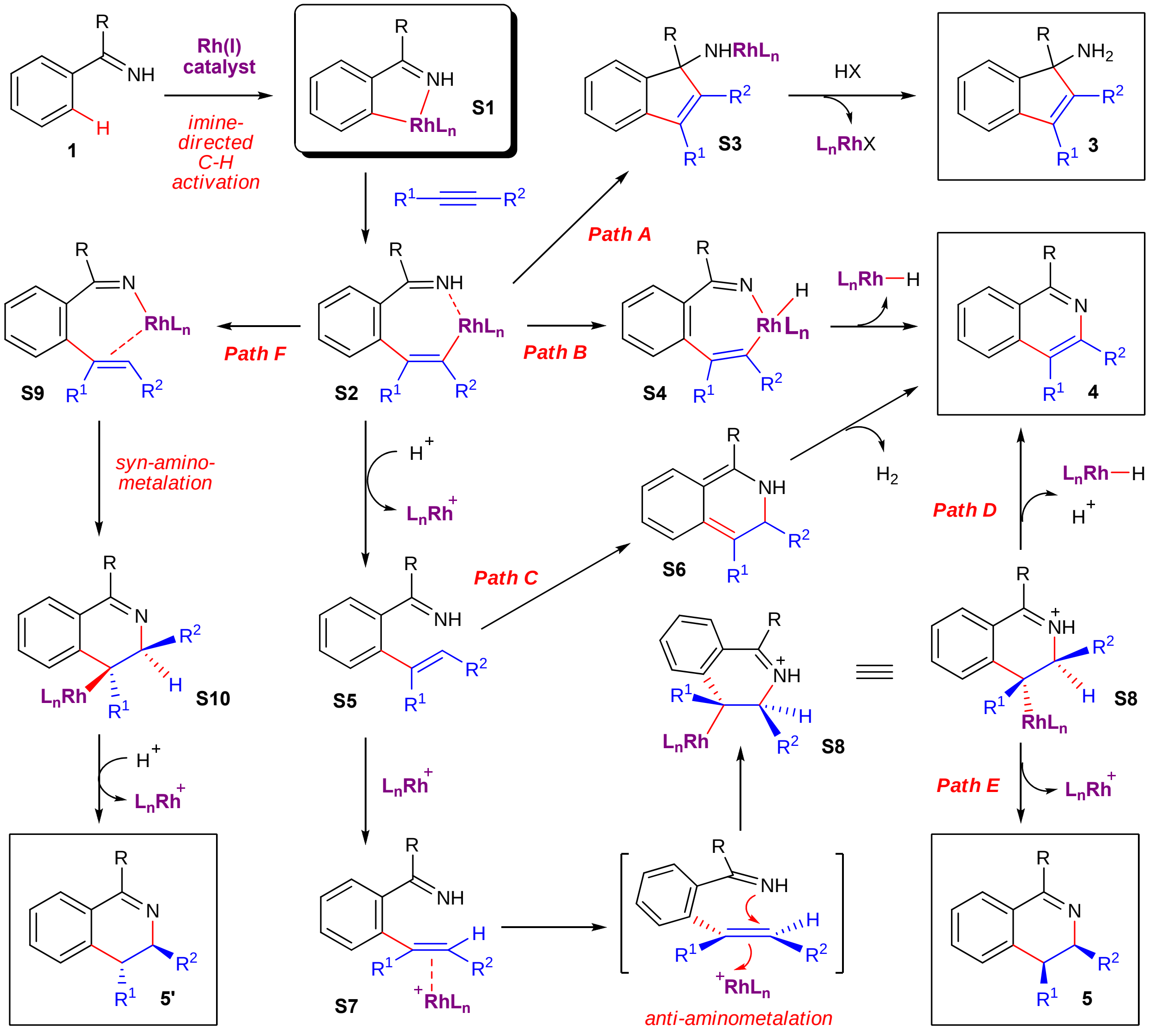

Path A: formation of [3+2] annulation product (**3**) via intramolecular imine insertion into the Rh-alkenyl linkage in alkyne coupling intermediate **S2**, followed by protonation of Rh amide **S3**.

Path B: formation of oxidative [4+2] annulation product (**4**) via N-H oxidative addition on Rh(I) center in intermediate **S2**, followed by C-N reductive elimination with the Rh(III) hydride intermediate **S4**.

Path C: protonation of **S2** to give *ortho*-alkenylation intermediate **S5**, followed by non-catalytic ring closure via 6π-electrocyclization, and aromatization-driven loss of H_2 to form product **4**.

Path D: alkene activation by π-complexation between **S5** and cationic Rh(I) center, followed by intramolecular alkene attack by the N-H imine nucleophile to give *anti*-aminometalation intermediate **S8**, and subsequent β-H elimination to form product **4**.

Path E: formation of desired redox-neutral [4+2] annulation product **5** via protonation of Rh-alkyl bond in intermediate **S8** that may involve intra- or intermolecular proton transfer with the iminium cation. The protonation process is assumed to occur in stereospecific retention to give the *cis*-4,5-disubstituted diastereomer.

Path F: formation of alkene-chelated Rh(I) iminyl intermediate **S9** via proton exchange with **S2**, followed by *syn*-aminometalation by intramolecular alkene insertion into the Rh-iminyl linkage to form intermediate **S10** (a stereoisomer of **S8**). Subsequent protonation of the Rh-alkyl bond occurred with stereospecific retention to afford the *trans*-isomer of 3,4-dihydroisoquinoline **5'** (not detected during our catalysis development).

Additional Note: In principle, reactive intermediate **S6** may isomerize to give redox-neutral [4+2] annulation product **5**. However, the exclusive formation of the *cis*-isomers of **5**, which should be less stable than the corresponding *trans*-isomers, supports a catalytic pathway for C-N bond formation/ring-closure rather than electrocyclization.

Supplementary Figure 89. ORTEP diagram (30% probability) of *cis*-1,3,4-triphenyl-3,4-dihydroisoquinoline (**5aa**), *cis*-3-ethyl-1,4-diphenyl-3,4-dihydroisoquinoline (**5an**) and *cis*-1-methyl-3,4-diphenyl-5-trifluoromethyl-3,4-dihydroisoquinoline (**5na**). All aromatic hydrogen atoms and the ethyl hydrogen atoms in **5an** are omitted for clarity.

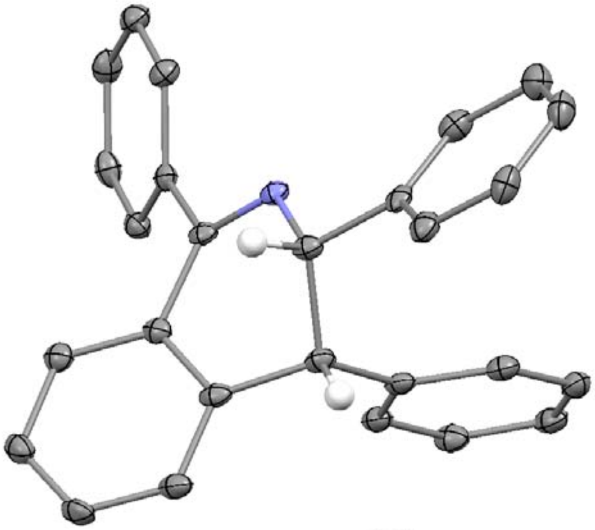

5aa

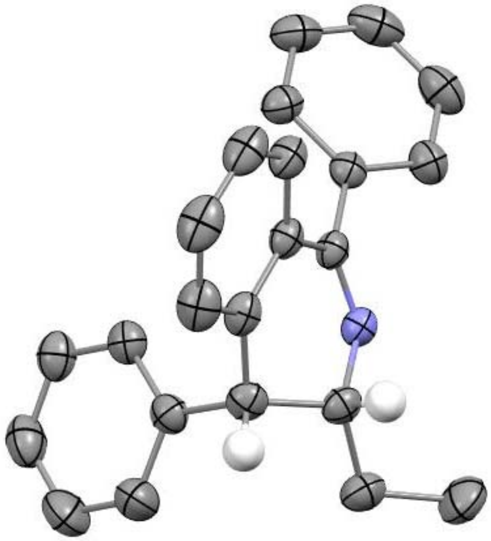

5an

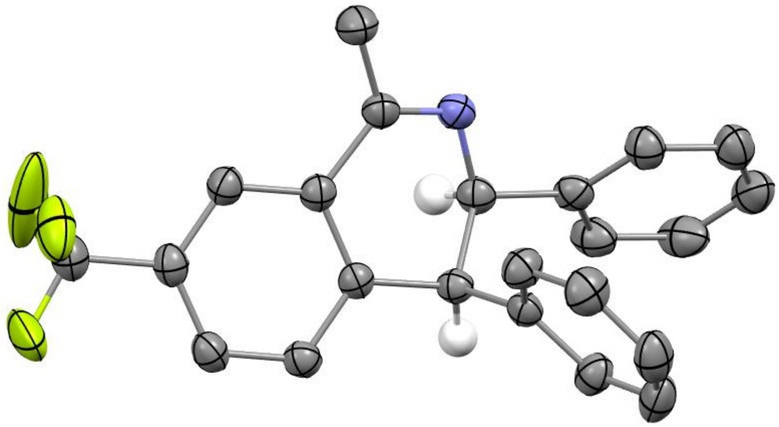

5na

Supplementary Figure 90. ORTEP diagram (30% probability) of the all-*cis* diastereomer of 1,3,4-triphenyl-1,2,3,4-tetrahydroisoquinoline (**9a**) and the cyclometalated Cp*Rh(III) complex **11**. All aromatic hydrogen atoms and hydrogen atoms from the Cp* moiety in **11** are omitted for clarity.

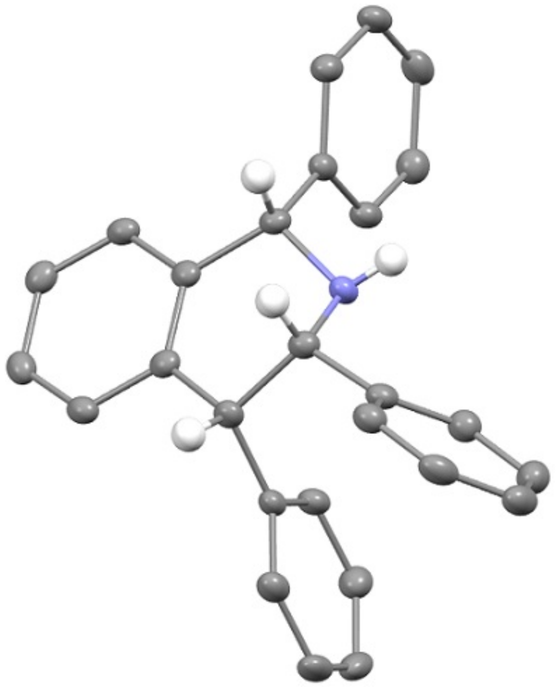

9a

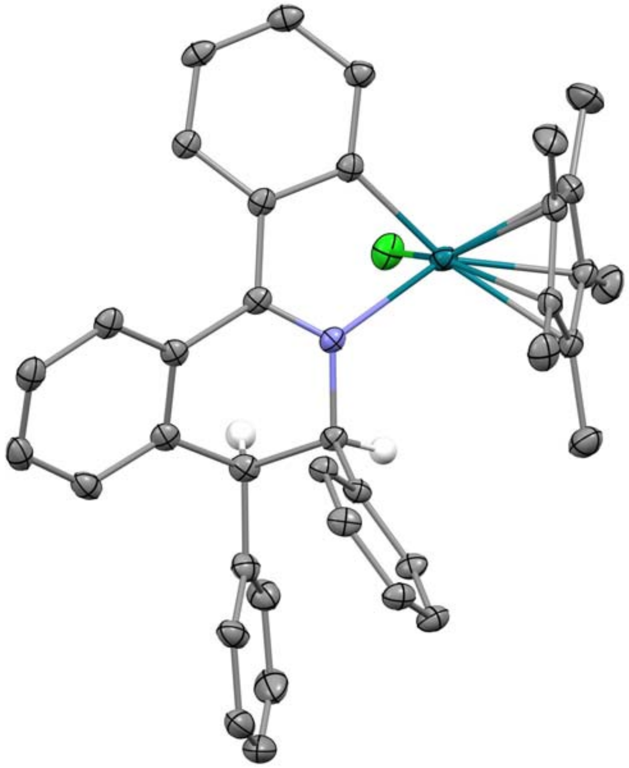

11

Supplementary Table 1. Summary of cell parameters, data collection and structural refinements for **5aa**, **5an**, **5na**, **9a** and **11**.

	**5aa (rxm 13)** CCDC 1424375	**9a (rxm 39)** CCDC 1424376	**5na (rxm 46)** CCDC 1424377	**11 (rxm 50)** CCDC 1424378	**5an (rxm 52)** CCDC 1442483
**Formula**	$C_{27}H_{21}N$	$C_{27}H_{23}N.1/2\ CHCl_3$	$C_{23}H_{18}F_3N$	$C_{38}H_{35}D_2Cl_3NRh$	$C_{23}H_{21}N$
**FW**	359.45	421.14	365.38	718.95	311.41
**cryst. size_max [mm]**	0.162	0.208	0.27	0.12	0.238
**cryst. size_mid [mm]**	0.095	0.2	0.2	0.09	0.191
**cryst. size_min [mm]**	0.045	0.075	0.12	0.02	0.161
**cryst. system**	monoclinic	triclinic	triclinic	triclinic	orthorhombic
**Space Group, Z**	$P2_1/n$, 4	P-1, 4	P-1, 2	P-1, 4	P 2ac 2ab
**a [Å]**	9.91151(4)	9.5479(4)	8.0095(2)	12.0885(6)	9.4007(3)
**b [Å]**	7.5788(3)	11.8989(5)	10.4347(2)	13.8960(6)	12.4572(4)
**c [Å]**	25.8148(13)	20.1528(9)	11.6372(3)	21.7793(11)	15.2327(6)
**α [Å]**	90.0	86.386(2)	76.4770(10)	72.428(3)	90.0
**ß [Å]**	95.699(4)	80.950(2)	86.8240	84.272(4)	90.0
**γ [Å]**	90.0	71.372(2)	76.469(2)	73.638(3)	90.0
**V [$Å^3$]**	1929.6(2)	2142.40(16)	919.39(4)	3346.1(3)	1783.85(11)
**ρ_{calc} [g/cm^3]**	1.237	1.306	1.320	1.427	1.160
**μ [mm^{-1}]**	0.542	2.244	0.815	6.531	0.506
**Radiation Type**	Cu	Cu	Cu	Cu	Cu
**F(000)**	760	884	380	1472	664
**no of measured refl.**	10642	19642	11205	26949	6615
**no of indep. refl.**	3277	7379	3149	11084	2761
**no of refl. (I ≥ 2σ)**	2729	6556	2499	9133	2502

**Resolution [Å]**	0.84	0.84	0.84	0.84	0.84
**R1/wR2 (I ≥ 2σ)[a] [%]**	8.89/21.5	3.42/8.70	3.88/10.1	6.65/17.5	3.30/8.18
**R1/wR2 (all data) [%]**	10.2/22.1	3.95/9.04	4.99/10.9	8.12/18.5	3.78/8.5

[a] $R1 = \Sigma||F_O| - |F_C|| / \Sigma|F_O|$, $wR2 = [\Sigma w[(F_O)^2 - (F_C)^2]^2 / \Sigma w(F_O{}^2)^2]^{1/2}$ for $F_O{}^2 > 2\sigma(F_O{}^2)$, $w = [\sigma^2(F_O)^2 + (AP)^2 + BP]^{-1}$ where $P = [(F_O)^2 + 2(F_C)^2] / 3$.

Supplementary Discussion: Result Analysis of the Deuterium Labeling Experiments

The experiments followed the general procedure for Rh(I)-catalyzed redox-neutral [4+2] annulation as described above. All reactions were carried out using diphenylacetylene (**2a**) as the alkyne substrate. No additional deuterium or proton sources were added when using partially deuterated benzophenone imine (***d*₁-1a** or ***d*₅-1a**)[1] as the N-H imine substrate. When using non-deuterated benzophenone imine (**1a**) as the N-H imine substrate, 0.1 mL MeOD (99.8% deuterium-enriched) was added as the deuterium source. The products were further purified by flash-column chromatography (1% ethyl acetate in hexane) to give partially deuterated **5aa** (compounds **5aaa-5aac** in the manuscript) as a white solid and in 85-87% isolated yields.

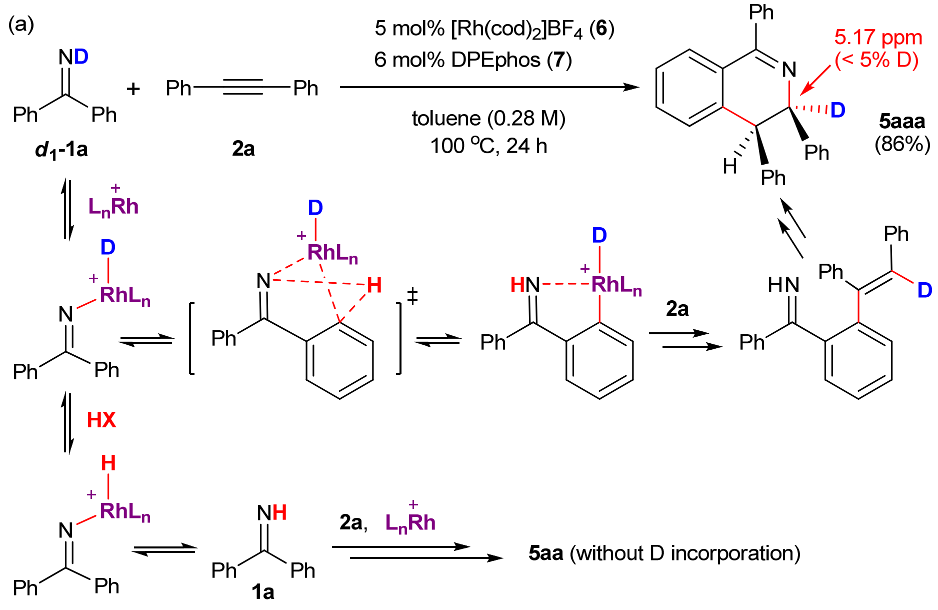

N-deuterated benzophenone imine (***d*$_1$-1a**, >95% D) reacted with **2a** to give product **5aaa** in 86% isolated yield. ^{1}H and ^{2}H NMR spectroscopy indicated that **5aaa** only contained trace amount of deuterium (< 5% D incorporation) at C3 position (δ = 5.17 ppm). The significant D loss was proposed to occur by N-D oxidative addition with Rh(I) center and a rapid H/D exchange between the resulting Rh(III) complex and trace moisture or acid in the reaction media. The regioselective intramolecular D transfer to C3 was proposed to occur by a less competitive pathway of 1,4-Rh migration with the Rh(III) intermediate (likely via a concerted transition state structure) that retained the Rh-D linkage, followed by alkyne insertion and N-heterocyclization steps (see Path E in Supplementary Figure 88 for details).

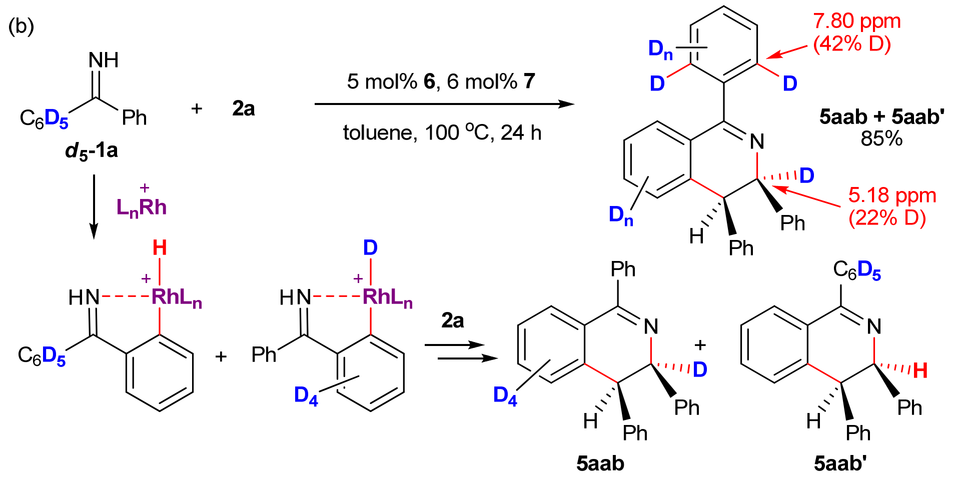

C_6D_5(Ph)C=NH (***d*$_5$-1a**, >95% D) reacted with **2a** to give an inseparable mixture of products **5aab** and **5aab'** in 85% isolated yield. ^{1}H and ^{2}H NMR spectroscopy indicated that **5aab/5aab'** contained a combined 22% D at C3 position (δ = 5.18 ppm). In addition, **5aab/5aab'** contained a number of aromatic deuteriums, among which 42% D could be determined at each *ortho* position of the 1-phenyl group (δ = 7.80 ppm, 84% in total). NMR signals for other aromatic protons or deuteriums were not resolved well enough for integration. The regioselective intramolecular D transfer to C3 was consistent with the proposed C-H alkenylation pathway as summarized above in Part (a) and described in more details in Supplementary Figure 88(Path E). The low D% at C3 and partial D retention at *ortho* positions of the 1-phenyl group suggested a combined effect of primary KIE for rate-determining C-H activation (C_6H_5 vs. C_6D_5) and H/D exchanges processes that were described above in Part (a). Assuming that inter- and intramolecular H/D exchange

processes were slow (other than those involving the imine N-H moiety), the numbers of D% in **5aab/5aab’** could be used to evaluate the KIE value for the cyclometalation step by C-H oxidative addition as follows: maximum value for KIE = k_H/k_D = (1 – 0.22)/0.22 = 3.5. However, an accurate determination of KIE was made difficult due to competitive H/D exchange processes.

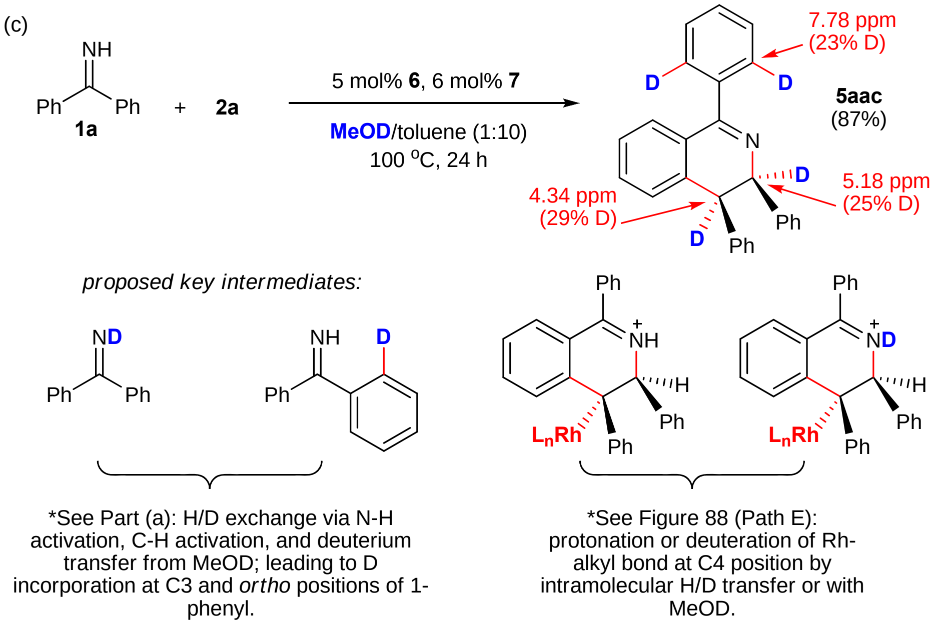

Non-deuterated benzophenone imine (**1a**) reacted with **2a** in the presence of added MeOD (99.8% D, 0.1 mL) to give product **5aac** in 87% isolated yield. ^{1}H and ^{2}H NMR spectroscopy indicated that **5aac** contained 25% D at C3 position (δ = 5.18 ppm), 29% D at C4 position (δ = 4.34 ppm), and 23% D at each *ortho* position of the 1-phenyl group (δ = 7.78 ppm, 46% in total). NMR signals for other aromatic protons or traces of deuteriums were not resolved well enough for integration. The partial D incorporation at C3 and *ortho* positions of 1-phenyl group could be rationalized by inter- and intramolecular H/D exchange processes as described above in Part (a). The partial D incorporation at C4 suggested that the protonation of Rh-alkyl linkage following the intramolecular alkene hydroamination/N-heterocyclization process may involve intramolecular proton transfer from the iminium proton (as the major pathway) or iminium deuterium (minor pathway), or intermolecular deuterium transfer from MeOD as an external Brønsted acid (minor pathway).

Supplementary Methods

General Experimental Procedures and Reagent Availability

Unless otherwise noted, all manipulations were carried out under a N_2 atmosphere using standard Schlenk-line or glovebox techniques. All glassware was oven-dried for at least 1 h prior to use. THF, toluene, and hexane were degassed by purging with N_2 for 45 min and dried with a solvent purification system (MBraun MB-SPS). Dioxane was distilled over sodium metal and freshly used. Except for commercially available benzophenone imine (**1a**), aromatic N-H ketimines were prepared by a reported general procedure of reactions between benzonitrile derivatives and organolithium reagents.[2,3] Other reagents and substrates were purchased from commercial vendors and were used as received. Organic solutions were concentrated by rotary evaporation at ~10 torr. Flash column chromatography was performed with 58 Å pore size neutral alumina purchased from Sigma Aldrich. GC analyses were performed on a Shimadzu GC-2010 with *n*-dodecane as the internal standard. ^{1}H NMR spectra were obtained on a 400 MHz spectrometer, and chemical shifts were recorded relative to residual protiated solvent. ^{13}C NMR spectra were obtained at 100.6 MHz, and chemical shifts were recorded to the solvent resonance. ^{2}H NMR spectra were obtained on a 76.7 MHz spectrometer. ^{1}H, ^{2}H and ^{13}C NMR chemical shifts were reported in parts per million downfield from tetramethylsilane ($\delta = 0$). ^{19}F NMR spectra were obtained at 376.3 MHz, and all chemical shifts were reported in parts per million upfield of CF_3COOH (δ = -78.5). High-resolution mass spectra (HRMS) were obtained at a Bruker Daltronics BioTOF HRMS spectrometer.

The diastereoselective hydride reduction of *cis*-1,3,4-triphenyl-3,4-dihydroisoquinoline (**5aa**) to form the all-*cis* diastereomer of 1,3,4-triphenyl-1,2,3,4-tetrahydroisoquinoline (**9a**) followed an analogous procedure reported by Bergman and Ellman.[4] Rh(III)-catalyzed C-H functionalization of **9a** to form products **12** and **13** followed an analogous procedure reported by Li[5] and Glorius[6] respectively.

Single crystal X-ray diffraction data of **5aa**, **5an**, **5na**, **9a** and **11** were collected on a Bruker Apex Duo diffractometer with a Apex 2 CCD area detector at T = 100K. Cu radiation was used. All structures were processed with Apex 2 v2010.9-1 software package (SAINT v.

7.68A, XSHELL v. 6.3.1). Direct method was used to solve the structures after multi-scan absorption corrections. Details of data collection and refinement are given in Table S2.

General Procedure for Rh(I)-Catalyzed Redox-Neutral [4+2] Annulation

Method A: For internal alkyne substrate scope. Into a 4 mL scintillation vial equipped with a magnetic stir bar was placed $[Rh(cod)_2]BF_4$ (**6**, 5.6 mg, 0.014 mmol, 0.050 equiv), DPEphos (**7**,8.9 mg, 0.017 mmol, 0.060 equiv), and 1.0 mL of Toluene. Next, N-H ketimine **1** (0.28 mmol, 1.0 equiv) and internal alkyne **2** (0.31 mmol, 1.1 equiv) were transferred into the vial. The vial was sealed with a silicone-lined screw-cap, transferred out of the glovebox, and stirred at 100 °C for 24 hours. After the reaction mixture was cooled to room temperature, all volatile materials were removed under reduced pressure. Further purification was achieved by flash-column chromatography using neutral alumina. Isolated yields are based on the average of two runs under identical conditions.

Method B: For N-H ketimine substrate scope. Into a 4 mL scintillation vial equipped with a magnetic stir bar was placed $[Rh(cod)_2]BF_4$ (**6**, 5.7 mg, 0.014 mmol, 0.050 equiv), DPEphos (**7**,9.1 mg, 0.017 mmol, 0.060 equiv), and 1.0 mL of Toluene. Next, N-H ketimine **1** (0.31 mmol, 1.1 equiv) and internal alkyne **2** (0.28 mmol, 1.0 equiv) were transferred into the vial. The vial was sealed with a silicone-lined screw-cap, transferred out of the glovebox, and stirred at 100 °C for 24 hours. After the reaction mixture was cooled to room temperature, all volatile materials were removed under reduced pressure. Further purification was achieved by flash-column chromatography using neutral alumina. Isolated yields are based on the average of two runs under identical conditions.

Synthesis of Cyclometalated Rh(III) Complex 11. Into a 1 dram vial charged with a stir-bar was added $[Cp^*RhCl_2]_2$ (50 mg, 0.008 mmol), *cis*-1,3,4-triphenyl-3,4-dihydroisoquinoline (**5aa**, 0.18 mmol), and sodium acetate (0.20 mmol). Dichloromethane (2.0 mL) was added and the resulting solution was stirred at room temperature for 60 hrs. After Celite filtration,all volatiles were removed under reduced pressure to afford a darkred-colored powder that contains the mixture of complex **11** and remaining reactants. Further crystallization was carried out in a mixed solvent system of chloroform and layered with pentane to produce a small amount of single crystals suitable for X-ray diffraction analysis.

Analytical Data of Reported Products

***cis*-1,3,4-Triphenyl-3,4-dihydroisoquinoline (5aa):** Prepared from benzophenone imine (**1a**) and 1,2-diphenylethyne by the general procedure A. Chromatography (1% ethyl acetate in hexane) gave **5aa** as a white solid (87 mg, 88%). ^{1}H-NMR (400 MHz, $CDCl_3$): δ 4.38 (d, 1H, *J* = 6.0 Hz), 5.21 (d, 1H, *J* = 6.0 Hz), 6.66 (d, 2H, *J* = 6.4 Hz), 7.02–7.09 (m, 3H),7.24-7.27 (m, 5H), 7.31 (d, 1H, J = 7.6 Hz), 7.38 (d, 1H, *J* = 7.6 Hz), 7.44 (d, 1H, *J* = 8.0 Hz), 7.52-7.53 (m, 4H), 7.81-7.84 (m, 2H). ^{13}C-NMR (100 MHz, $CDCl_3$): δ 167.9, 141.63, 141.60, 139.3, 137.8, 131.6, 129.8, 129.7, 129.34, 129.32, 128.6, 128.53, 128.50, 128.4, 128.1, 127.8, 127.5, 126.9, 126.7, 66.5, 49.2. HRMS: calcd for $C_{27}H_{22}N^{+}$ 360.1747, found 360.1758.

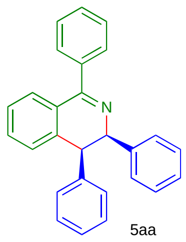

5aa

***cis*-1-Phenyl-3,4-di-*p*-tolyl-3,4-dihydroisoquinoline (5ab):** Prepared from benzophenone imine(**1a**) and 1,2-di-*p*-tolylethyne by the general procedure A. Chromatography (1% ethyl acetate in hexane) gave **5ab** as a white solid (95 mg, 89%). ^{1}H-NMR (400 MHz, $CDCl_3$): δ 2.17, (s, 3H), 2.30 (s, 3H), 4.29 (d, 1H, *J* = 6.0 Hz), 5.09 (d, 1H, *J* = 6.0 Hz), 6.51 (d, 2H, *J* = 8.0 Hz), 6.79 (d, 2H, *J* = 8.0Hz), 7.00 (d, 2H, *J* = 8.0 Hz), 7.09 (d, 2H, *J* = 8.4 Hz), 7.24 (d, 1H, *J* = 10.0 Hz), 7.32 (d, 1H, *J* = 6.4 Hz) 7.38 (d, 1H, *J* = 6.0 Hz), 7.43-7.47 (m, 4H), 7.73 (d, 2H, *J* = 8.0 Hz). ^{13}C-NMR (100 MHz, $CDCl_3$): δ 167.6, 141.9, 139.3, 138.4, 138.2, 136.2, 136.1, 134.7, 131.4, 129.7, 129.6, 129.3, 128.7, 128.5, 128.4, 128.3, 128.29, 128.28, 127.2, 66.2, 48.7, 21.4, 21.1. HRMS: calcd for $C_{29}H_{26}N^{+}$ 388.2060, found 388.2045.

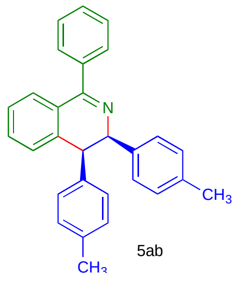

5ab

***cis*-3,4-bis(4-(*tert*-butyl)phenyl)-1-phenyl-3,4-dihydroisoquinoline (5ac):** Prepared from benzophenone imine(**1a**) and 1,2-bis(4-(*tert*-butyl)phenyl)ethyne by the general procedure A. Chromatography (1% ethyl acetate in hexane) gave **5ac** as a yellow solid (109 mg, 84%). ^{1}H-NMR (400 MHz, $CDCl_3$): δ 1.23, (s, 9H), 1.33 (s, 9H), 4.33 (d, 1H, *J* = 5.6 Hz), 5.16 (d, 1H, *J* = 6.0 Hz), 6.55 (d, 2H, *J* = 8.4 Hz), 7.01 (d, 2H, *J* = 8.4Hz), 7.10 (d, 2H, *J* = 8.4 Hz), 7.23-7.25 (m, 2H), 7.29-7.39 (m, 2H), 7.46-7.53 (m, 5H), 7.80

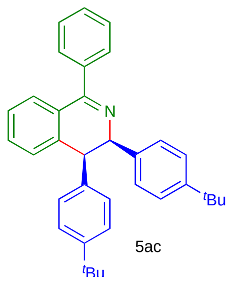

5ac

(d, 2H, J = 7.6 Hz). ^{13}C-NMR (100 MHz, $CDCl_3$): δ 167.4, 149.5, 149.1, 141.6, 139.2, 138.2, 134.4, 131.2, 129.4, 129.2, 129.1, 128.4, 128.3, 128.2, 128.1, 128.0, 127.0, 124.5, 124.3, 66.1, 48.5, 34.4, 34.2, 31.5, 31.3. HRMS: calcd for $C_{35}H_{37}N^+$ 472.3004, found 472.3017.

***cis*-3,4-Bis(4-methoxyphenyl)-1-phenyl-3,4-dihydro-isoquinoline (5ad):** Prepared from benzophenone imine (**1a**) and 1,2-bis(4-methoxyphenyl)ethyne by the general procedure A. Chromatography (3% ethyl acetate in hexane) gave **5ad** as a light-yellow oil (104mg, 90%). ^{1}H-NMR (400 MHz, $CDCl_3$): δ 3.67 (s, 3H), 3.78 (s, 3H), 4.28 (d, 1H, J = 6.0 Hz), 5.10 (d, 1H, J = 6.0 Hz), 6.58 (s, 4H), 6.78 (d, 2H, J = 8.4 Hz), 7.12 (d, 2H, J = 8.0 Hz), 7.26 (d, 1H, J = 7.2 Hz), 7.24 (d, 2H, J = 7.6 Hz), 7.41-7.43 (m, 2H), 7.47-7.76 (m, 5H), 7.77 (d, 2H, J = 9.2 Hz). ^{13}C-NMR (100 MHz, $CDCl_3$): δ 167.7, 158.6, 158.4, 141.9, 139.4, 133.8, 131.5, 130.8, 130.6, 129.9, 129.70, 129.65, 129.3, 129.2, 128.5, 128.4, 127.3, 113.5, 113.3, 65.9, 55.5, 55.3, 48.5. HRMS: calcd for $C_{29}H_{26}NO_2^+$ 420.1958, found 428.1970.

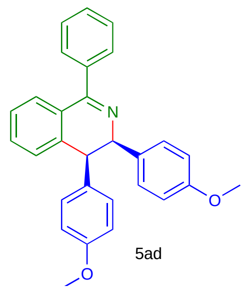

5ad

***cis*-3,4-Bis(4-fluorophenyl)-1-phenyl-3,4-dihydroisoquinoline (5ae):** Prepared from benzophenone imine (**1a**) and 1,2-bis(4-fluorophenyl)ethyne by the general procedure A. Chromatography (1% ethyl acetate in hexane) gave **5ae** as a white solid (104 mg, 95%). ^{1}H-NMR (400 MHz, $CDCl_3$): δ 4.31 (d, 1H, J = 5.6 Hz), 5.16 (d, 1H, J = 6.0 Hz), 6.61 (t, 2H, J = 8.4 Hz), 6.72 (t, 2H, J = 8.8Hz), 6.96 (t, 2H, J = 8.8 Hz), 7.22-7.30 (m, 3H), 7.32 (s, 1H), 7.40 - 7.54 (m, 5H), 7.80 (d. 2H, J = 4.0 Hz). ^{13}C-NMR (100 MHz, $CDCl_3$): δ 168.2, 163.3 (d, J(C,F) = 244.5 Hz), 163.1 (d, J(C,F) = 245.5 Hz), 141.3, 139.1, 137.5 (d, J = 3.0 Hz), 133.3 (d, J = 3.0 Hz), 131.9, 131.2 (d, J = 7.0 Hz), 130.1 (d, J = 8.0 Hz), 129.9, 129.3, 129.1, 128.7, 128.6, 128.5, 127.7, 115.1 (d, J = 21.1 Hz), 114.9 (d, J = 21.3 Hz), 65.6, 48.5. ^{19}F-NMR (376.3 MHz, $CDCl_3$): δ -116.08, -116.09. HRMS: calcd for $C_{27}H_{20}F_2N^+$ 369.1558, found 369.1539.

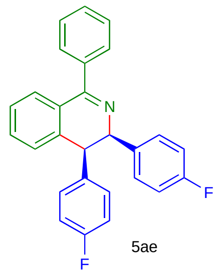

5ae

***cis*-3,4-Bis(4-chlorophenyl)-1-phenyl-3,4-dihydroisoquinoline (5af):** Prepared from

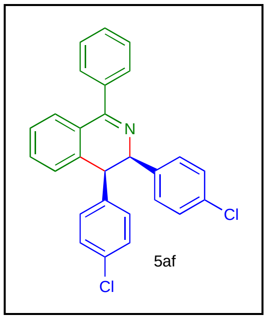

benzophenone imine (**1a**) and 1,2-bis(4-chlorophenyl)ethyne by the general procedure A. Chromatography (1% ethyl acetate in hexane) gave **5af** as a white solid (104 mg, 88%). ^{1}H-NMR (400 MHz, $CDCl_3$): δ 4.28 (d, 1H, *J* = 6.0 Hz), 5.13 (d, 1H, *J* = 6.0 Hz), 6.56 (d, 2H, *J* = 8.4 Hz), 6.99 (d, 2H, *J* = 8.4 Hz), 7.24-7.29 (m, 5H), 7.38–7.49 (m, 2H), 7.44 (d, 1H, *J* = 8.8 Hz), 7.50–7.52 (m, 4H), 7.76 (d, 2H, *J* = 7.6 Hz). ^{13}C-NMR (100.6 MHz, $CDCl_3$): δ 168.2, 140.9, 140.2, 138.9, 135.9, 132.8, 132.7, 131.9, 130.9, 129.99, 129.94, 129.3, 129.0, 128.7, 128.6, 128.44, 128.38, 128.2, 127.8, 65.5, 48.4. HRMS: calcd for $C_{27}H_{20}Cl_2N^+$ 428.0967, found 428.0952.

***cis*-1-Phenyl-3,4-bis(4-trifluoromethylphenyl)-3,4-dihydro-isoquinoline (5ag):** Prepared from

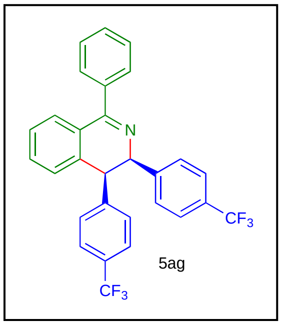

benzophenone imine (**1a**) and 1,2-bis(4-trifloromethylphenyl)ethyne by the general procedure A. Chromatography (1% ethyl acetate in hexane) gave **5ag** as a white solid (126 mg, 92%). ^{1}H-NMR (400 MHz, $CDCl_3$): δ 4.40 (d, 1H, *J* = 6.0 Hz), 5.25 (d, 1H, *J* = 6.0 Hz), 6.74 (d, 2H, *J* = 8.0 Hz), 7.26 – 7.28 (m, 3H), 7.42 – 7.48 (m, 3H), 7.49-7.56 (m, 7H), 7.78 (d, 2H, *J* = 8.0 Hz). ^{13}C-NMR (100 MHz, $CDCl_3$): δ 168.3, 145.4, 141.2, 140.3, 138.5, 132.4 (q, *J* = 69.4 Hz), 131.82, 131.75, 130.8 (q, *J* = 57.3 Hz), 129.9, 129.4 (q, *J* = 22.1 Hz), 128.8, 128.7, 128.5 (q, *J* = 18.1 Hz), 127.9, 125.7 (q, *J*(C,F) = 271.6 Hz), 125.4 (q, *J*(C,F) = 252.5 Hz), 125.1 (q, *J* = 6.0 Hz), 124.8 (q, *J* = 3.0 Hz), 122.9, 122.7, 65.5, 48.5. ^{19}F-NMR (376.3 MHz, $CDCl_3$): δ -15.3, -14.9. HRMS: $C_{29}H_{20}F_6N^+$ 496.1494, found 496.1474.

***cis*-3,4-Bis(3-methoxyphenyl)-1-phenyl-3,4-dihydro-isoquinoline (5ah):** Prepared from

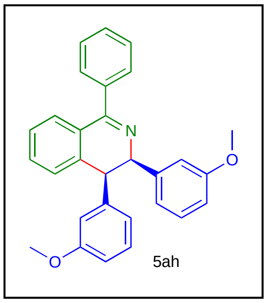

benzophenone imine (**1a**) and 1,2-bis(3-methoxyphenyl)ethyne by the general procedure A. Chromatography (3% ethyl acetate in hexane) gave **5ah** as a creamy white solid (90 mg, 78%). ^{1}H-NMR (400 MHz, $CDCl_3$): δ 3.50 (s, 3H), 3.61 (s, 3H), 4.31 (d, 1H, *J* = 6.0 Hz), 5.15 (d, 1H, *J* = 6.0 Hz), 6.18 (s, 1H), 6.25 (d, *J* = 7.6 Hz), 6.60 (d, 1H, *J* = 6.8 Hz), 6.77 (s, 2H), 6.89-6.95 (m, 2H), 7.16 -7.23 (m, 1H), 7.29-7.37 (m, 2H), 7.42- 7.50 (m, 5H), 7.78 (d, 2H, *J* = 7.6 Hz). ^{13}C-NMR (100 MHz, $CDCl_3$): δ 167.8, 159.7, 158.9, 143.4,

141.4, 139.3, 131.6, 129.7, 129.3, 129.23, 129.20, 128.9, 128.7, 128.5, 128.4, 127.4, 122.2, 120.9, 115.1, 114.0, 113.2, 112.8, 66.4, 55.4, 55.1, 49.1. HRMS: calcd for $C_{29}H_{26}NO_2^+$ 420.1958, found 420.1964.

***cis*-3,4-bis(3-fluorophenyl)-1-phenyl-3,4-dihydroisoquinoline (5ai):** Prepared from benzophenone imine (**1a**) and 1,2-bis(3-fluorophenyl)ethyne by the general procedure A. Chromatography (1% ethyl acetate in hexane) gave **5ai** as a white solid (96 mg, 88%). ^{1}H-NMR (400 MHz, $CDCl_3$): δ 4.35 (d, 1H, *J* = 6.0 Hz), 5.19 (d, 1H, *J* = 6.0 Hz), 6.61-6.47 (m, 2H), 6.76 (t, 1H, *J* = 8.4 Hz),6.93-7.01 (m, 2H), 7.08-7.16 (d, 2H), 7.24-7.29 (m, 1H), 7.32 (d, 1H, *J* = 7.6 Hz), 7.39 (t, 1H, *J* = 7.6 Hz), 7.47-7.55 (m, 5H), 7.81-7.84 (m, 2H). ^{13}C-NMR (100 MHz, $CDCl_3$): δ 168.2, 164.4 (d, *J*(C,F) = 243.7 Hz), 161.9 (d, *J*(C,F) = 244.1 Hz), 144.5 (d, *J* = 7.8 Hz), 140.8, 140.2 (d, *J* = 7.2 Hz), 131.9,130.0, 129.7, 129.6, 129.4, 129.3, 129.1, 114.0 (d, *J* = 10.9 Hz), 113.8 (d, *J* = 10.7 Hz), 128.8, 128.61, 128.55, 127.9, 125.3 (d, *J* = 2.5 Hz), 116.6 (d, *J* = 22.2 Hz), 115.7 (d, *J* = 22.3 Hz), 114.0 (d, 10.9 Hz), 113.8 (d, 10.7 Hz), 65.6, 48.8. ^{19}F-NMR (376.3 MHz, $CDCl_3$): δ -113.4, -113.6. HRMS: calcd for $C_{27}H_{22}N^+$ 396.1558, found 396.1565.

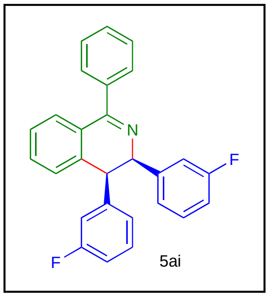

***cis*-3,4-bis(2-fluorophenyl)-1-phenyl-3,4-dihydroisoquinoline (5aj):** Prepared from benzophenone imine (**1a**) and 1,2-bis(2-fluorophenyl)ethyne by the general procedure A. Chromatography (1% ethyl acetate in hexane) gave **5aj** as a white solid (88 mg, 81%). ^{1}H-NMR (400 MHz, $CDCl_3$): δ 5.08 (d, 1H, *J* = 6.4 Hz), 5.65 (d, 1H, *J* = 6.4 Hz), 6.78 (t, 1H, *J* = 8.4 Hz), 6.99-7.01 (m, 3H),7.07-7.18 (m, 2H), 7.24-7.31 (m, 2H), 7.42-7.47 (m, 2H), 7.42-7.47 (m, 2H), 7.52-7.57 (m, 1H), 7.59-7.69 (m, 4H), 7.89-7.93 (m, 2H). ^{13}C-NMR (100 MHz, $CDCl_3$): δ 168.4, 162.1 (d, *J*(C,F) = 243.5 Hz), 161.6 (d, *J*(C,F) = 244.6 Hz), 140.3, 139.1, 138.3 (d, J = 3.1 Hz), 137.7, 132.5, 131.8, 130.8 (d, *J* = 4.8 Hz), 130.6 (d, *J* = 3.6 Hz), 130.2, 129.7, 129.2, 129.1, 128.9, 128.5, 128.4, 127.6, 125.4, 115.1 (d, *J* = 23.4 Hz), 114.5 (d, *J* = 21.7 Hz), 59.2, 38.2. ^{19}F-NMR (376.3 MHz, $CDCl_3$): δ -116.7, -120.1. HRMS: calcd for $C_{27}H_{22}N^+$ 396.1564, found 360.1577.

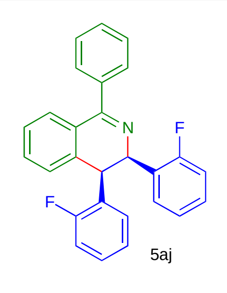

***cis*-1-Phenyl-3,4-di(thiophen-2-yl)-3,4-dihydroisoquinoline (5ak):** Prepared from benzophenone imine (**1a**) and 1,2-di(thiophen-2-yl)ethyne by the general procedure A. Chromatography (1% ethyl acetate in hexane) gave **5ak** as a white solid (92 mg, 89%). ^{1}H-NMR (400 MHz, $CDCl_3$): δ 4.75 (d, 1H, J = 5.2 Hz), 5.39 (d, 1H, J = 4.8 Hz), 6.52 (d, 1H, J = 3.2 Hz), 6.75 (t, 1H, J = 4.8 Hz), 7.01–7.06 (m, 2H), 7.14–7.22 (m, 2H), 7.40–7.42 (m, 2H), 7.46-7.55 (m, 5H), 7.77-7.80 (m, 2H). ^{13}C-NMR (100 MHz, $CDCl_3$): δ 167.7, 145.6, 141.5, 140.2, 138.4, 131.4, 129.6, 129.3, 128.5, 128.24, 128.21, 127.8, 127.6, 126.6, 126.4, 126.1, 124.7, 124.4, 62.1, 44.9. HRMS: calcd for $C_{23}H_{18}NS_2^+$ 372.0875, found 372.0859.

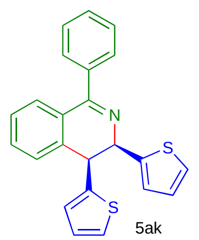

5ak

***cis*-3,4-Diethyl-1-phenyl-3,4-dihydroisoquinoline (5al):** Prepared from benzophenone imine (**1a**) and 3-hexyne by the general procedure A. Chromatography (1% ethyl acetate in hexane) gave **5al** as a light yellow liquid (59 mg, 81%). ^{1}H-NMR (400 MHz, $CDCl_3$): δ 0.83 (t, 3H, J = 7.2 Hz), 1.13 (t, 3H, J = 7.2 Hz), 1.35-1.43 (m, 1H), 1.64 (q, 1H, J = 7.2Hz), 1.83 (q, 1H, J = 6.8 Hz), 1.99-2.08 (m, 1H), 2.57 (sept, 1H, J = 10.0 Hz), 3.39 (d, 1H, J = 5.2 Hz), 7.20 (t, 3H, J = 7.2 Hz), 7.34 (d, 1H, J = 6.8 Hz), 7.39 (d, 3H, J = 4.8 Hz), 7.59 (d, 2H, J = 7.6 Hz). ^{13}C-NMR (100 MHz, $CDCl_3$): δ 166.7, 143.7, 139.4, 130.2, 129.4, 129.1, 128.7, 128.5, 128.4, 128.2, 126.7, 62.8, 41.7, 26.1, 19.5, 12.4, 11.7. HRMS: calcd for $C_{19}H_{22}N^+$ 264.1747, found 264.1757.

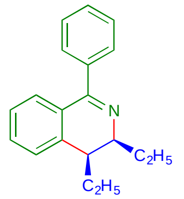

5al

***cis*-3,4-Dipropyl-1-phenyl-3,4-dihydroisoquinoline (5am):** Prepared from benzophenone imine (**1a**) and 4-octyne by the general procedure A. Chromatography (1% ethyl acetate in hexane) gave **5am** as a light yellow liquid (68 mg). This product was contaminated with ~10% of the corresponding isoquinoline byproduct (generated by product decomposition during separation and purification), and the yield for **5am** was estimated to be 76% by ^{1}H NMR analysis (see Figure 29). ^{1}H-NMR (400 MHz, $CDCl_3$): δ 0.86 (t, 3H, J = 7.2 Hz), 1.01 (t, 3H, J = 7.2 Hz), 1.14 (t, 1H, J = 7.6 Hz), 1.21-1.32 (m, 2H), 1.38-1.44 (m, 2H), 1.49-1.60 (m, 2H), 1.72-1.77 (m, 1H), 2.66-2.69 (m, 1H), 3.39-3.54 (m, 1H), 7.20-7.22 (m, 2H), 7.34 (d, 1H, J = 7.2 Hz), 7.40 (d, 2H, J = 6.8 Hz), 7.47 (d, 1H, J = 8.0 Hz), 7.60 (m, 2H), 7.80 (d, 1H, J = 6.8 Hz). ^{13}C-NMR (100 MHz, $CDCl_3$): δ 166.7, 144.2, 139.4, 137.8, 132.6, 130.3, 129.4, 129.1, 128.4,

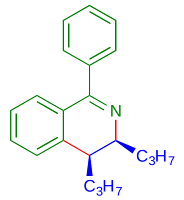

5am

128.2, 126.6, 60.9, 40.1, 29.9, 28.9, 20.8, 20.3, 14.6, 14.5. HRMS: calcd for $C_{21}H_{26}N^+$ 292.2060, found 292.2057.

***cis*-3-Ethyl-1,4-diphenyl-3,4-dihydroisoquinoline (5an):** Prepared from benzophenone imine (**1a**) and 1-phenyl-1-butyne by the general procedure A. Chromatography (1% ethyl acetate in hexane) gave **5an** as a light yellow liquid (75 mg). This product was contaminated with ~12% of the corresponding isoquinoline byproduct (generated by product decomposition during separation and purification), and the yield for **5an** was estimated to be 76% by ^{1}H NMR analysis (see Figure 31). ^{1}H-NMR (400 MHz, $CDCl_3$): δ 0.95 (t, 3H, J = 7.6 Hz), 1.79 (qd, 2H, J_1 = 7.6 Hz, J_2 = 2.4 Hz), 3.78 (q, 1H, J = 6.0 Hz), 4.07 (d, 1H, J = 5.6Hz), 7.14-7.19 (m, 5H), 7.27–7.30 (m, 2H), 7.37–7.42 (m, 2H), 7.46-7.49 (m, 4H), 7.73–7.75 (m, 2H). ^{13}C-NMR (100 MHz, $CDCl_3$): δ 167.5, 142.7, 139.4, 131.4, 130.4, 129.6, 129.3, 129.2, 128.8, 128.52, 128.48, 128.4, 128.3, 127.1, 126.9, 63.5, 46.5, 26.9, 11.7. HRMS: calcd for $C_{23}H_{22}N^+$ 312.1747, found 312.1732.

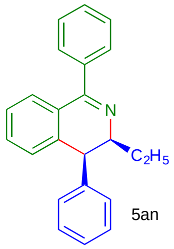

5an

***cis*-3-Butyl-1,4-diphenyl-3,4-dihydroisoquinoline (5ao):** Prepared from benzophenone imine (**1a**) and 1-phenyl-1-hexyne by the general procedure A. Chromatography (1% ethyl acetate in hexane) gave **5ao** as a light yellow liquid (78 mg). This product was contaminated with ~10% of the corresponding isoquinoline byproduct (generated by product decomposition during separation and purification), and the yield for **5ao** was estimated to be 75% by ^{1}H NMR analysis (see Figure 33). ^{1}H-NMR (400 MHz, $CDCl_3$): δ 0.94 (t, 3H, J = 7.6 Hz), 1.41-1.44 (m, 2H), 1.52-1.61 (m, 1H), 1.79 - 1.83 (m, 3H), 3.80 (dt, 1H, J_1 = 5.6 Hz, J_2 = 3.2 Hz), 4.07 (d, 1H, J = 6.0 Hz), 7.19-7.21 (m, 5H), 7.29 (d, 2H, J = 6.0 Hz), 7.37 (td, 2H, J_1 = 7.2 Hz, J_2 = 3.2 Hz), 7.48 (t, 3H, J = 6.0 Hz), 7.73-7.75 (m, 2H). ^{13}C-NMR (100 MHz, $CDCl_3$): δ 167.5, 142.8, 139.4, 139.3, 132.7, 131.4, 130.3, 129.7, 129.31, 129.25, 128.8, 128.5, 128.4, 127.1, 126.9, 61.8, 46.8, 33.7, 29.4, 23.1, 14.5. HRMS: calcd for $C_{25}H_{26}N^+$ 340.2060, found 340.2053.

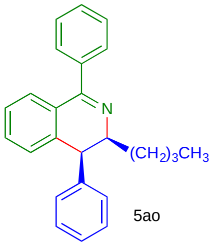

5ao

***cis*-6-chloro-1-(4-chlorophenyl)-3-ethyl-4-phenyl-3,4-dihydroisoquinoline (5ap):** Prepared from bis(4-chlorophenyl)methanimine and 1-phenyl-1-butyne by the general procedure A. Chromatography (1% ethyl acetate in hexane) gave **5ap** as a white solid (86 mg, 81%). ^{1}H-NMR (400 MHz, $CDCl_3$): δ 1.19 (t, 3H, *J* = 7.2 Hz), 1.79 (m, 2H), 3.67 (q, 1H, *J* = 7.6 Hz), 4.07 (d, 1H, *J* = 6.0 Hz), 7.13-7.16 (m, 2H), 7.21 -7.24 (m, 3H),7.29-7.33 (m, 3H), 7.48 (d, 2H, *J* = 8.4 Hz), 7.67 (d, 2H, *J* = 8.4 Hz). ^{13}C-NMR (100 MHz, $CDCl_3$): δ 165.4, 144.4, 138.1, 137.1, 137.0, 135.8, 131.4, 130.3, 129.3, 128.9, 128.7, 128.5, 127.3, 127.1, 126.6, 63.2, 46.2, 26.5, 11.5 HRMS: calcd for $C_{23}H_{20}Cl_2N^+$ 380.0973, found 380.0969.

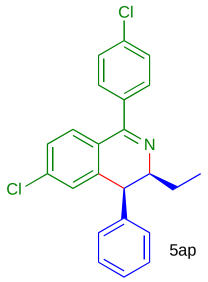

5ap

***cis*-1,3-diphenyl-4-(thiophen-2-yl)-3,4-dihydroisoquinoline (5aq):** Prepared from di-*p*-tolylmethanimine and 2-(phenylethynyl)thiophene by the general procedure A to give a 4:1 mixture of regioisomers. Further purification by chromatography (1% ethyl acetate in hexane) gave **5aq** as a white solid (66 mg, 66%). ^{1}H-NMR (400 MHz, $CDCl_3$): δ 4.70 (d, 1H, *J* = 5.6 Hz), 5.22 (d, 1H, *J* = 5.6 Hz), 6.34 (d, 1H, *J* = 3.2 Hz), 6.75-6.77 (m, 1H), 7.01 (d, 1H, *J* = 5.2 Hz), 7.29 -7.46 (m, 6H),7.55-7.59 (m, 7H), 7.89-7.92 (m, 2H). ^{13}C-NMR (100 MHz, $CDCl_3$): δ 167.9, 141.9, 141.6, 140.6, 139.1, 131.4, 129.6, 129.3, 128.5, 128.31, 128.28, 128.1, 127.9, 127.7, 126.9, 126.2, 126.1, 124.5, 65.6, 45.0 HRMS: calcd for $C_{25}H_{19}NS^+$ 366.1317, found 366.1317.

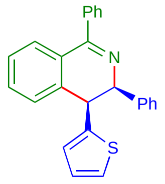

5aq, 66%

***cis*-6-Fluoro-1-(4-fluorophenyl)-3,4-diphenyl-3,4-dihydroisoquinoline (5ba):** Prepared from 4,4'-difluoro benzophenone imine and 1,2-diphenylethyne (**2a**) by the general procedure B. Chromatography (1% ethyl acetate in hexane) gave **5ba** as a white solid (101 mg, 91%). ^{1}H-NMR (400 MHz, $CDCl_3$): ^{1}H-NMR (400 MHz, $CDCl_3$): δ 4.30 (d, 1H, *J* = 6.0 Hz), 5.13 (d, 1H, *J* = 6.0 Hz), 6.57 (d, 2H, *J* = 8.8 Hz), 6.98-7.08 (m, 5H), 7.14-7.24 (m, 7H), 7.44-7.48 (m, 1H), 7.73-7.76 (m, 2H). ^{13}C-NMR (100 MHz, $CDCl_3$): δ 165.8, 165.5 (d, *J*(C,F) = 254.5 Hz), 165.2 (d, *J*(C,F) = 249.5 Hz), 144.7 (d, *J* = 8.1 Hz), 140.9, 136.9, 135.1 (d, *J* = 3.0 Hz), 131.2 (d, *J* = 9.1 Hz), 130.6 (d, *J* = 8.1 Hz), 129.6, 128.5, 128.1, 127.9, 127.1 (d, *J* = 7.0 Hz), 125.6 (d, *J* = 3.0 Hz), 115.7, 115.5, 114.5, 114.3, 66.1, 49.3. ^{19}F-NMR

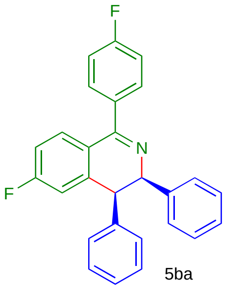

5ba

(376.3 MHz, $CDCl_3$): δ -29.7 (s, 1F), -33.9 (s, 1F). HRMS: calcd for $C_{27}H_{20}F_2N^+$ 396.1558, found 396.1559.

***cis*-6-Chloro-1-(4-chlorophenyl)-3,4-diphenyl-3,4-dihydroisoquinoline (5ca):** Prepared from

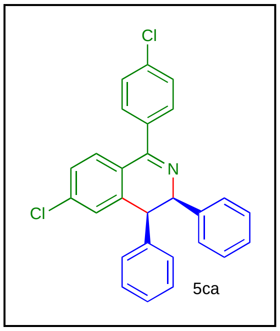

4,4'-dichlorobenzophenone imine and 1,2-diphenylethyne (**2a**) by the general procedure B. Chromatography (1% ethyl acetate in hexane) gave **5ca** as a white solid (104 mg, 87%). ^{1}H-NMR (400 MHz, $CDCl_3$):δ 4.36 (d, 1H, *J* = 5.6 Hz), 5.17 (d, 1H, *J* = 6.0 Hz), 6.60 (d, 2H, *J* = 7.2 Hz), 7.04-7.11 (m, 3H), 7.21-7.29 (m, 5H), 7.33-7.45 (m, 3H), 7.49 (d, 2H, *J* = 8.4 Hz), 7.73 (d, 2H, *J* = 8.4 Hz). ^{13}C-NMR (100 MHz, $CDCl_3$): δ 165.8, 143.3, 140.6, 137.3, 136.9, 136.6, 135.9, 130.4, 129.4, 129.3, 128.7, 128.6, 128.3, 127.9, 127.8, 127.6, 127.1, 126.9, 126.8, 66.1, 44.8. HRMS: calcd for $C_{27}H_{20}Cl_2N^+$ 428.0967, found 428.0973.

***cis*-6-methyl-3,4-diphenyl-1-(*p*-tolyl)-3,4-dihydroisoquinoline (5da):** Prepared from di-*p*-

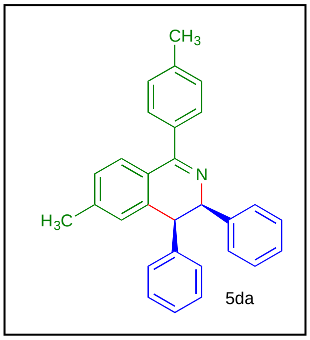

tolylmethanimine and diphenylacetylene by the general procedure A. Chromatography (0.5% ethyl acetate in hexane) gave **5da** as a white solid (75 mg, 70%). ^{1}H-NMR (400 MHz, $CDCl_3$): δ2.49 (S, 3H), 2.59 (m, 3H), 4.41 (d, 1H, *J* = 6.0 Hz), 5.28 (d, 1H, *J* = 6.0 Hz), 6.78 (d, 2H, *J* = 6.8 Hz), 7.10-7.19 (m, 3H), 7.25 -7.36 (m, 7H), 7.25-7.36 (m, 7H), 7.38-7.45 (m, 2H), 7.55 (d, 1H, *J* = 7.6 Hz), 7.85 (d, 2H, *J* = 8.0 Hz). ^{13}C-NMR (100 MHz, $CDCl_3$): δ167.6, 141.9, 141.8, 141.6, 139.5, 137.9, 136.6, 129.2, 129.0, 128.52, 128.46, 127.95, 127.91, 127.7, 126.9, 126.7, 126.5, 66.4, 49.4, 21.7, 21.6. HRMS: calcd for $C_{29}H_{25}N^+$ 388.2065, found 388.2073.

***cis*-3,4-Diphenyl-7-trifluoromethyl-1-(3-trifluoromethylphenyl)-3,4-dihydroisoquinoline**

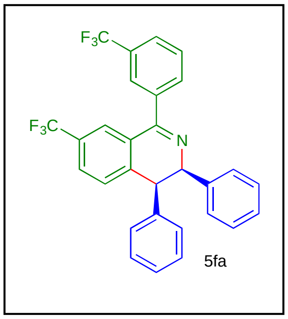

(5fa): Prepared from 3,3'-bis(trifluoro-methyl) benzophenone imine and 1,2-diphenylethyne (**2a**) by the general procedure B. Chromatography (1% ethyl acetate in hexane) gave **5fa** as a white solid (129 mg, 93%). ^{1}H-NMR (400 MHz, $CDCl_3$): δ 4.45 (d, 1H, *J* = 6.4 Hz), 5.22 (d, 1H, *J* = 6.0 Hz), 6.60 (d, 2H, *J* = 5.6 Hz), 7.02-7.11 (m, 3H), 7.24-7.26 (m, 5H), 7.47 (d, 1H, *J* = 8.0 Hz), 7.66-7.83 (m, 4H), 7.96 (d, 1H, *J* = 8.0 Hz),

8.11 (s, 1H). ^{13}C-NMR (100 MHz, $CDCl_3$): δ 165.4, 148.9, 145.9, 145.3, 140.5 (q, J = 28.1 Hz), 139.9 (q, *J* = 29.2 Hz), 133.9, 132.1, 129.9(q, J = 10.9 Hz), 129.4, 129.3, 129.0, 128.3, 128.0, 127.9, 127.1 (q, *J* = 10.1 Hz), 125.9 (q, *J* = 3.0 Hz), 125.3 (q, *J*(C,F) = 272.6 Hz), 125.1 (q, *J*(C,F) = 272.6 Hz), 124.4 (q, *J* = 3.0 Hz), 119.9, 119.7, 66.1, 48.6. ^{19}F-NMR (376.3 MHz, $CDCl_3$): δ -15.0 (s, 1F), -14.9 (s, 1F). HRMS: calcd for $C_{29}H_{19}F_6NNa^+$ 518.1314, found 518.1327.

***cis*-1-(3-Methoxyphenyl)-3,4-diphenyl-7-trifluoromethyl-3,4-dihydroisoquinoline (5ga):**

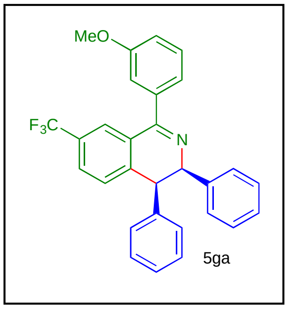

Prepared from 3-trifluoromethyl-3'-methoxy benzophenone imine and 1,2-diphenylethyne (**2a**) by the general procedure B. Chromatography (1% ethyl acetate in hexane) gave **5ga** as a light yellow liquid (112 mg, 88%). ^{1}H-NMR (400 MHz, $CDCl_3$): δ 3.86 (s, 3H), 4.39 (d, 1H, *J* = 6.0 Hz), 5.17 (d, 1H, *J* = 6.0 Hz), 6.60 (d, 2H, *J* = 6.8 Hz), 7.00-7.10 (m, 4H), 7.22-7.24 (m, 5H), 7.31 (d, 2H, *J* = 4.8 Hz), 7.39 (t, 2H, *J* = 8.4 Hz), 7.67 (d, 1H, J = 8.0 Hz), 7.79 (s, 1H). ^{13}C-NMR (100 MHz, $CDCl_3$): δ 166.3, 159.9, 145.2, 140.5, 139.5, 136.5, 131.2, 131.1, 130.5, 130.3 (q, *J* = 14.1 Hz), 128.9, 128.6, 128.3, 128.5, 127.9, 127.7, 127.4 (q, *J* = 8.1 Hz), 124.9 (q, *J* = 4.0 Hz), 122.5 (q, *J*(C,F) = 271.6 Hz), 121.3, 116.0, 114.2, 65.9, 55.5, 48.8. ^{19}F-NMR (376.3 MHz, $CDCl_3$): δ -62.4 (s, 1F). HRMS: calcd for $C_{29}H_{23}F_3NO^+$ 458.1726, found 458.1732.

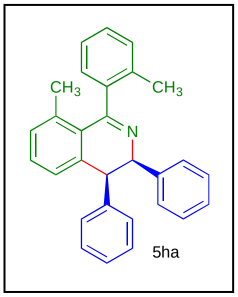

***cis*-8-Methyl-3,4-diphenyl-1-(*o*-tolyl)-3,4-dihydroisoquinoline (5ha):** Prepared from di-*o*-tolylmethyleneamine and 1,2-diphenylethyne (**2a**) by the general procedure B. Chromatography (1% ethyl acetate in hexane) gave **5ha** as a white solid (83 mg). This product was contaminated with ~15% of the corresponding isoquinoline byproduct (generated via product decomposition during separation and purification), and the yield for **5ha** was estimated to be 66% by ^{1}H NMR analysis (see Figure 54). ^{1}H-NMR (400 MHz, $CDCl_3$): δ 1.81 (s, 3H), 2.46 (s, 3H), 4.31 (d, 1H, *J* = 5.6 Hz), 5.14 (d, 1H, *J* = 5.6 Hz), 6.68 (d, 2H, *J* = 6.8 Hz), 6.97 (t, 4H, *J* = 7.6Hz), 7.11-7.14 (m, 2H), 7.21-7.23 (m, 5H), 7.27-7.29 (m, 2H), 7.33 (d, 2H, *J* = 6.8 Hz). ^{13}C-NMR (100 MHz, $CDCl_3$): δ 167.7, 143.3, 141.9, 141.8, 137.8, 136.8, 131.6, 131.4, 130.9, 130.4, 130.0, 129.7, 128.6, 128.5, 128.4, 128.2, 128.1, 127.8, 126.8, 126.7, 126.2, 125.9, 65.4, 50.1, 22.4, 20.8. HRMS: calcd for $C_{29}H_{26}N^+$ 388.2060, found 388.2073.

***cis*-1-Butyl-3,4-diphenyl-3,4-dihydroisoquinoline (5ia):** Prepared from 1-phenyl-pentylideneamine and 1,2-diphenylethyne (**2a**) by the general procedure B. Chromatography (1% ethyl acetate in hexane) gave **5ia** as a light yellow liquid (80 mg, 84%). ^{1}H-NMR (400 MHz, $CDCl_3$): δ 1.02 (t, 3H, *J* = 7.2 Hz), 1.53 (sext, 2H, *J* = 7.6 Hz), 1.78-1.93 (m, 2H), 2.91-3.05 (m, 2H), 4.26 (d, 1H, *J* = 6.4 Hz), 5.05 (d, 1H, *J* = 6.4 Hz), 6.48 (d, 2H, *J* = 7.2 Hz), 6.98-7.08 (m, 3H), 7.16-7.26 (m, 6H), 7.38-7.45 (m, 2H), 7.72 (d, 1H, *J* = 6.8 Hz). ^{13}C-NMR (100 MHz, $CDCl_3$): δ 168.3, 141.9, 140.4, 138.1, 131.4, 129.8, 129.5, 128.9, 128.5, 128.0, 127.8, 127.6, 126.8, 126.6, 125.5, 65.3, 48.9, 36.0, 29.7, 23.1, 14.3. HRMS: calcd for $C_{25}H_{26}N^+$ 340.2060, found 340.2049.

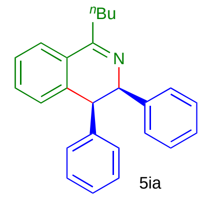

5ia

1-*tert*-Butyl-3,4-diphenyl-3,4-dihydroisoquinoline (5ja): Prepared from 2,2-dimethyl-1-phenyl-propylideneamineand 1,2-diphenylethyne (**2a**) by the general procedure B. Chromatography (1% ethyl acetate in hexane) gave **5ja** as a yellow liquid (86 mg, 90%). ^{1}H-NMR (400 MHz, $CDCl_3$): δ 1.54 (s, 9H), 4.20 (d, 1H, *J* = 6.0 Hz), 4.92 (d, 1H, *J* = 6.0 Hz), 6.43 (d, 2H, *J* = 7.2 Hz), 6.95 -7.06(m, 3H), 7.21-7.31 (m, 6H), 7.36-7.41 (m, 2H), 7.97 (d, 1H, *J* = 7.6 Hz). ^{13}C-NMR (100 MHz, $CDCl_3$): δ 173.3, 142.6, 142.1, 137.5, 130.4, 129.9, 128.9, 128.5, 128.0, 127.4, 126.95, 126.85, 126.7, 126.4,64.8, 49.3, 40.2, 30.4. HRMS: calcd for $C_{25}H_{26}N^+$ 340.2060, found 340.2060.

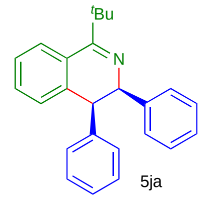

5ja

***cis*-1-Methyl-3,4-diphenyl-7-(trifluoromethyl)-3,4-dihydro-isoquinoline (5ka):** Prepared from 1-(3-trifluoromethyl-phenyl)-ethylideneamine and 1,2-diphenylethyne (**2a**) by the general procedure B. Chromatography (1% ethyl acetate in hexane) gave **5ka** as a yellow liquid (90 mg, 88%). ^{1}H-NMR (400 MHz, $CDCl_3$): δ 2.71 (s, 3H), 4.35 (d, 1H, *J* = 6.4 Hz), 5.09 (d, 1H, *J* = 6.4 Hz), 6.51 (d, 2H, *J* = 7.2 Hz), 7.06-7.17 (m, 5H), 7.24-7.26 (m, 3H), 7.35 (d, 1H, *J* = 7.6 Hz), 7.69 (d, 1H, *J* = 7.6 Hz), 7.99 (s, 1H). ^{13}C-NMR (100 MHz, $CDCl_3$): δ 164.1, 143.7, 140.6, 136.9, 130.6(q, *J* = 32.6 Hz), 129.9, 129.4, 129.1, 128.1, 127.93, 127.91, 127.87, 127.8, 126.9, 126.8, 125.3 (q, *J*(C, F) = 270.5 Hz), 123.6 (q, *J* = 5.0 Hz),

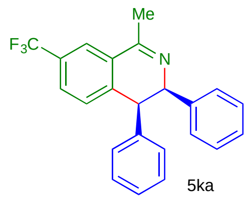

5ka

122.5 (q, J = 3.7 Hz), 65.1, 48.5, 23.4. ^{19}F-NMR (376.3 MHz, $CDCl_3$): δ -62.4 (s, 1F). HRMS: calcd for $C_{23}H_{19}F_3N^+$ 366.1464, found 366.1457.

***cis*-1-Butyl-3,4-diphenyl-7-trifluoromethyl-3,4-dihydro-isoquinoline (5la):** Prepared from 1-(3-trifluoromethyl-phenyl)-butylideneamine and 1,2-diphenylethyne (**2a**) by the general procedure B. Chromatography (1% ethyl acetate in hexane) gave **5la** as a yellow liquid (99 mg, 87%). ^{1}H-NMR (400 MHz, $CDCl_3$): δ 0.99 (t, 3H, J = 7.6 Hz), 1.47 (sext, 2H, J = 7.6 Hz), 1.78-1.84 (m, 2H), 2.94-2.98 (m, 2H), 4.29 (d, 1H, J = 6.4 Hz), 5.02 (d, 1H, J = 6.4 Hz), 6.42 (d, 2H, J = 7.2 Hz), 6.96-7.05 (m, 3H), 7.12-7.24 (m, 5H), 7.31 (d, 1H, J = 6.4 Hz), 7.63 (d, 1H, J = 6.4 Hz), 7.93 (s, 1H). ^{13}C-NMR (100 MHz, $CDCl_3$): δ 166.9, 144.2, 141.1, 137.1, 130.4, 130.0 (q, J = 32.2 Hz), 129.8, 129.7, 129.5, 128.3, 128.2, 127.8, 127.0, 126.9, 125.5 (q, J(C,F) = 272.6 Hz), 122.8, 122.1 (q, J = 4.0 Hz), 64.9, 48.6, 35.7, 29.2, 22.1, 14.2. ^{19}F-NMR (376.3 MHz, $CDCl_3$): δ -15.1 (s, 1F). HRMS: calcd for $C_{26}H_{25}F_3N^+$ 408.1934, found 408.1923.

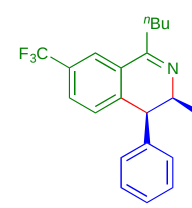

5la

***cis*-1-*tert*-Butyl-3,4-diphenyl-7-trifluoromethyl-3,4-dihydro-isoquinoline (5ma):** Prepared from 2,2-dimethyl-1-(3-trifluoromethylphenyl)-propan-1-imine and 1,2-diphenylethyne (**2a**) by the general procedure B. Chromatography (1% ethyl acetate in hexane) gave **5ma** as a yellow liquid (99 mg, 87%). ^{1}H-NMR (400 MHz, $CDCl_3$): δ 1.60 (s, 9H), 4.32 (d, 1H, J = 6.0 Hz), 4.97 (d, 1H, J = 6.0 Hz), 6.46 (d, 2H, J = 7.2 Hz), 7.02 (d, 2H, J = 6.8Hz), 7.09 (d, 1H, J = 6.0 Hz), 7.28 -7.31 (m, 5H), 7.36 (d, 1H, J = 7.6 Hz), 7.61 (d, 1H, J = 7.2 Hz), 8.18 (s, 1H). ^{13}C-NMR (100 MHz, $CDCl_3$): δ 172.1, 145.7, 141.6, 136.3, 131.4 (q, J = 32.7 Hz), 130.6, 129.5, 129.3, 128.9, 128.2, 127.9, 127.4, 126.6, 125.4 (q, J(C,F) = 270.7 Hz), 124.6 (q, J = 10.6 Hz), 123.3 (q, J = 3.0 Hz), 123.5 (q, J = 3.0 Hz), 64.3, 48.8, 39.9, 30.1. ^{19}F-NMR (376.3 MHz, $CDCl_3$): δ -62.4 (s, 1F). HRMS: calcd for $C_{26}H_{25}F_3N^+$ 408.1934, found 408.1932.

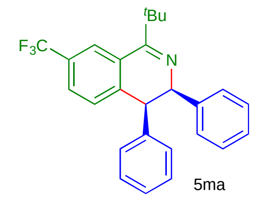

5ma

***cis*-1-Methyl-3,4-diphenyl-6-trifluoromethyl-3,4-dihydro-isoquinoline(5na):** Prepared from 1-(4-trifluoromethyl-phenyl)ethanimine and 1,2-diphenylethyne (**2a**) by the general procedure B. Chromatography (1%) gave **5na** as a light yellow liquid (91 mg, 89%). ^{1}H-NMR (400 MHz, $CDCl_3$): δ 2.65 (s, 3H), 4.30 (d, 1H, J = 6.4 Hz), 5.02 (d, 1H, J = 6.4 Hz), 6.45 (d, 2H, J = 7.2 Hz), 6.99 (t, 2H, J = 6.8 Hz), 7.06-7.11 (m, 3H),

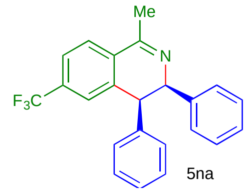

5na

7.19-7.24 (m, 3H), 7.47 (s, 1H), 7.66 (d, 1H, J = 6.8 Hz), 7.78 (d, 1H, J = 8.4 Hz). ^{13}C-NMR (100 MHz, $CDCl_3$): δ 164.4, 140.9, 137.0, 133.2(q, J = 32.5 Hz), 132.9, 132.3, 129.6, 128.3, 128.1, 127.9, 127.1, 126.9, 126.1, 125.6 (q, J = 3.0 Hz), 125.2 (q, J(C,F) = 272.6 Hz), 124.8 (q, J = 3.0 Hz), 65.4, 48.9, 23.6. ^{19}F-NMR (376.3 MHz, $CDCl_3$): δ -63.3 (s, 1F). HRMS: calcd for $C_{23}H_{19}F_3N^+$ 366.1464, found 366.1458.

***cis*-1-tert-Butyl-3,4-diphenyl-6-(trifluoromethyl)-3,4-dihydro-isoquinoline (5oa):** Prepared

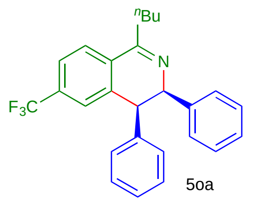

5oa

from 2,2-dimethyl-1-(4-trifluoromethylphenyl)propan-1-imine and 1,2-diphenylethyne (**2a**) by the general procedure B. Chromatography (1% ethyl acetate in hexane) gave **5oa** as a light yellow liquid (98 mg, 86%). ^{1}H-NMR (400 MHz, $CDCl_3$): δ 1.02 (t, 3H, J = 7.2 Hz), 1.50 (sext, 2H, J = 7.6 Hz), 1.76-1.94 (m, 2H), 2.90-3.08 (m, 2H), 4.33 (d, 1H, J = 6.4 Hz), 5.05 (d, 1H, J = 6.4 Hz), 6.46 (d, 2H, J = 7.6 Hz), 6.99 (t, 2H, J = 7.2 Hz) 7.07 (t, 1H, J = 7.6 Hz), 7.16-7.18 (m, 2H), 7.21 -7.27(m, 3H), 7.51 (s, 1H), 7.68 (d, 1H, J = 8.0 Hz), 7.81 (d, 1H, J = 8.0 Hz). ^{13}C-NMR (100 MHz, $CDCl_3$): δ 167.2, 141.3, 141.2, 137.0, 133.3, 132.9, 132.0 (q, J = 30.6 Hz), 129.7, 128.4, 128.1, 127.8, 126.9, 125.8 (q, J = 4.1 Hz), 124.8 (q, J = 4.2 Hz), 122.6, 119.8 (q, J(C,F) = 272.6 Hz), 65.1, 48.6, 35.7, 29.3, 23.0, 14.2.^{19}F-NMR (376.3 MHz, $CDCl_3$): δ -14.7 (s, 1F). HRMS: calcd for $C_{26}H_{25}F_3N^+$ 408.1920, found 408.1934.

(*E*)-2-[2-(1-Phenylhex-1-enyl)-phenyl]-pyridine (8): Prepared from 2-phenylpyridine and 1-

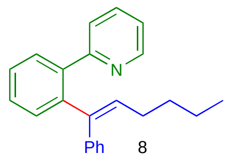

8

phenyl-1-hexyne by the general procedure A for redox-neutral [4+2] imine/alkyne annulation. Chromatography (1% ethyl acetate in hexane) gave **8** as a yellow liquid (84 mg, 83%). ^{1}H-NMR (400 MHz, $CDCl_3$): δ 0.82 (t, 3H, J = 6.8 Hz), 1.24-1.30 (m, 4H), 2.16 (dt, 2H, J_1= J_2 = 7.2 Hz), 5.70 (t, 1H, J = 7.2 Hz), 6.97 (d, 2H, J = 6.4 Hz), 7.12-7.30 (m, 4H), 7.28 (d, 1H, J = 7.6 Hz), 7.37-7.40 (m, 3H), 7.47-7.51(m, 2H), 8.51 (d, 1H, J = 5.2 Hz). ^{13}C-NMR (100 MHz, $CDCl_3$): δ 160.0, 148.9, 143.3, 140.6, 140.4, 140.3, 135.2, 134.1, 130.8, 130.0, 129.7, 127.9, 127.4, 127.1, 126.2, 124.4, 121.1, 31.9, 29.3, 22.3, 13.9. HRMS: calcd for $C_{23}H_{24}N^+$ 314.1903, found 314.1914.

***cis*-1,3,4-Triphenyl-1,2,3,4-tetrahydroisoquinoline (9a):** Synthesized based on an analogous

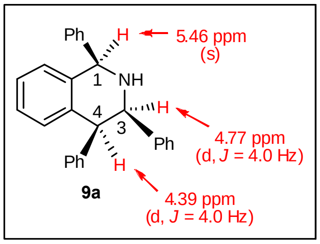

hydride reduction procedure reported by the groups of Bergman and Ellman.[2] Into a 20 mL scintillation vial equipped with a magnetic stir bar was placed *cis*-1,3,4-triphenyl-3,4-dihydroisoquinoline (**5a**, 0.28mmol, 1.00 equiv**)**, diphenylphosphate (0.58mmol, 2.10 equiv), and THF (4 mL). The mixture was stirred at room temperature for 16 h, transferred to a fume hood, and added to a suspension of tetramethylammonium triacetoxyborohydride (1.67 mmol, 6.00 equiv) in THF (10 mL) in a 50 mL round bottom flask immersed in a 0 °C bath. The mixture was stirred at 0 °C for 2 h before warming to room temperature and stirred for another 2 h. The reaction was quenched sequentially with water (1 mL) and saturated aqueous NH_4Cl (1 mL), and then was added dropwise with 2 mL of 2.0 M NaOH until PH 11 was reached. The mixture was extracted with a mixed solution of Hexane:EtOAC:Et_3N (400:25:3, 3x30mL) and the organic layers were combined, filtered through Celite, and evaporated under reduced pressure to remove all volatiles. The crude product was further purified by flash column chromatography with neutral Alumina and 1% ethyl acetate in hexane to give a 20:1 inseparable mixture of **9a** and **9b** as a white solid (87.4 mg, 87% combined yield, diastereoselectivity determined by ^{1}H NMR analysis). ^{1}H-NMR for **9a** (400 MHz, $CDCl_3$): δ 4.39 (d, 1H, *J* = 4.0 Hz), 4.77 (d, 1H, *J* = 4.0 Hz), 5.46 (s, 1H), 7.01-7.06 (m, 5H), 7.19–7.23 (m, 9H),7.48 (d, 1H, *J* = 7.2 Hz), 7.53-7.56 (m, 2H), 7.71 (d, 2H, *J* = 6.8 Hz). ^{13}C-NMR for **9a** (100 MHz, $CDCl_3$): δ 144.3, 142.3, 141.9, 138.9, 138.7, 130.9, 130.8, 129.4, 128.7, 127.94, 127.88, 127.6, 127.14, 127.08, 126.9, 126.8, 126.3, 126.1, 65.0, 63.4, 52.0. HRMS: calcd for $C_{27}H_{24}N^+$ 362.1909, found 362.1910.

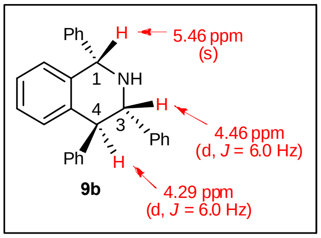

The following ^{1}H NMR signals could be identified for the aliphatic protons in the diastereomer **9b** (400 MHz, $CDCl_3$): δ 4.46 (d, 1H, *J* = 6.0 Hz), 5.29 (d, 1H, *J* = 6.0 Hz), 5.46 (s, 1H, overlapping with a singlet from **9a**). The relatively larger H(3)-H(4) coupling (compared to **9a**) is consistent with a *trans*-3,4-disubstitution stereochemistry.

***cis*-1-(2-butylphenyl)-3,4-diphenyl-3,4-dihydroisoquinoline (12):** In to a 4 dram vial *cis*-1,3,4-triphenyl-3,4-dihydroisoquinoline (**5aa**, 0.20 mmol), potassium butyltrifluoroborate (0.60 mmol), $[RhCp^{*}Cl_2]_2$ (0.008 mmol), $AgSbF_6$ (0.032 mmol), AgF (0.78 mmol) and DCE 3.0 mL were added and the vial was sealed with a silicone-lined screw-cap, transferred out of the glovebox, and stirred at 100 °C for 24 hours. After the reaction mixture was cooled to room temperature, all volatile materials were removed under reduced pressure. Further purification was achieved by flash-column chromatography using neutral alumina. Chromatography (1% ethyl acetate in hexane) gave **12** as a colorless liquid (55 mg, 68%). ^{1}H-NMR (400 MHz, $CDCl_3$): δ 0.83 (t, 3H, *J* = 7.6 Hz), 1.23 (q, 2H, *J* = 7.2 Hz), 1.54 (bs, 2H), 2.54 (bs, 2H), 4.38 (d, 1H, *J* = 10.0 Hz), 5.17 (bs, 1H), 7.01 (d, 2H, *J* = 7.6 Hz), 7.01 (d, 1H, *J* = 7.6 Hz),7.08 (d, 1H, *J* = 7.6 Hz), 7.17-7.26 (m, 9H), 7.29-7.34 (m, 4H), 7.34-7.41 (m, 3H). ^{13}C-NMR (100 MHz, $CDCl_3$): δ 167.6, 142.82, 142.81, 140.8, 139.2, 138.6, 131.4, 129.4, 129.3, 129.19, 129.18, 128.9, 128.8, 128.5, 128.3, 128.1, 127.8, 127.7, 127.2, 126.8, 125.8, 68.3, 49.9, 33.2, 29.7, 22.7, 13.9. HRMS: calcd for $C_{31}H_{29}N^{+}$ 416.2378, found 416.2391.

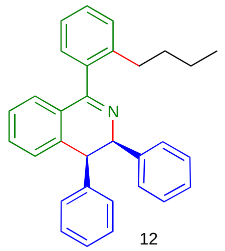

12

1-(2-bromophenyl)-3,4-diphenylisoquinoline (13): In to a 4 dram vial *cis*-1,3,4-triphenyl-3,4-dihydroisoquinoline (**5aa**, 0.28 mmol), NBS (0.33 mmol), $[RhCp^{*}Cl_2]_2$ (0.014 mmol), $AgSbF_6$ (0.056 mmol), PivOH (0.31 mmol) and DCE 2.0 mL were added and the vial was sealed with a silicone-lined screw-cap, transferred out of the glovebox, and stirred at 80 °C for 9 hours. After the reaction mixture was cooled to room temperature, all volatile materials were removed under reduced pressure. Further purification was achieved by flash-column chromatography using neutral alumina. Chromatography (1% ethyl acetate in hexane) gave **13** as a colorless liquid (90 mg, 74%). ^{1}H-NMR (400 MHz, $CDCl_3$): δ 7.19-7.24 (m, 3H), 7.32-7.34 (m, 1H), 7.37 -7.46 (m, 7H),7.49-7.55 (m, 2H), 7.56-7.65 (m, 2H), 7.74–7.81 (m, 3H). ^{13}C-NMR (100 MHz, $CDCl_3$): δ 159.1, 149.7, 140.7, 140.5, 137.3, 136.5, 132.9, 131.7, 131.4, 130.5, 130.2, 129.9, 128.4, 128.2, 127.6, 127.4, 127.3, 127.2, 127.0, 126.8, 126.0, 125.8, 123.4. HRMS: calcd for $C_{27}H_{18}NBr^{+}$ 436.0701, found 436.0719.

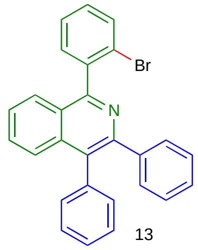

13

Supplementary References:

1) How, G.; Gosselin, F.; Li, W.; McWilliams, J. C.; Sun, Y.; Weisel, M.; O'Shea, P. D.; Chen, C.; Davies, I.W.; Zhang, X. *J. Am. Chem. Soc*. **2009**, *131*, 9882.

2) Duttwyler, S.; Chen, S.; Takase, M. K.; Wiberg, K. B.; Bergman, R. G.; Ellman, J. A. *Science***2013**, *339*, 678.

3) Wang, H.; Yu, S.; Qi, Z.; Li, X. *Org. Lett.***2015**, *17*, 2812.

4) Schroeder, N.; Wencel-Delord, J.; Glorius, F. *J. Am. Chem. Soc.***2012**, *134*, 8298.

5) He, R; Huang, Z.-T; Zheng, Q.-Y; Wang, C. *Angew. Chem. Int. Ed.* **2014**, *53*, 4950.

6) Sun, Z.-M.; Chen, S.-P.; Zhao, P. *Chem. Eur. J.***2010**, *16*, 2619.
